# Large-scale discovery and annotation of substructure patterns in mass spectrometry profiles

**DOI:** 10.1038/s41467-026-75038-0

**Published:** 2026-07-06

**Authors:** Laura Rosina Torres Ortega, Jonas Dietrich, Joe Wandy, Hans Mol, Justin J. J. van der Hooft

**Affiliations:** 1 Bioinformatics Group, Wageningen University & Research, Wageningen, the Netherlands; 2 Wageningen Food Safety Research – part of Wageningen University & Research, Wageningen, the Netherlands; 3 College of Medical, Veterinary & Life Sciences, University of Glasgow, Glasgow, UK; 4 Department of Biochemistry, University of Johannesburg, Johannesburg, South Africa

**Keywords:** Metabolomics, Data mining, Metabolomics

## Abstract

Untargeted mass spectrometry can detect thousands of molecules at once, potentially offering powerful insights into complex samples. However, the increasing scale of experimental datasets and spectral libraries limits our ability to extract and annotate structural information to allow for interpretation. Here, we present the software tool MS2LDA 2.0 that helps to address this gap by identifying recurring fragmentation patterns (Mass2Motifs) that can reflect shared chemical substructures. We introduce automated annotation support through Mass2Motif Annotation Guidance (MAG) that provides suggestions to interpret detected patterns. Our unsupervised pattern mining tool enables the study of much larger datasets with up to 14 times faster analysis than its predecessor. We demonstrate the utility of MS2LDA 2.0 and MAG in applications such as detecting pesticide-related substructures and exploring unknown fungal compounds. Together, these advances make it easier to uncover meaningful chemical patterns in complex data.

## Abstract (web summary)

MS2LDA 2.0 enables fast, scalable analysis of mass spectrometry data and improves automated interpretation of molecular patterns, helping researchers more easily identify chemical substructures and discover new molecules.

## Introduction

Mass Spectrometry (MS) is an essential tool used across diverse fields, such as metabolomics, food safety, natural products, and drug discovery. Its high sensitivity and specificity make it essential for identifying and characterizing small molecules and biomolecules in complex samples^1^. Tandem mass spectrometry (MS/MS), a key advancement first developed in proteomics^2^, has transformed metabolomics since its introduction into the field^3^. By fragmenting detected molecular ions into smaller ions, MS/MS provides richer structural information. Furthermore, MS/MS has become critical in organizing^4^ and annotating^5^ mass spectrometry-based metabolomics profiles.

Despite advances in instrumentation and the rapid growth of public MS/MS libraries^6,7^, a substantial fraction, often more than 85%, of MS/MS spectra remains unannotated, depending on the sample type^8^. To close this gap, several computational approaches have been proposed, ranging from propagating structural annotations through molecular networking^4^, via fragmentation tree-based annotation methods like SIRIUS^9^, to deep learning methods matching spectra to structures such as DiffMS^10^, MIST^11^, and MSNovelist^12^. Despite these efforts, annotating metabolites remains difficult due to their complex fragmentation behavior, limited mass spectral libraries, and the diversity of chemical space^13,14^.

An alternative route to annotate metabolite features is to predict their chemical compound class terms with tools such as MolNetEnhancer^15^, CANOPUS^16^, which assigns spectra to classes within a predefined ontology, or through analog search to library spectra with predefined classes with tools like MS2Query^17^. Such class ontology-based annotations could be combined with maximum common subgraph analysis to derive shared substructures. However, these ontology-driven strategies are limited by the resolution of the class hierarchy and often return trivial scaffolds for structurally diverse classes, while many relevant substructure moieties or functional groups, such as glycosylations, phosphate groups or indole rings, can recur across multiple chemical classes, and therefore not captured based on this class-prediction logic. As such, unsupervised substructure-level discovery approaches such as MS2LDA that operate directly on the mass fragmentation spectra are complementary to both full-structure annotation and chemical compound class prediction. Especially when full structural elucidation is not feasible, identifying substructures can still provide meaningful chemical insights, such as detecting novel bioactive scaffolds or revealing new relationships between molecules.

Substructure-based tools analyze fragmentation patterns to detect molecular building blocks. One of the first of these was MS2LDA^18^, an unsupervised method based on topic modeling, which identifies recurrent patterns of fragments and losses, termed Mass2Motifs (see Supplementary Fig. S1). These Mass2Motifs are manually annotated (for example, a neutral loss of 162 is associated with a hexose substitution, e.g., glucose), and because such substructures recur across molecules, the same Mass2Motif can be present in multiple spectra. The unsupervised nature of MS2LDA makes it an exploratory data analysis tool that enables the discovery of such recurring patterns without relying on predefined substructure libraries, making it particularly valuable for exploratory data analysis.

Due to its unsupervised nature, MS2LDA has already facilitated substantial novel biological discoveries. For instance, its application revealed the key role of homogentisic acid as a precursor for biosynthesis and biotransformation of the antibiotic trimethoprim in pigmented bacteria, providing new opportunities for treatment of infections^19^. In another study, MS2LDA identified distinct Mass2Motis in polyketides with cytotoxic and antitumor properties, discriminating key properties that may influence their antitumor properties, and facilitating focusing on novel structural analogs amongst the currently 500 known acetogenins^20^. Finally, MS2LDA has been an integral part of the unsupervised workflow that resolved the first siderophore hydrolysis degradation mechanism of amphibactin in co-cultured marine bacteria^21^.

Since its conception in 2015, MS2LDA has grown into a widely adopted tool for substructure discovery in MS/MS data. A web application was introduced to explore Mass2Motifs^22^, and a database with manually annotated and curated Mass2Motifs, named MotifDB, was built to help researchers reuse and share substructure annotations^23^. MS2LDA was also integrated with other metabolomics tools, such as the popular GNPS platform^4^, allowing researchers to find relationships between molecules based on shared substructures and not only based on mass spectral similarity^15,24^. These developments have made MS2LDA a widespread tool, for instance in natural product discovery^25^, in studying microbial diversity^26,27^, and in xenobiotic screening^28^.

Despite these advancements, several key challenges remain. First, extracting Mass2Motifs from large datasets is computationally demanding, making it unfeasible for some datasets (over 20,000 spectra). Second, the interpretation of Mass2Motifs still depends heavily on manual annotation by human experts. While other tools such as MESSAR^29^ automate parts of this process, they rely on predefined substructures and ignore peak intensities. Third, MS2LDA lacks integration with widely used Python-based metabolomics frameworks like matchms^30,31^.

To address these limitations, MS2LDA 2.0 introduces a completely redesigned and scalable framework for substructure exploration in mass spectrometry. Alongside a more computationally efficient and performant topic modeling algorithm, it brings several key innovations: an automated Mass2Motif Annotation Guidance (MAG) system powered by Spec2Vec^32^, a fully rebuilt, queryable MotifDB leveraging MassQL^33^, full integration with Python-based metabolomics tools through matchms, and a completely redesigned interactive visualization app (online available to visualize MS2LDA runs at: https://ms2lda.org/viz/).

In this study, we validate MS2LDA 2.0 through benchmarking of manually curated MotifSets and in a large spectral library of standards, demonstrate its application in experimental data by analyzing spiked-in pesticides in food, uncover potentially unknown metabolite fungal metabolites, a bacterial siderophore degradation re-investigation and demonstrate its scalable application in a large MS/MS dataset. We also highlight its integration into modern mass spectrometry analysis frameworks.

## Results

The completely redesigned unsupervised substructure discovery tool MS2LDA 2.0 handles large datasets. However, this results in larger collections of mass spectral patterns, Mass2Motifs, that need to be structurally annotated to use them to their full potential. To streamline this process, we developed the automated Mass2Motif Annotation Guidance (MAG) by Spec2Vec^32^ (see full explanation in the Supplementary Notes 3.1 and Supplementary Fig. S3-S5), which for now has been trained with positive ionization mode data, and the Mass2Motif-matching functionality against MotifDB (see Supplementary Methods 4.3.3).

### Benchmarking of Mass2Motif Annotation Guidance using MotifDB

During the redesign of MS2LDA, we identified the manual annotation of Mass2Motifs as a major bottleneck. In the original MS2LDA workflow, manually annotating 30 Mass2Motifs, was a laborious task that required several days of expert time. Because for large datasets, MS2LDA 2.0 can generate more than a thousand Mass2Motifs of which hundred or more can be relevant, manual structural annotation has become a substantial practical limitation. To address this challenge, we developed Mass2Motif Annotation Guidance (MAG), an automatic procedure that supports Mass2Motif annotation by using spectral similarity scoring with Spec2Vec and additional refinement steps (see Methods Motif Annotation Guidance (MAG) section). MAG suggests candidate structures that are likely to contain the Mass2Motif of interest, allowing users to recognize the co-occurring substructure that the Mass2Motif represents. When relevant structures are found, this substantially accelerates and simplifies the structural annotation process.

To benchmark MAG, we used Mass2Motif datasets that were previously manually annotated and published as three positive ionization mode MotifSets in MotifDB: urine-derived^28^, GNPS library-derived^23^, and MassBank library-derived^23^ Mass2Motifs. The evaluation was based on the overlap of MACCS fingerprints between manually and automatically recommended structures using a motif fingerprint (see Methods: Motif fingerprints). To benchmark, we introduce a substructure overlap score, hereafter termed SOS (Supplementary Notes 3.1.3), where an SOS of 1 represents a complete fingerprint overlap between the automated recommendation and the manual annotation.

**Fig. 1:**
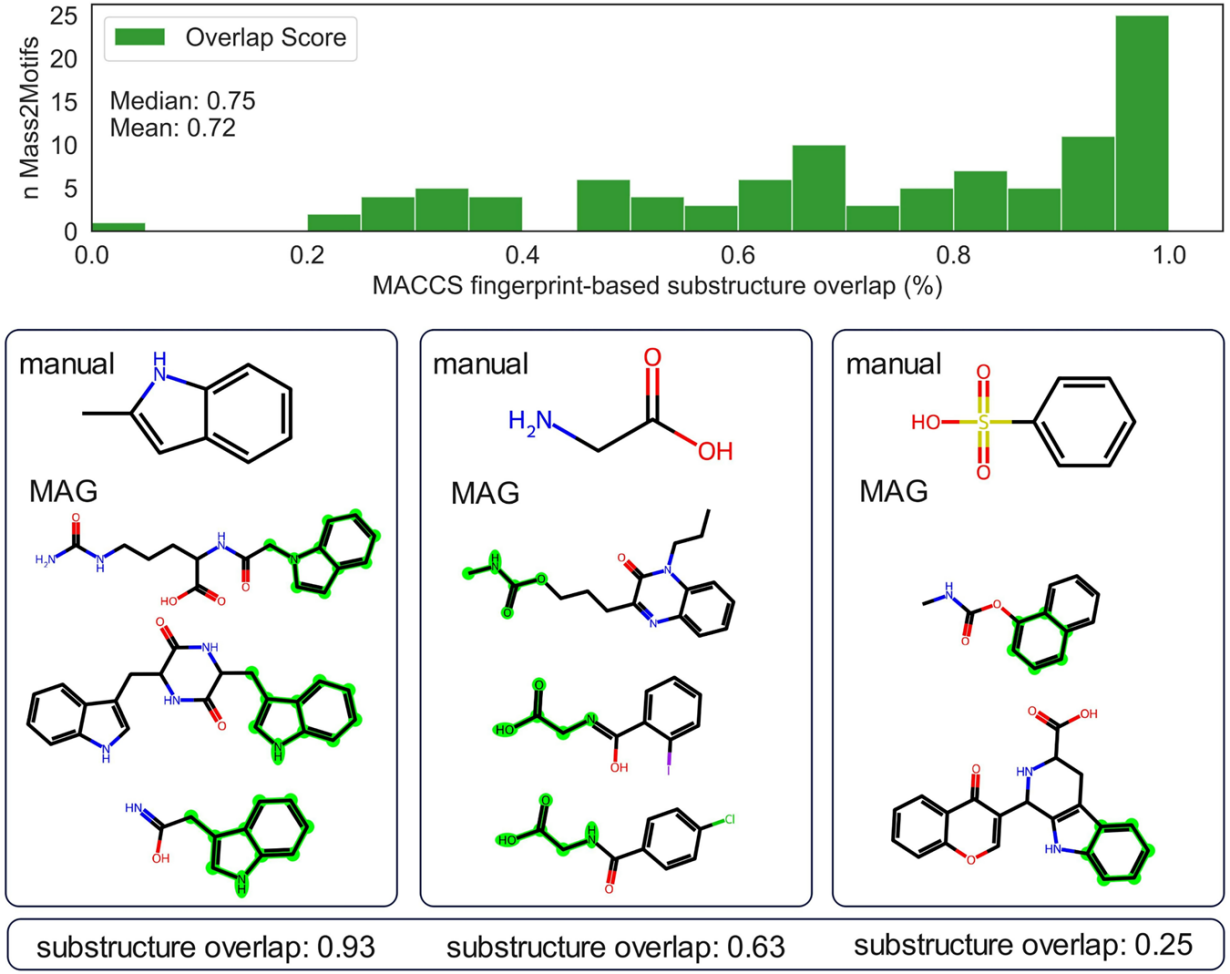
Benchmarking Mass2Motif Annotation Guidance (MAG) on urine-derived Mass2Motifs. Motif Annotation Guidance (MAG) was tested on an experimentally derived urine MotifSet with 101 manually annotated Mass2Motifs. The evaluation was based on MACCS fingerprint overlaps of the MAG and the manual annotated substructures. At the top, a histogram shows the distribution of the alignment between manual and automated structural annotations and at the bottom three examples with high, medium and low substructure overlap scores (from left to right) are shown. The highlighted substructure (green) shows the structural overlap between manual and automated annotations.

In most cases, MAG’s substructure predictions aligned well with manual annotations. For example, in the urine-derived Mass2Motifs MotifSet, MS2LDA 2.0 achieved an average SOS of 0.72 and a median score of 0.75 across 101 Mass2Motifs (Fig. 1). Of these, 20 Mass2Motifs showed a complete substructure match with manual annotations. This means that if users were analyzing these recommendations, they would have very relevant support for 20 out of these 101 Mass2Motifs, and useful support for another 30 Mass2Motifs. Figure 1 shows examples of Mass2Motifs with their respective substructure-overlap scores.

While the urine-derived Mass2Motif MotifSet is based on experimental data, the GNPS and Massbank MotifSets are acquired from spectral library data. For these MotifSets, MAG achieved significantly higher substructure overlap scores, with an average of 0.85 (median 0.93) for the GNPS-based Mass2Motifs and an average score of 0.79 (median 0.93) for the Massbank-based Mass2Motifs (Supplementary. Notes 3.1.4 and Supplementary Fig. S6-S8). The discrepancy between experimental and library-based substructure overlap scores is likely caused by two main reasons. First, MAG makes use of the mass spectral libraries that have an overlap with the two library-based MotifSets, thus containing relevant spectral-structure combinations. Second, the inclusion of more (unrelated) or noisy mass spectra in MS2LDA modeling of the experimental dataset compared to the more cleaned and selected spectra in libraries can lead to noisier Mass2Motifs that make automated structure annotation more difficult.

These results demonstrate that MAG provides reliable automatic guided annotations for positive ionization mode-generated MotifSets, as motifs coming from MotifDB truly represent substructures and can be easily benchmarked against MAG recommendations.

### Modeling and Annotation Performance on Library Spectra and Their Interpretation

A key limitation of the MotifDB benchmark is that MAG only annotated Mass2Motifs that (i) describe a true underlying substructure (manually curated) and (ii) were sufficiently distinct to allow manual annotation. In scenarios where users apply MS2LDA 2.0 to experimental data, many Mass2Motifs will not correspond to any meaningful substructure. Consequently, the ability to distinguish reliable from unreliable Mass2Motif annotations becomes essential.

Because no established benchmark exists to assess unsupervised substructure discovery at a large scale, we used the recently published and well-curated open MS^n^Lib spectral library to simulate a large and diverse mass spectral dataset^6^. Since the MS^n^Lib is composed of reference standards, we can rely on the correctness of compound annotations. For each Mass2Motif, we computed the SOS score (see Methods) between a motif fingerprint and all molecules associated with that motif. The average SOS score reflects how well the MAG annotation captures the true underlying substructure.

**Fig. 2:**
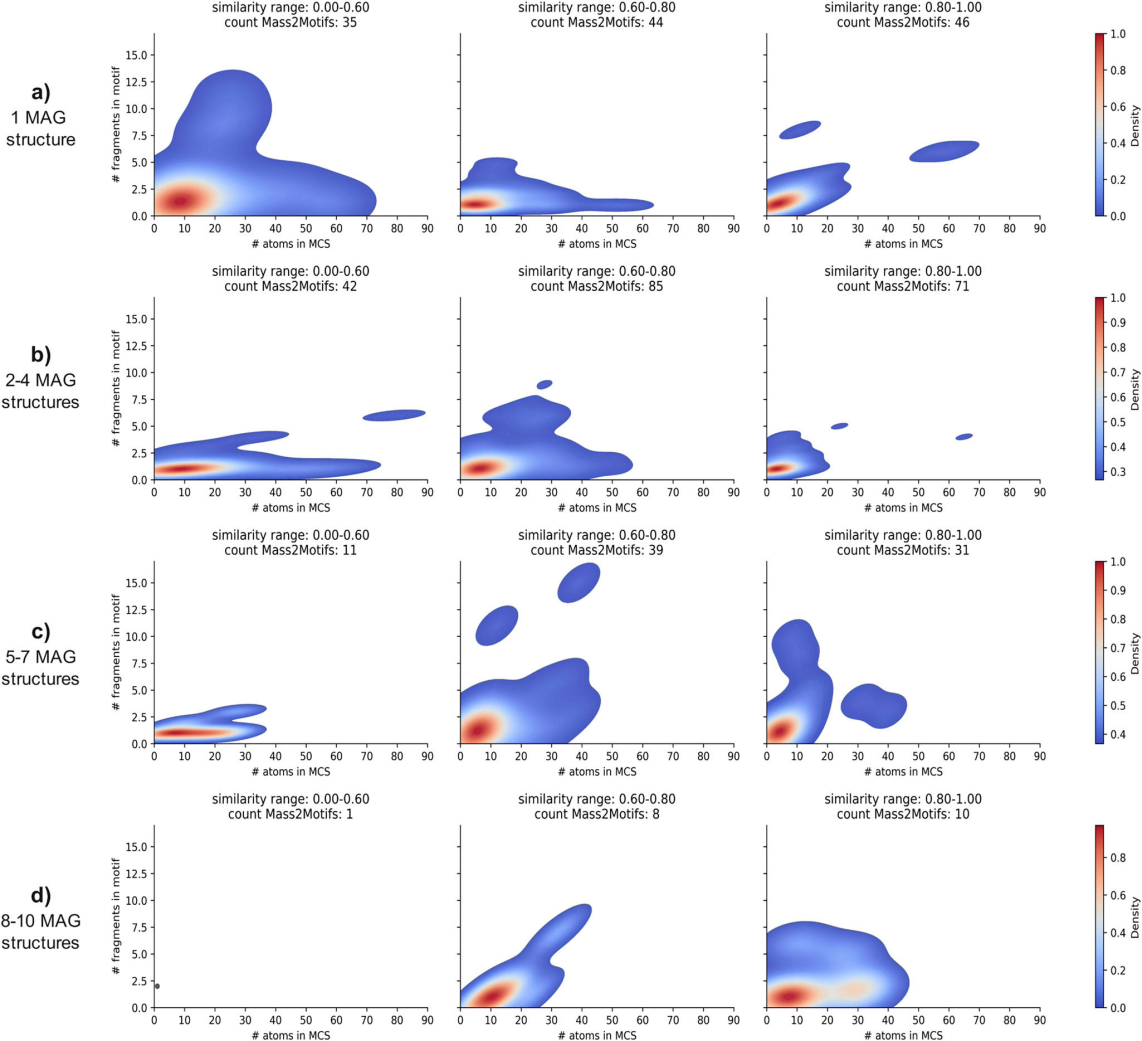
Correlation between Mass2Motif features and annotation performance. All subplots show the distribution of 423 Mass2Motif annotations as a function of the number of Mass2Motif fragment features (y-axis) and the number of MCS atoms (x-axis). The color scale indicates the density of annotations. Distributions are stratified by the SOS score (based on MACCS fingerprints) of the MAG annotation, grouped into three ranges: 0-0.6, 0.6-0.8, 0.8-1.0). **a** One MAG recommendation makes it difficult to distinguish between high and low-quality annotations, because low-quality annotations have an overlapping distribution with high-quality annotations and the amount of low and high-quality annotations is also similar. **b** for 2-4 MAG recommendations the distributions of low and high-quality annotations still overlap, but there are overall more high-quality annotations than low-quality annotations. **c** increasing to 5-7 MAG recommendations allows, for the first time, to distinguish between high and low-quality annotation based on the number of fragments present. Mass2Motifs with more than 5 fragments are only present when the SOS score is above 0.6. Additionally, the number of high-quality annotations is now substantially greater than the number of low-quality annotations. **d** 8-10 MAG recommendations show mostly high-quality annotations.

To emulate how a user might evaluate the quality of a MAG annotation, we stratified results by (i) number of fragments in the optimized Mass2Motif, (ii) size of the maximum common substructure (MCS), and (iii) number of structures recommended by MAG (Fig. 2).

We generated 1,000 Mass2Motifs and during optimization, 432 Mass2Motifs were removed because none of the MAG-proposed annotations shared any fragments with the optimized motif. An additional 145 Mass2Motifs lacked associated molecules when filtered by probability > 0.5. Among the remaining 423 Mass2Motifs, 158 exhibited high-quality annotations (SOS > 0.8), and 176 had intermediate annotation quality (SOS = 0.6–0.8). As also shown in the benchmark above, these intermediate scores can correspond to constitutional isomers (Fig. 1). The remaining 78 Mass2Motifs represent cases where the MAG-proposed annotations are likely incorrect (SOS < 0.6). We note that the here presented numbers are MACCS fingerprint-based and they show a more optimistic result as compared to the more conservative RDKit’s Daylight fingerprint-based results (see Supplementary Notes 3.2 and Fig. S9) with 79 high-quality Mass2Motif annotations and 140 intermediate, well annotated Mass2Motifs.

Analysis of Mass2Motif properties did not reveal a consistent pattern across MAG-proposed annotation quality classes. Most high-quality as well as low-quality annotations typically shared 5–10 atoms in the MCS and contained two shared fragments. Furthermore, the distributions of fragments and losses did not show strong separation between high and poor annotations; even low-quality annotations occasionally contained many fragments or a relatively large, shared substructure. This is especially true when MAG recommended only a single structure. Here, manual inspection remains essential to validate the proposed structure. In contrast, when MAG suggested multiple structures, the likelihood of a reliable annotation increased as the ratio of high-quality and low-quality annotations shifted more towards high-quality annotations. Additionally, for 5-7 MAG-proposed annotations (Fig. 2c) and 8-10 MAG-proposed annotations (Fig. 2d), the low-quality annotations do not exceed five fragment features within one Mass2Motif and allow thereby high- and low-quality annotations to be separated more easily.

In summary, determining whether a MAG annotation captures a distinct substructure based on Mass2Motif properties remains challenging. Mass2Motif features, such as the number of fragments, the size of the shared substructure, and the number of recommended structures, provide helpful tendencies but do not yield strict thresholds. In general, higher values for these parameters increase the probability that a MAG annotation reflects a meaningful substructure, with the number of consistent MAG annotations emerging as the most informative feature. The following case studies will go into more detail about what can be identified and annotated with MS2LDA 2.0. As the case studies are based on experimental data, no ground truth exists, and these case studies therefore have a purely exploratory character.

### Case Study on Pesticides

In our first explorative study, we considered spiked-in pesticides in a complex biological matrix derived from food. Identifying structures in food samples is a challenging process due to their complexity, which stems from the large number of different compounds present^34,35^. To assess whether MS2LDA 2.0 can assist in rapidly identifying Mass2Motifs in a complex sample matrix, we spiked a tomato sample with a standard mixture of 211 pesticides (Supplementary Table S1). This mixture contained various pesticide classes, including organophosphates, carbamates, triazole fungicides, sulfonylureas, and benzoylureas.

The aim of this study was to investigate if MS2LDA 2.0 can successfully model Mass2Motifs of pesticide classes and annotate them correctly in an unsupervised manner. With fifty times more compounds related to the tomato matrix compared to the spiked pesticides, the challenge for MS2LDA 2.0 is to find patterns that are not dominant across the entire dataset.

**Fig. 3:**
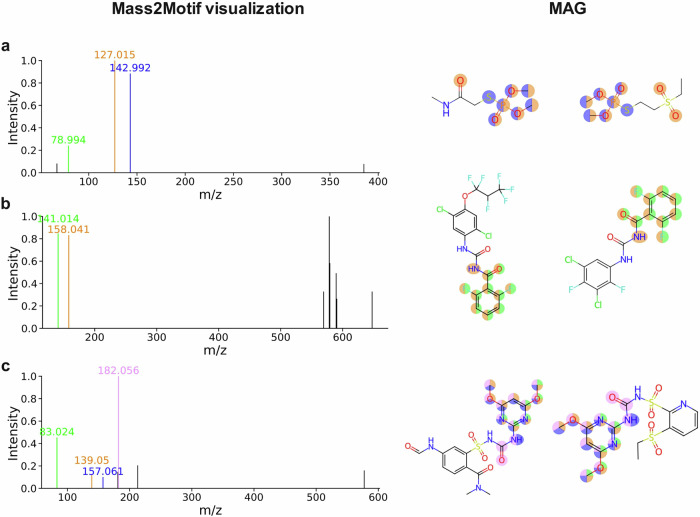
Identified Mass2Motifs related to Spiked-in Pesticides. **a** Mass2Motif 6 shows three fragments related to the MAG of organophosphates. The color of the fragments in the Mass2Motif visualization relates to the highlighted atoms in the MAG and visualizes possible fragmentation pathways of the molecule. **b** Two highlighted fragments from Mass2Motif 90 represent a benzoylurea substructure as shown by MAG annotations. All other fragments above m/z 500 are not directly related to the benzoylurea substructure represented by the Mass2Motif. **c** Mass2Motif 236 shows four common fragments in the Mass2Motif visualization. The MAG suggests a sulfonylurea substructure. While the fragments do not include the sulfonyl group, they are linked to different fragmentation pathways in the 4,6-dimethoxypyrimidine substructure.

MS2LDA 2.0 successfully detected Mass2Motifs that correspond to recurrent substructures of the pesticides present in the spiked mixture (Fig. 3). We focus here on three Mass2Motifs with coherent MAG-proposed annotations that could be linked to distinct pesticide classes: organophosphates, benzoylureas, and sulfonylureas. Two additional pesticide-related Mass2Motifs are presented in Supplementary Notes 3.3.1 and Fig. S10 and S11. To link the spiked compounds to the Mass2Motifs, we used the probability for a compound to be linked to a motif returned by the LDA model, where 0 represents no likely association and 1 a very likely association between the spiked pesticide and a Mass2Motif.

Organophosphates (OPs) are a widely used pesticide class primarily in crop protection^36^ and act as potent cholinesterase inhibitors^37^. Mass2Motif 6 (Fig. 3a) is defined by the mass fragments m/z 127.015, 142.992 and 78.994, representing a dimethylated (mono- or di-thio)-phosphate substructure. This substructure was prevalent in 9 out of 17 spiked OPs: 4 display a strong association with the Mass2Motif (probability > 0.7), two a moderate association (>0.3) and two a weak association (<0.1). The latter two spiked compounds still share two of the three key fragments, indicating that complementary fragment-based queries (e.g., through MassQL) could retrieve them. OPs lacking the dimethylated phosphate group follow alternative fragmentation pathways and are therefore not captured by this Mass2Motif.

Benzoylureas, another major pesticide class, function as insect growth regulators by inhibiting chitin synthases^38^. Mass2Motif 90 (Fig. 2b), characterized by fragments at m/z 158.041 and 141.015, shows a fragmentation pathway typical of benzoylureas, including the loss of an isocyanate group followed by subsequent NH₃ loss^39^. In the spiked mixture, three benzoylureas, along with one oxazole and one isoxazole sharing the typical fragmentation pathways, were present. As we used a complex biological matrix, MS/MS spectra were acquired for three of them. Of these, two benzoylureas showed sufficient association with Mass2Motif 90 (probability > 0.3) to be identified. The third compound did not associate with the Mass2Motif, likely due to the presence of additional unrelated fragments (e.g., m/z 578.41 and 579.41) in the Mass2Motif. As these fragments are only represented in one of the spiked compounds, the generalization of the Mass2Motif decreases. However, we note that, as a part of MAG, Mass2Motif optimization takes place and the optimized fragments of the Mass2Motif could be used instead to improve generalization of the discovered fragmentation pattern.

Sulfonylureas, which interfere with the biosynthesis of specific amino acids in plants^40^, were also identified in the dataset. The core structure of dimethoxylated sulfonylureas was associated with Mass2Motif 236 (Fig. 3c), which contained four key fragments: m/z 83.024, 139.05, 157.061, and 182.056. Overall, 15 compounds belonging to the sulfonylurea class were spiked. Out of the 15 compounds, 6 compounds included a dimethoxylated pyrimidine core of which 5 could be identified with a moderate to high certainty (probability > 0.5). Only one compound showed a low probability of 0.1, but the corresponding spectrum contained all four highlighted fragments, showcasing the specificity of Mass2Motifs. The low probability can be explained by the spiked compound having other more abundant fragments that are not part of the Mass2Motif (further details on the reported pesticides classes and their Mass2Motifs can be found in Supplementary Notes 3.2.2 and Table S2). Again, a MassQL-like search for the identified fragments in the Mass2Motif would be able to identify all spiked compounds with the dimethoxylated sulfonylureas core structure.

Our findings demonstrate that MS2LDA 2.0 effectively identifies substructures related to pesticides of different classes within complex food matrices, thereby further demonstrating the effectiveness of the combination of MS2LDA 2.0 and MAG to discover and structurally annotate spiked-in bioactive scaffolds. We note that the ability of MS2LDA 2.0 to identify Mass2Motifs depends on the frequency and specificity of a fragmentation pattern and the collection of MS/MS spectra of sufficient signal-to-noise. Overall, this case study reinforces its potential application in nontargeted screening of pesticides and their metabolites as well as transformation products.

### Case Study on Fungal Natural Products

The second case study explores the fungal specialized metabolome. Natural product extracts, such as those obtained from edible mushrooms, are complex metabolite mixtures that can contain bioactive compounds with potential therapeutic or toxicological effects. In their study, Khatib et al. (2025) characterized three molecular families in metabolomics profiles of two edible fungal extracts: erinacerins, hericenones, and ergostanes^41^. However, these metabolite families could only be annotated using SNAP-MS^42^ and DEREPLICATOR^43^, as approaches like GNPS library matching or SIRIUS did not provide annotations for these molecules. Here, we assess MS2LDA 2.0’s ability to confirm and extend these annotations through substructure-level analysis.

First, we mapped Mass2Motifs to the previously identified molecular families using MolNetEnhancer^15^. Second, we queried the identified Mass2Motifs in MotifDB using MassQL and conducted Mass2Motif matching against MotifDB. MS2LDA 2.0 inferred the 200 Mass2Motifs in just 1.2 minutes, using an 8 GB RAM machine, rapidly identifying recurring substructural Mass2Motifs across the dataset of 2714 features.

**Fig. 4:**
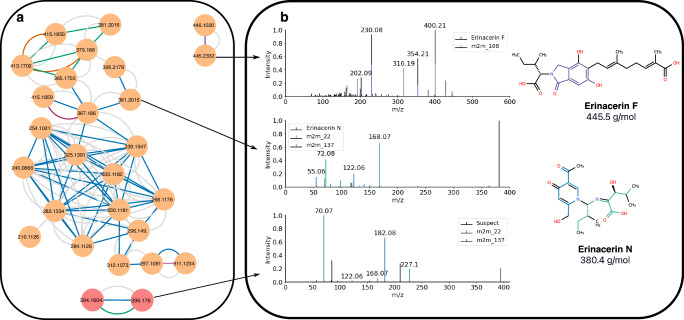
MS2LDA 2.0 results from the fungal natural product case study. **a** Molecular families of erinacerins, the erinacerin N molecular family being the largest (23 orange nodes, top left), erinacerin F (2 orange nodes, top right) and suspect erinacerin molecular family (2 pink nodes, bottom). The data (mass spectra and molecular network) were originally generated by Khatib et al. (2025), while this network figure with shared Mass2Motifs was generated during this study (for further details check Code Availability). The gray edges represent cosine similarity, colored edges represent Mass2Motifs, generated using MolNetEnhancer. **b** Spectra of the molecules manually annotated as erinacerin F and N (top) and the suspect molecule (bottom). Mass2Motifs related fragments colored on top. Structures of the complete erinacerin molecules with the inferred Mass2Motifs by MAG colored (right).

The erinacerin molecular family contains seven Mass2Motifs, with the three most frequently shared ones highlighted in Fig. 4a. Mass2Motif 137 is shared among 19 of 23 nodes in this family, which was identified by MAG as a 4-[4-(2-chlorophenyl)piperazin-1yl]−4-oxobut-2-enoic acid (Fig. 4a, b), containing the substructure-pyridine ring present in erinacerin N (Supplementary Fig. S12). Another frequent motif was Mass2Motif 22, which represents another substructure present in this molecular family identified as 4-methylpentanoic acid (Fig. 4b, Supplementary Fig. S13). Both substructures are characteristic of erinacerin N (C19H28N2O6) related structural features. For Mass2Motif 137, we could find a useful hit as verapamil-related structure in MotifDB via MassQL (Supplementary Table S3). Mass2Motif 22 did not return any results from MotifDB. Another member of the erinacerin family, erinacerin F (C24H31NO7), contains Mass2Motif 108, which corresponds to a 4,6-dihydroxy-2,3-dihydro-1H-isoindol-1-one structure (Fig. 4b). Related indole-like structures were retrieved from MotifDB via MassQL queries, supporting these substructure assignments (Supplementary Fig. S14 and Table S4; further details of the overall molecular family can be found in Supplementary. Notes 3.4.1).

Beyond confirming substructures previously reported as erinacerins, MS2LDA 2.0 uncovered two previously unannotated spectra with precursor m/z of 396.176 and 394.160, respectively, which are not clustered in this molecular family (Fig. 4a, pink nodes). The nodes shared key Mass2Motifs (137 and 22) and their mass differences relative to erinacerin N suggest possible derivatives, e.g., addition of NH (15 Da), with a proposed molecular formula of C_19_H_29_N_3_O_6._ In the suspect molecular family (Fig. 4a, pink nodes), the node of m/z 396.176, contains Mass2Motif 137 and 22 with probabilities of 0.105 and 0.208, and for the precursor of m/z 394.160, the same motifs show 0.451 and 0.38 probabilities, respectively. These findings mean that Mass2Motif 137 and 22 are main topics contributing to the probabilities in both nodes, especially for the node with a m/z of 394.160. These compounds were not annotated by any of the previously used tools, illustrating MS2LDA 2.0’s capacity to complement existing tools in natural product dereplication.

In the hericenone molecular family, MS2LDA 2.0 identified three key Mass2Motifs across 20 nodes (Supplementary Notes 3.4.2 and Fig. S15). Mass2Motif 163 and 186 (present in 15 and 5 nodes, respectively) were annotated both as benzodihydropyran substructures by MAG (Supplementary Notes section 3.4.2 and Fig. S16 and S17), while Mass2Motif 90 (found in 12 nodes) was annotated as a 2-(2-methylbut2-enamido)acetic moiety (Supplementary Fig. S18). Via MassQL querying, we did not obtain any hits for Mass2Motif 163. Mass2Motif 186 retrieved terpenoid-like Mass2Motifs for its annotation (Supplementary Table S5). For Mass2Motif 90, MassQL retrieved terpene, sterone and related structures; a contrasting result compared to its MAG annotation (Supplementary Table S6 and Fig. S18). These results could be explained by the fact that Mass2Motif 90 shows only one top fragment; and when querying against MotifDB via MassQL, there is a high probability of retrieving unrelated molecules sharing the same fragment.

For the ergostane molecular family, MS2LDA 2.0 uncovered six recurring Mass2Motifs (Supplementary Notes 3.4.3 and Fig. S19a). The dominant motifs included Mass2Motif 173, representing a steroid backbone and a carbon side chain, and Mass2Motif 193, both containing a steroid scaffold by MAG recommendation (Supplementary Fig. S20 and S23), which correlates with the molecules manually annotated in the original study (Supplementary. Fig S19b). Mass2Motif 21 is also frequent for this family, annotated as 2,15-dihydroxy-7methyl-6-oxabicyclo[11.3.0]hexadeca-3,11-dien-5-one, which is structurally not very similar to the original structure proposed but they share the five-carbon fused ring (Supplementary Fig. S21). The MassQL results for the Mass2Motifs of the nodes previously reported did not yield additional structural insights for this molecular family (Supplementary Table S7), only Mass2Motif 0 results retrieved a diverse class of motif-related structures such as depsidones, quinones and paraconic acids, which is not aligning with MAG nor the manual annotation (Supplementary Fig. S22 and Table S8).

Finally, we computed the Mass2Motif-matching functionality between our experimental data and the following MotifSets: GNPS, MassBank and LDB MotifDB POS (Supplementary Notes 3.4.4 and Table S9). Several top-ranking, with a threshold over 0.7, supported the substructure proposed by MAG (Supplementary Table S10). For instance, Mass2Motif 10 matched Mass2Motif 13 and 11 with scores of 0.77 from MassBank and GNPS MotifSets respectively, both annotated as alkyl-aromatic substructure indicative for aromatic ring, which correlates with the MAG results shown in Fig. S24, this highlights the value of Mass2Motif similarity screening as complementary strategy for substructure annotation. Another two positive examples with other structural moieties are Mass2Motif 126 and Mass2Motif 61 (Supplementary Fig. S25 and S26).

MAG and Mass2Motif-matching annotations do not align in all the cases; for example, for Mass2Motif 156 (Supplementary Fig. S27), the MAG annotation is 3-propanoyloxy-4(trimethylazaniumyl)butanoate, which does not align with the manual annotation as glycosylation, however, when we inspect the motif (Supplementary Fig. S28), it reveals a loss of 161.1 m/z, as an indicative of a glycosylation. For motifs with high similarity, manual annotated motifs can offer sufficient quality annotations. However, most of the spectra do not show high similarity with the MotifSet manually annotated Mass2Motifs. For instance, a spectral similarity above 0.7 retrieved only 10 results with manually annotated motifs out of the total 2714 spectra. Moreover, all these results are duplicated motifs between GNPS and MassBank. These two MotifSets share many Mass2Motifs, giving us only 5 unique results for this dataset. This reveals the importance of other motif annotation strategies developed in this study, such as the MAG-based and MassQL-based approaches.

### Case Study on Accelerating Biological Discoveries with MAG-empowered MS2LDA

To demonstrate end-to-end utility and verifiability, we reanalyzed the publicly available dataset from Monge-Loría et al. (2025)^18^, who reported the first degradation of amphibactin, a widely abundant siderophore in the ocean, by *Microbulbifer* spp. The original study identified a shared (motif 531) between amphibactin F (m/z 876.529 [M + H]+) and a degradation product (m/z 617.412 [M + H]+), supported by diagnostic fragments: 86, 114, 131, 191, 278 and 450, containing a N-acetyl-N-hydroxy-ornithine substructure. Due to its chelating properties, this annotation was biologically- plausible and supported the interpretation of *Microbulbifer* spp. mediated amphibactin degradation.

Using MS2LDA 2.0 with the preprocessing and parameters reported in the original study, we recovered a corresponding Mass2Motif containing both metabolite features. MAG allowed us to directly link the Mass2Motif to the N-acetyl-N-hydroxy-ornithine substructure as it is present in all its recommended structures. Furthermore, the Mass2Motif optimization resulted in two remaining fragments: 131.08 and 86.06, the same key diagnostic fragments recognized earlier (Supplementary Fig. S29). At the feature level, the original study reported 68 features associated with motif 531 at a motif–spectra probability threshold of >0.1. Our independent reanalysis recovered 32 of these 68 features, indicating substantial concordance despite independent inference and the probabilistic nature of topic modeling (Supplementary Notes 3.5).

We further examined bromotryptamine-related metabolites reported in the same study (bromotryptamine, N-formyl bromotryptamine and N-acetyl bromotryptamine). These features co-occurred in motif_34 and motif_129 (Supplementary Fig. S30 and S31); however, after MAG optimization, each motif was supported by only a single retained fragment, leading to weak and nonspecific substructure recommendations. This potentially reflects limited diagnostic fragmentation information, and possibly incomplete reference coverage in current public libraries. Importantly, both motifs still consistently grouped the brominated features, a relationship not explored further in the original work, and they could be used to mine other datasets for their presence as well.

Together, this case study demonstrates the added value of MAG and its Mass2Motif optimization. At the same time, it highlights cases where MAG is insufficient for reliable automated annotation of motifs; however, we do observe how structurally related molecules are still associated with such motifs. Moreover, MS2LDA 2.0 recovers previously reported biological insights on an external dataset, with MAG substantially accelerating substructure hypothesis generation when diagnostic signals are present.

### Case Study Suspect List

We extended our analysis beyond the MS^n^Lib spectral library by applying MS2LDA 2.0 on 77,218 MS/MS from the positive-mode portion of a recently published suspect spectral list^44^. Each suspect is annotated using the InChI of a known compound plus or minus the mass difference between the known compound and the suspect. Hence, all annotations are based on structural analogs and do not represent full structural annotations. Given its size and diversity, this suspect library is an ideal test case for evaluating MS2LDA 2.0’s large-scale analysis capabilities on a set of heterogenous mass spectra of various structures generated on different instruments and with various collision energies by different contributors, all deposited to public GNPS libraries.

Out of 1,500 generated Mass2Motifs, we investigated the MAG annotations manually and compared them with the annotation of the suspect (further details can be found at the Supplementary Notes 3.6). Some examples where MS2LDA 2.0 shows a positive alignment with the proposed suspect can be found in the S.I. (Supplementary Fig. S32). To further verify the structural annotations, we used the mass spectral prediction tool CFM-ID 4.0^45^ (details Supplementary Notes 3.6.1-3.6.4 and Tables S11-S13) to assess agreement between the suspect and the Mass2Motifs. To highlight the presence of the Mass2Motif across the suspect library, we retrieved the number of compounds for which this Mass2Motif had the highest association based on LDA calculated probabilities.

In this explorative case study, we highlight some manually chosen suspects, representing a diverse class of compounds: peptides (Supplementary Fig. S33), steroids (Supplementary Fig. S34), sulfonamides (Supplementary Fig. S35 and S36), and glycosides (Supplementary Fig. S37). Except for the sulfonamide suspect, the other three suspects are explained by two Mass2Motifs. This shows another characteristic of Mass2Motifs: they can act as building blocks for molecules and complement each other. In theory, this would allow annotation of known compounds based on their known building blocks. However, this characteristic also reveals a limitation: Mass2Motifs rely on similar fragmentation pathways, which may not always occur even for identical substructures across different molecules. Finding building blocks is also expected to be more common when many analogous compounds are present as they are in the suspect list.

Overall, MS2LDA 2.0 can be run on large datasets and extract meaningful Mass2Motifs, which identify building blocks of molecules. The manual selection of Mass2Motifs that represent relevant substructure patterns emphasizes that the extraction of meaningful Mass2Motifs is not automated and is still a bottleneck in the current version. However, workflows like MAG can support this selection to some extent (Fig. 2).

### Integration with other computational mass spectrometry frameworks and computationally efficient topic modeling

**Fig. 5:**
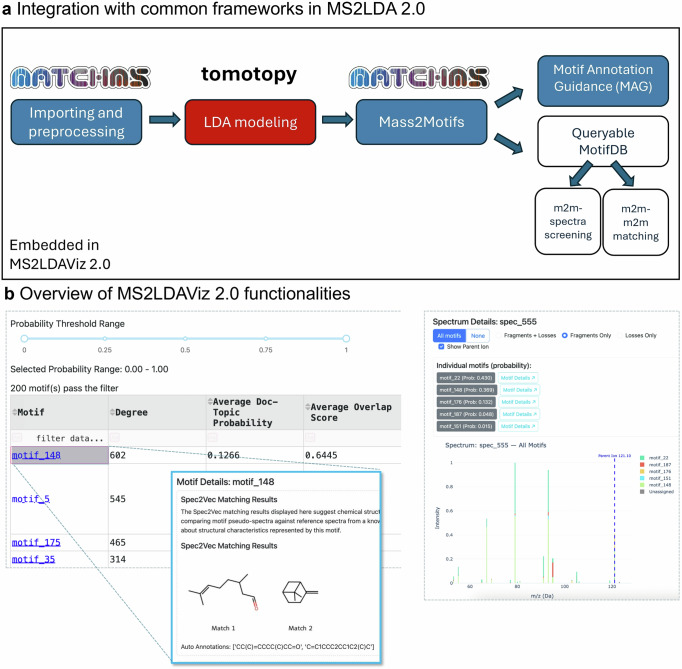
Workflow and Tool Integration in MS2LDA 2.0. **a** Integration of MS2LDA 2.0 with common computational mass spectrometry frameworks. Mass spectrum importing and preprocessing are performed using matchms, while topic modeling is handled by the optimized LDA implementation provided by Tomotopy. The resulting Mass2Motif objects are now fully interoperable with multiple downstream tools, allowing seamless integration with automated Motif Annotation Guidance (MAG) and motif querying of MotifDB via MassQL. MotifDB can also be used for Mass2Motif-Mass2Motif (m2m-m2m) matching and Mass2Motif-spectra (m2m-spectra) screening. All these functionalities are now incorporated in an interactive MS2LDAViz 2.0 application. **b** Overview of the MS2LDAViz 2.0 application functionalities. The left panel shows a ranked table of inferred Mass2Motifs along with automated structural annotations provided by MAG. Users can interactively inspect detailed annotations, including suggested chemical structures, by selecting individual motifs. The right panel illustrates how a single MS/MS spectrum can be decomposed into multiple contributing Mass2Motifs, a unique feature of the MS2LDA 2.0 approach that facilitates rapid interpretation of complex spectra.

In the re-design of MS2LDA 2.0, seamless integration with widely used Python-based frameworks for computational mass spectrometry was another main goal (Fig. 5a). The original version relied on custom Python code to process mass spectra and handle Mass2Motifs, limiting interoperability with community-standard tools. Instead, MS2LDA 2.0 has adopted standardized frameworks such as matchms^30^ to process mass spectra, including filtering, similarity calculations and data import/export, through well-maintained, modular components. This improves reproducibility, maintainability, and compatibility with other tools, especially those also built on the matchms framework.

MS2LDA 2.0 now utilizes Tomotopy for LDA inference^46^, a library written in C + + that supports parallelized inference using collapsed Gibbs sampling. This shift enables substantial speed improvements for the topic modeling: on an AMD EPYC 7532 32-Core Processor using 4 cores, MS2LDA 2.0 was 3x to 14x as fast as the previous version, which used a single-threaded batch variational Bayes implementation in NumPy. For example, discovering 400 Mass2Motifs in the GNPS Suspect-List library (87,899 spectra in total) required 3.46 hours with MS2LDA 2.0 on this hardware setup, compared to 20.51 hours with its predecessor (Supplementary Notes 3.7 and Fig. S38). When reducing the number of cores and using a single core as the original MS2LDA version, the two versions showed similar speed (Supplementary Fig. S39). This shows the importance of parallelized computing inference by Tomotopy, making large-scale analysis now feasible.

After MS2LDA 2.0 topic modeling, the resulting Mass2Motifs are now well integrated with matchms, allowing integration with specialized downstream tasks. First, MAG utilizes Mass2Motifs as input for Spec2Vec^32^ to aid in the structural annotation of substructures. Based on the resulting annotations, structural fingerprints such as MACCS or Daylight can be generated, improving the annotation capabilities that were possible with previously available workflows. Second, Mass2Motifs generated by MS2LDA 2.0 are now stored in an up-to-date standard file format based on the MassQL framework^33^ (for further details check Supplementary Methods 4.1.1 and Table S14) This enables flexible querying and retrieval of Mass2Motifs based on characteristic fragments and losses within MotifSets. Furthermore, the current setup allows researchers to easily contribute their own MotifSets generated with MS2LDA 2.0 to MotifDB, with the goal of making it an expanding community-driven motif annotation effort.

The newly developed MS2LDAViz 2.0 app makes visualization of several aspects of Mass2Motifs easy and interactive (Fig. 5b and Supplementary Fig. S43-S50). Previously, visualization was implemented using a Django-based web application, which requires considerable web development expertise, making it challenging for researchers without extensive software development backgrounds to install, modify, or extend upon. In contrast, the recently developed app is written in Dash, which offers a simpler Python-based approach. This makes it possible for data scientists and researchers to more easily visualize annotation output and to customize and extend the views themselves (Fig. 5b). We refer to section 4.3.1 of the Supplementary Methods for more details on MS2LDAViz 2.0.

As a demonstration of seamless integration into existing analysis environments, we visualized molecular networks in Cytoscape using the MolNetEnhancer concept^15^ in the Case Study Natural Products. This enabled us to confirm the structural annotation of previously annotated metabolites as well as to annotate unknown fungal metabolites. Our analysis was further supported by querying already annotated MotifDB-Mass2Motifs to annotate several substructure patterns.

## Discussion

Untargeted metabolomics studies continue to face major challenges in structural elucidation of metabolite features, and full structure identification is often not feasible. Substructure-level analysis offers a practical route to chemical insights. The original MS2LDA introduced topic modeling for fragmentation patterns (Mass2Motifs) from MS/MS data. While influential, it was limited by its slow computation, dependence on manual annotation, and outdated implementation rendering it incompatible with other frameworks. MS2LDA 2.0 addresses these limitations by providing a modern scalable framework and by introducing a key feature, the automated Motif Annotation Guidance (MAG).

Across manually curated motifs from MotifDB, MAG retrieved correct substructure annotations with high overlap (mean score >0.75), demonstrating that automated annotation can achieve expert-level performance on validated motifs. When applied to the large MS^n^Lib spectral library^6^, MS2LDA 2.0 scaled efficiently and produced a subset of Mass2Motifs (7%-15%) with high-confidence MAG annotations. Together, these two benchmarks show that MAG can reach expert-level performance on curated MotifDB motifs and, in large heterogeneous datasets, yields a characteristic fraction of Mass2Motifs with high-confidence annotations and many more with intermediate-quality guidance, which is still useful for hypothesis generation. Mass2Motifs containing a higher number of fragments and multiple consistent recommended structures tend to yield the most reliable annotations. However, qualitative assessment of annotation quality, based on fragment count, common substructure size or number of recommended structures remains challenging and requires further refinement of evaluation metrics. Furthermore, MAG-proposed annotations are biased towards the availability of mass spectra-structure pairs in mass spectral libraries; fortunately, over the last years, open spectral libraries have been growing both in size and structural diversity, with MS^n^Lib as a prime recent contributor to this trend.

In experimental datasets, MAG successfully identified pesticide-related motifs in spiked tomato samples and detected many of the added pesticides. For fungal extracts, MS2LDA recovered known substructures within three molecular families and provided additional hypotheses for unconnected nodes, showing its application to complex natural product mixtures. For the siderophore case study, Mass2Motif reproducibility was achieved compared to the original study and MAG-capabilities were also highlighted. As expected, the performance varied with the coverage of spectral libraries, and motifs representing unknown chemistry still depend on expert interpretation.

To complement MAG, MS2LDA 2.0 offers post hoc annotation strategies through integration with external tools. Since Mass2Motifs can now be treated as pseudo-spectra, users can compare them directly to MotifSets in MotifDB using spectral similarity matching, providing targeted-sample support for structural hypotheses. This approach proved useful for fungal motifs such as those associated with lysine and phenylalanine. In parallel, MassQL provides a complementary route by enabling precise, query-based searches of fragments and losses present in Mass2Motifs against curated motif repositories. Using the fungal dataset, MassQL-derived annotations were consistent with MAG suggestions, highlighting the benefit of combining automated recommendations with query-driven pattern matching.

All current functionalities that include MAG annotation, MotifDB matching, MassQL querying and visualization of Mass2Motifs in molecular networks are unified in the MS2LDAViz 2.0 application, making MS2LDA 2.0 more accessible to non-expert users. Besides, the MS2LDAViz 2.0 app still holds the original key plots for manual inspection of Mass2Motifs (especially useful for motifs that do not have reliable MAG annotations) and now allows to interactively adapt key threshold settings like the probability and overlap thresholds that associate spectra to Mass2Motifs.

Despite these advances, some limitations remain. As in the original implementation, users must specify the number of Mass2Motifs prior to inference, and results can vary with hyperparameter choices (see recommendations in the Supplementary Discussion 5.1). While repeated runs or varying topic numbers can help to assess stability, nonparametric Bayesian approaches, such as Hierarchical Dirichlet Process (HDP)^47^ may offer long-term solutions but they are constrained by computational speed. MAG currently supports only positive-mode data, reflecting the availability of these reference spectra; the ongoing expansion of negative-mode libraries will enable broader applicability. Finally, Mass2Motifs representing truly novel chemistry still require detailed manual interpretation. Integration with MotifDB and MassQL will ease dissemination and large screening of newly annotated motifs.

In summary, MS2LDA 2.0 combines scalable motif discovery with semi-automated annotation and is integrated into the broader metabolomics ecosystem. Its interoperability with community driven resources such as matchms and MassQL provides a practical foundation for substructure discovery in large MS/MS datasets. As mass spectral libraries and Mass2Motif repositories continue to grow, we anticipate that Mass2Motif annotation will become an increasingly standard component of untargeted metabolomics workflows.

## Methods

We note that for all case studies and benchmarks, MS2LDA 2.0 parameters need to be selected. Additionally, when running MS2LDA 2.0 a convergence curve of the LDA modeling process is generated to assess if the model was run for sufficient time. All selected parameters, their descriptions and recommended ranges, and generated convergence curves can be found in Supplementary Methods 4.1.2-4.1.3 and Supplementary Fig. S40-S42 and Table S15. All data used in this study can be found in the Data Availability section.

### MotifSets for MAG Benchmarking

The selection of suitable MotifSets for the Motif Annotation Guidance benchmarking was based on the number of annotated Mass2Motifs and their annotation confidence. We selected three MotifSets from MotifDB: urine-derived Mass2Motifs^18^ (101 Mass2Motifs), GNPS library-derived Mass2Motifs^23^ (73 Mass2Motifs) and MassBank library-derived Mass2Motifs^23^ (42 Mass2Motifs). These three MotifSets were chosen as they represent relatively large Mass2Motif collections whose annotations were reviewed with confidence. To convert the initial descriptive manner of Mass2Motif annotations into computer readable SMILES, every description was manually inspected, and the SMILES for the described compound corresponding to the annotated substructure was retrieved from PubChem^48^.

### Case Study on Pesticides

We experimentally prepared a tomato sample spiked with 211 pesticides and performed a HPLC-HRMS/MS analysis. The names of all spiked compounds can be found in a separate file in the Supplementary Table S1.

### Sample preparation Case Study on Pesticides

First, 10 grams of blank tomato sample were transferred into a 50 ml Greiner tube. 10 ml of acetonitrile/1% formic acid were added and extracted in an overhead shaker for 30 min. Then, 4 grams of MgSO_4_ and 1 gram sodium acetate were added, and the mixture was vortexed for 1 min to induce phase separation. Afterwards, the sample was centrifuged for 5 minutes at 2671 x g. 250 µl of the upper layer of the extract was transferred into an LC vial, 25 µl of a 1000 ng/ml pesticide mixture containing 211 compounds and 225 µl of water were added. After mixing using a vortex, this vial was used for the LC-HRMS/MS analysis.

### LC and MS settings Case Study on Pesticides

Liquid chromatography was carried out on a Vanquish UHPLC system (Thermo Fisher Scientific). The LC eluents A and B were water and methanol both containing 2 mM ammonium formate and 0.1% formic acid. The gradient started with 100% phase A with a linear increase of phase B, which reached 100% at 15 min. For 6 min the eluent stayed at 100% of phase B and then increased to 100% phase A with a linear gradient within 1 min. The run stopped after 25 min. The flow rate was set to 0.3 mL/min and the injection volume was set to 5 µL. The column was a Waters UPLC BEH C18 1.7 um 2.1 mm × 100 mm with a column oven temperature of 50 °C.

Mass spectrometric detection was carried out on a Thermo IQX Orbitrap instrument (Thermo Fisher Scientific) operated in positive ESI mode. Mass spectrometry was performed with a sheath gas flow rate of 48, auxiliary gas flow rate 11, and sweep gas flow rate 2. The spray voltage was set to 3400 V while the ion transfer tube temperature and the vaporizer temperature were set to 320 °C and 350 °C, respectively.

For MS1 the resolving power (R) of 120 K was used, with a maximum injection time of 246 ms, an automated gain control (AGC) target of 400,000, and RF lens of 70%. All data were acquired in centroid mode from a mass range from 100 to 1000 m/z. A dynamic exclusion filter was applied where compounds were excluded for 3 s after the first appearance with a 10-ppm mass tolerance, also excluding isotopes.

An inclusion and exclusion list were used (see all compounds in Supplementary Table S1). The inclusion list contained 36 compounds with neurotoxic properties (out of the 211 spiked compounds), and the exclusion list was automatically created based on the procedure blank run.

MS/MS data were acquired using the HCD cell for fragmentation with a normalized and stepped collision energy of 30/80%. The maximum injection time was 22 ms with an AGC target of 50000 and the Orbitrap resolution was set to 15 K.

### Case Study on Fungal Natural Products

The natural product dataset is publicly available^49^ and contains HPLC-MS/MS data measured in positive mode and data dependent acquisition mode for two fungal species: the mushrooms Hericium erinaceus and Pleurotus eryngii. We applied MS2LDA 2.0 on the dataset, thereby focusing on finding the correlations between Mass2Motfis and structures reported in the paper of three biochemically relevant metabolite groups that could not be annotated with GNPS library matching or SIRIUS^9^ in-silico annotation.

### Case Study using the Suspect List

The suspect list comprises a large collection of 87,899 mass spectra derived from published datasets in the form of suspects that are related to known library compounds^44^. The complete dataset was used to showcase MS2LDA 2.0 computational efficiency. As input for the case study, the 77,218 suspects measured in positive ionization mode were used to evaluate its automated annotation capabilities.

### Datasets used for Scalability Evaluation

To test the computational efficiency of MS2LDA 2.0, we downloaded the following libraries from the GNPS public libraries^4^: GNPS-NIH-NATURALPRODUCTSLIBRARY (1,267 spectra), GNPS-NIST14-MATCHES (5,185 spectra), GNPS-NIH-NATURALPRODUCTSLIBRARY_ROUND2_POSITIVE (7,377 spectra), BERKELEY-LAB (18,352 spectra), and the Suspect Library (87,899 spectra). All libraries were downloaded from the GNPS webpage. We calculated the time that the LDA inference took for the original MS2LDA implementation and version 2.0, thereby varying the number of discovered Mass2Motifs (100, 200, 300, 400). We used 1000 Gibbs sampling steps for MS2LDA 2.0 and 100 variational iterations for the original MS2LDA for the number of steps. We note that 100 iterations for the initial version do not always lead to completely converged models, especially for larger datasets.

### Retraining Spec2Vec and Reference Database

A dataset combining publicly available positive ionization mode mass spectra from MoNA, MassBank, GNPS and Brungs et. al. library containing 502,685 spectra (dated 15/02/2025) was downloaded, pre-processed, and used to retrain Spec2Vec (details at https://github.com/vdhooftcompmet/MS2LDA/tree/main/notebooks/Spec2Vec), and it served as the reference library for the Motif Annotation Guidance. The mass spectral library dataset is publicly available from Zenodo (https://zenodo.org/records/12543129).

### Computational Methods

#### MS2LDA 2.0 Framework and Implementation

MS2LDA 2.0 is a framework designed to identify substructures in tandem mass spectrometry (MS/MS) datasets. The workflow consists of five main steps: (a) spectrum loading and preprocessing, (b) topic modeling using Latent Dirichlet Allocation (LDA), (c) Mass2Motif annotation, (d) visualization, and (e) Mass2Motif storage and querying via MotifDB. MS2LDA 2.0 supports standard input formats such as.mgf,.mzML, and.msp. Mass spectrum processing, including ion peak filtering and metadata handling, is performed using the matchms library (v0.27.0). Mass fragment peaks and neutral losses are rounded to two or three significant digits (default: two) and converted to string representations (e.g., frag@70.04) to use for topic modeling. Peak intensities are discretized by scaling normalized intensity values (0-1) to integers (0-100), with each fragment or loss represented as repeated words proportional to intensity. This ensures that more intense peaks have greater influence in topic modeling, analogous to frequent words in text-based LDA.

LDA is performed using the tomotopy library^46^ (v0.13), producing Mass2Motifs as distributions over fragment and loss features. These features are then converted back to their original numerical values (e.g., frag@70.04 → 70.04), with topic-word probabilities (β values) used as normalized intensities in the final Mass2Motif representations. For further details on the underlying LDA method and its application to metabolomics data, we refer to the original MS2LDA paper^18^.

A web-based application, MS2LDAViz 2.0, has been developed using the Dash framework (v2.18). This app allows users to interactively explore Mass2Motif spectra, annotations, and Mass2Motif comparisons. Additionally, a command-line interface and Jupyter notebooks are provided for users who prefer scripted analyses or customizable interactive data exploration.

Mass2Motifs can be stored and queried using the MassQL library (v0.0.15). Queries are expressed using the MassQueryLanguage4Mass2Motifs syntax, an extension of MassQL specifically designed for searching Mass2Motifs (details in Supplementary Methods 4.2 and Table S16). These queries allow users to search against MotifDB, a community-driven repository of previously annotated MotifSets (see MotifDB Update and Integration), allowing efficient retrieval and comparison of Mass2Motifs across different datasets.

### Convergence Criteria

In the original MS2LDA, users were required to manually set the number of iterations for training the LDA model. In the updated version, MS2LDA 2.0 introduces early stopping, allowing the training to terminate automatically based on user-set convergence criteria. Users can specify a convergence threshold evaluated against one of four available metrics: perplexity, maximum likelihood, document-topic entropy, or document-word entropy. At defined intervals (default step size = 50 iterations), the relative change in the selected metric is calculated over recent checkpoints (default window size = 500 iterations). If this relative change falls below the specified threshold, training stops early. Otherwise, training continues until the maximum number of iterations is reached. Examples of convergence plots illustrating this process are provided in the Supplementary Methods 4.1.3.

### Motif Annotation Guidance (MAG)

A detailed example of how MAG identifies structures for annotation guidance can be found in the Supplementary Notes 3.1.1 and3.1.2. Here, we provide a brief overview of its main innovations leading to the automated MAG. First, Spec2Vec^32^ is used to compute embeddings for each Mass2Motif as well as the embeddings for compounds in the reference database. Then, cosine similarity between embeddings is efficiently calculated using the FAISS library^50^. By default, the top 10 database hits are retrieved as candidate matches. However, depending on the database content, fewer relevant hits may be returned.

To filter out irrelevant database hits that may represent different substructures, we apply a masking strategy. Masking involves systematically removing one fragment or loss at a time from the Mass2Motif and recalculating its embedding using Spec2Vec. For example, given a Mass2Motif containing fragments 70.04, 91.03, and 125.69, each fragment is masked in turn, resulting in three modified motifs: one without 70.04, one without 91.03, and one without 125.69. An embedding is generated for each masked variant, and its cosine similarity is recalculated against the initially retrieved database hits.

The idea behind masking is that database hits corresponding to the same substructure will exhibit a similar change in similarity scores when key fragments or losses are masked. For instance, if four of the five hits share a substructure associated with fragments 70.04 and 125.69, their similarity scores are expected to decrease when those fragments are masked. In contrast, a fifth hit representing a different substructure (primarily associated with fragment 91.03) will be affected differently.

This behavior is identified by applying agglomerative clustering (scikit-learn version 1.5.1) to the resulting cosine similarity matrix from all masked variants. Ideally, clustering isolates the hits that represent the same substructure. The cluster that includes the best original database hit is then selected to guide MAG.

### Mass2Motif Fingerprints

MS2LDA 2.0 creates fingerprints to represent possible substructures in molecules. These fingerprints are based on results from the Motif Annotation Guidance (MAG). MAG works by identifying a set of candidate chemical structures that are likely to share a common substructure and belong to the same Mass2Motif.

To build a fingerprint that captures just this shared substructure, MS2LDA 2.0 first generates a fingerprint for each candidate structure. Then, it compares them and keeps only the bits (chemical features) that appear in at least 80% of the fingerprints (default threshold). This way, the final fingerprint highlights the features most likely to belong to the shared substructure (for further details Supplementary Methods 3.1.1).

### MotifDB Update and Integration

The updated version of MotifDB enables storing, querying, and retrieving annotated Mass2Motifs. MotifDB stores Mass2Motifs in an extended MassQL format (version 0.15.0), adapted to handle differences between spectra and Mass2Motifs. One substantial difference is the explicit storage of losses, as these cannot be derived directly from the matchms spectrum object for Mass2Motifs due to the absence of precursor ions. Additionally, since Mass2Motifs lacks MS1-level data and retention times, certain MassQL functionalities, such as MS1-level or retention-time-based queries, are not supported in MotifDB. Additionally, we extended the current format by supporting the storage of additional metadata which can be found in Supplementary Methods 4.1.1. This additional metadata was inspired by the original MotifDB and was found helpful in selecting relevant MotifSets to match Mass2Motifs, for example based on shared origin. The metadata can also be queried using the new METAFILTER:metadata newMotifDB-compatible format. Furthermore, the current MotifDB is stored within the MS2LDA 2.0 GitHub repository (https://github.com/vdhooftcompmet/MS2LDA/tree/main/MS2LDA/MotifDB), making it easier to version and trace changes over time, and it is downloaded directly when installing MS2LDA 2.0.

### Visualization

A web-based application has been developed to allow users to visualize discovered Mass2Motifs and inspect screening results. This MS2LDAViz 2.0 application replaces the previously deployed ms2lda.org^22^, which is no longer actively maintained due to computational efficiency issues and its customized code structure. By contrast, the current visualization leverages Dash^51^, offering a simpler development syntax and a more straightforward deployment on remote servers or local machines. Compared to Django, Dash has a lower barrier to entry and is more widely familiar to data scientists, making it easier for the metabolomics community to contribute and expand visualization features. We also introduce a new way to visualize Mass2Motifs with its fragments and losses with the optimized fragments highlighted (Supplementary Fig. S5).

While the legacy ms2lda.org website is hosting a demo version of the app to visualize MS2LDA 2.0 models, users now also have the option to install MS2LDAViz 2.0 locally, increasing accessibility and flexibility across different research scenarios. A high-level overview of the functionalities in the application is shown in Fig. 5b with a more detailed overview being available in Supplementary Methods 4.3.1 − 4.3.6. Ultimately, we envision MS2LDAViz 2.0 as a community-driven resource enabling users to browse, annotate, and contribute annotated Mass2Motifs back to MotifDB, fostering collaboration in substructure discovery.

### Mass2Motif-Mass2Motif Matching

One functionality from the initial version that is no longer available in MS2LDA 2.0 is the use of a MotifDB-MotifSet as fixed topics made possible by custom LDA inference code. Previously, fixed topics allowed predefined Mass2Motifs to be directly identified within new datasets by combining the discovery of free (unsupervised) and fixed (supervised) topics, the latter with manually curated structural annotations, allowing for the knowledge from previously annotated analyses to be transferred to a future analysis. This functionality is omitted in the current release due to a limitation in Tomotopy, which does not support exact fixing of previously discovered topics. As an alternative, we allow users to screen for previously characterized motifs by computing cosine similarities between Spec2Vec embeddings of newly generated Mass2Motifs and existing MotifSets. These MotifSets may originate from prior experiments or from MotifDB. This screening approach differs from the Motif Annotation Guidance (MAG) described above, and is complementary, as its primary purpose is to rediscover previously annotated motifs through Spec2Vec matching, without explicitly verifying the importance of individual fragments (Supplementary Methods 4.3.6)

### Reporting summary

Further information on research design is available in the Nature Portfolio Reporting Summary linked to this article.

## Supplementary information

Supplementary Information

Reporting Summary

Transparent Peer Review file

## Source data

Source Data

## Data Availability

For retraining the positive mode Spec2Vec model we used a dataset combining GNPS, MassBank and the MS^n^Library and the dataset can be downloaded from Zenodo [10.5281/zenodo.12543129]. The used MSnLibrary in positive mode is freely available and has been downloaded and filtered to contain only MS^n^-derived library and positive ionization mode [10.5281/zenodo.16882111]. The pesticide dataset is available from GNPS-MassIVE (MSV000097937) [10.25345/C5RJ49707, https://massive.ucsd.edu/ProteoSAFe/dataset.jsp?task=a8b81cf6d0c84f6bb4c533244af6b2a9]. The experimental fungal dataset used in this study is publicly available and was downloaded from MassIVE (MSV000090919) [10.25345/C5W669D5F, https://massive.ucsd.edu/ProteoSAFe/dataset.jsp?task=19bf1c83f967435b8de532ac0d617ae5]. For Fig. 4, the molecular network used was from the original link reported by Khatib et al. (2025) [https://gnps.ucsd.edu/ProteoSAFe/status.jsp?task=069e4c5dc7d846a4a96e485091d199a4], and the table generated with features and their associated Mass2Motif (see Code Availability) was imported on top of this network using Cytoscape (3.11). The data for the siderophore case study is available from GNPS-MassIVE (MSV000096127) [10.25345/C5CF9JJ8G, https://massive.ucsd.edu/ProteoSAFe/dataset.jsp?task=7fd472fa319a426aa4a4910902d0ae13]. The data for the case study analyzing the suspect library can be downloaded from Zenodo [10.5281/zenodo.8282733]. Results including the Mass2Motifs and their annotation (including Mass2Motifs for the benchmarking of MAG) from all the datasets analyzed in this manuscript are available from Zenodo [10.5281/zenodo.20179680], and as notebooks in the GitHub repository [https://github.com/vdhooftcompmet/MS2LDA/tree/main/notebooks/Paper_results]. Source data are provided with this paper.

## Code availability

The MS2LDA 2.0 framework, including all modules and scripts for reproducing the results presented in this paper, is available at https://github.com/vdhooftcompmet/MS2LDA and archived on Zenodo (DOI: [10.5281/zenodo.16881225]) under the MIT License. The software can be easily installed via PyPI using pip install ms2lda. Files for retraining Spec2Vec models are archived and versioned on Zenodo (https://zenodo.org/records/12543129). The generation of a table with features and associated Mass2Motifs for network visualization (as used in the experimental fungal dataset) can be found in https://github.com/vdhooftcompmet/MS2LDA/blob/main/notebooks/Paper_results/Notebook_retrieve_spec_motifs.ipynb. Comprehensive installation instructions for Linux, MacOS, and Windows, and user guides for MS2LDA 2.0 and its visualization interface MS2LDAViz 2.0 are provided in the documentation (https://vdhooftcompmet.github.io/MS2LDA/). Python versions and dependencies can be found on GitHub (https://github.com/vdhooftcompmet/MS2LDA/blob/main/MS2LDA_environment.yml). For data preprocessing we used MZmine (4.3.3) and for network visualizations we used Cytoscape (3.11).
