# Supplementary material for: Large-scale discovery and annotation of substructure patterns in mass spectrometry profiles
Source: Nat Commun. 2026 Jul 6;17:8350. doi: 10.1038/s41467-026-75038-0 (PMC13473578; doi:10.1038/s41467-026-75038-0)
Supplement: Supplementary file 1 [file 41467_2026_75038_MOESM1_ESM.pdf]

Supplementary Information with:

Large-scale discovery and annotation of substructure patterns in mass spectrometry profiles

Laura Rosina Torres Ortega[1,#,*], Jonas Dietrich[1,2,#,*], Joe Wandy[3], Hans Mol[2] & Justin J. J. van der Hooft[1,4*]

[1]Bioinformatics Group, Wageningen University & Research, Wageningen, the Netherlands

[2]Wageningen Food Safety Research – part of Wageningen University & Research, Wageningen, the Netherlands

[3]College of Medical, Veterinary & Life Sciences, University of Glasgow, Glasgow, United Kingdom

[4]Department of Biochemistry, University of Johannesburg, Johannesburg 2006, South Africa

[#]These authors contributed equally

[*]Corresponding Authors: LRTO, rosina.torresortega@wur.nl; JD, jonas.dietrich@wur.nl; justin.vanderhooft@wur.nl, JJJvdH.

Table of Contents

1. Supplementary Figures ***3***

2. Supplementary Tables ***53***

3. Supplementary Notes ***64***

3.1 Automated Mass2Motif Annotation Guidance and Benchmarking **64**
3.1.1 The three steps of Mass2Motif Annotation Guidance 64
3.1.2 Mass2Motif Optimization 65
3.1.3 Substructure Overlap Score Definition 65
3.1.4 Extended Benchmarks 66

3.2 Correlation between Mass2Motif features and annotation performance **66**

3.3 Case Study Pesticides **67**
3.3.1 Additional Mass2Motifs related to Pesticides 67
3.3.2 LDA probabilities for reported pesticides classes and their Mass2Motifs 67

3.4 Case Study Natural Products **67**
3.4.1 Erinacerin family findings 68
3.4.2 Hericenone family findings 68
3.4.3 Ergostane family findings 69
3.4.4 Additional Mass2Motif annotations via motif-motif matching 70

3.5 Case Study on Accelerating Biological Discoveries with MAG-empowered MS2LDA **70**

3.6 Suspect list **71**
3.6.1 Peptide example 72
3.6.2 Steroid example 72
3.6.3. Sulfonamide example 72
3.6.4 Glycoside example 72

3.7 Handling large datasets within reasonable timeframes **73**

4. Supplementary Methods ***73***
4.1.1 Mass2Motif Object 73
4.1.2 MS2LDA 2.0 parameter selection and definition 73
4.1.3 MS2LDA 2.0 convergence curves 80
4.2.1 MotifDB 80
4.3.1 Visualization Interface 81
4.3.2 Run Analysis 81
4.3.3 Motif Ranking and Motif Details 82
4.3.4 Spectra Search 82
4.3.5 View Network 83
4.3.6 Motif Search 83

5. Supplementary Discussion ***84***

5.1 Parameter selection recommendations **84**

Supplementary References ***85***

1. *Supplementary Figures*

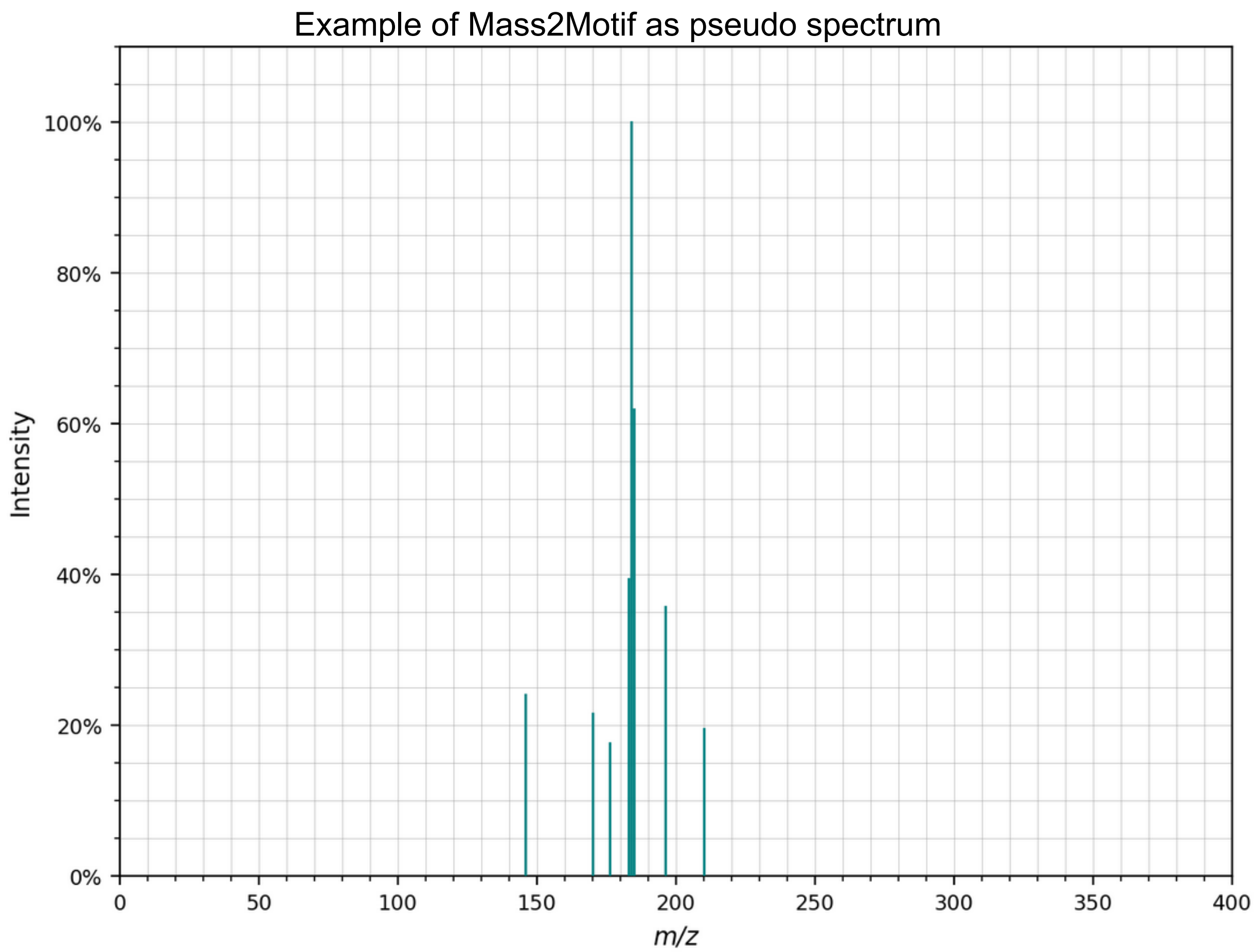

Fig. S1. Mass2Motif 219 from the case study on pesticides consists of nine mass fragments and one neutral loss (not shown; has no importance in this example).

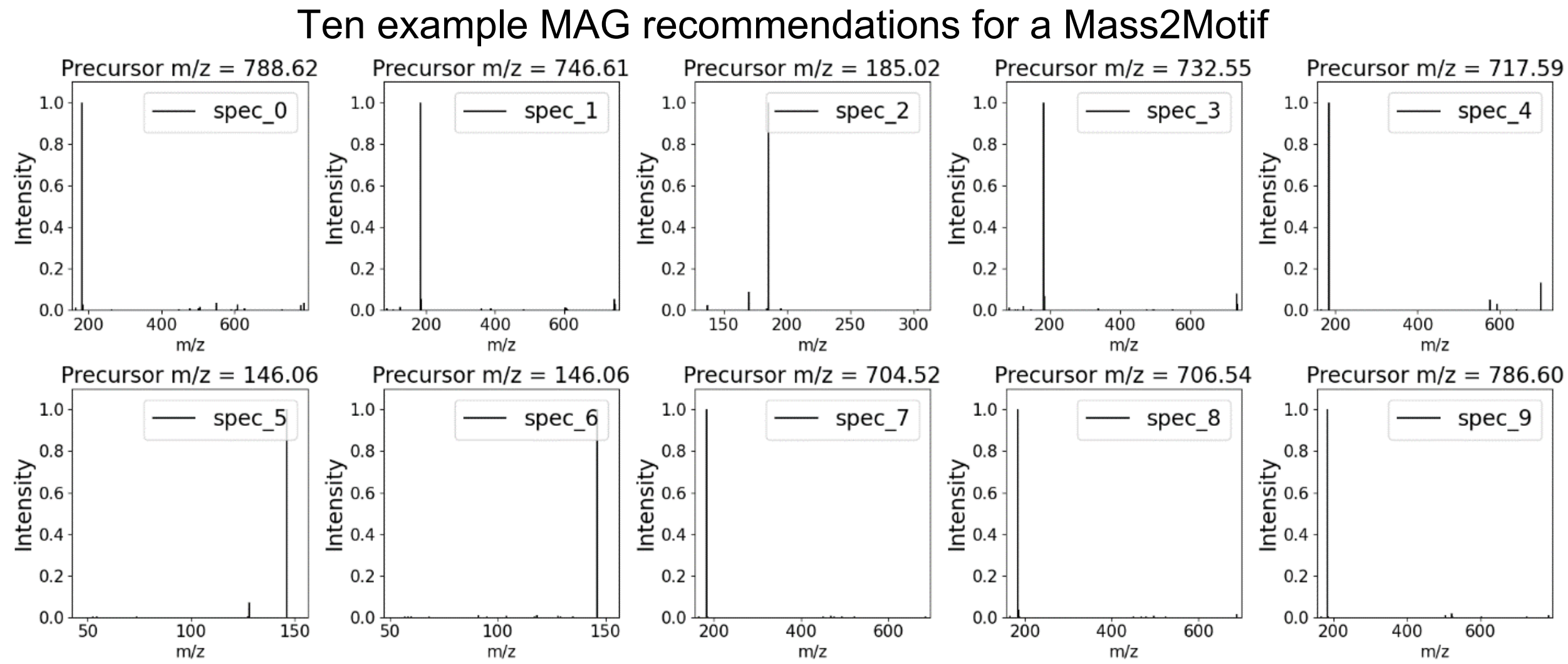

Fig. S2. Ten retrieved spectra based on the highest Spec2Vec scores (0.38-0.45) between a Mass2Motif and the reference database. The similarity score is the highest in the top left corner and the lowest in the bottom right corner.

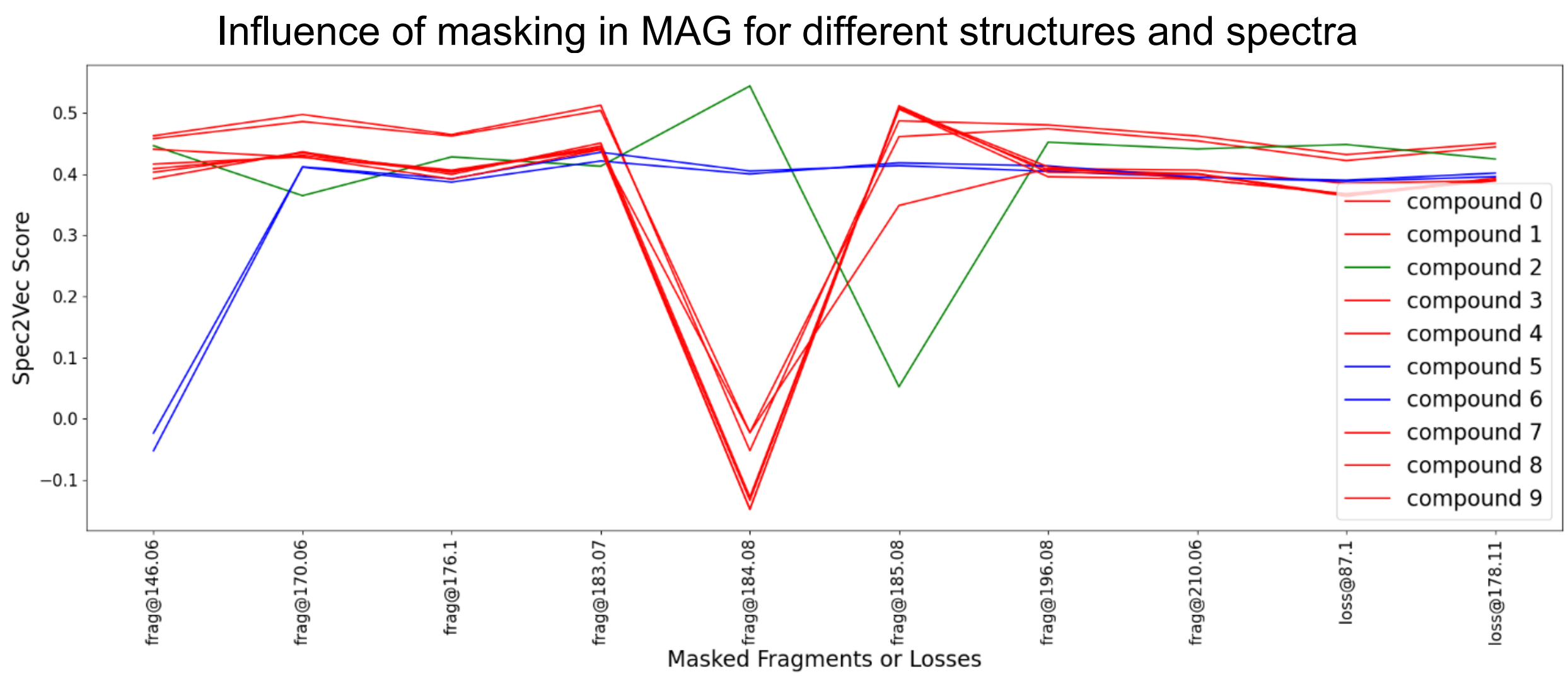

Fig. S3. The visualization of the masking results shows the effect on the Spec2Vec score for the top ten compound when a fragment or loss is removed from the Mass2Motif. The Figure already includes the clustering based on the masking, which splits the ten compounds in three clusters highlighted by different colors.

Top ten candidate structures from MAG without masking

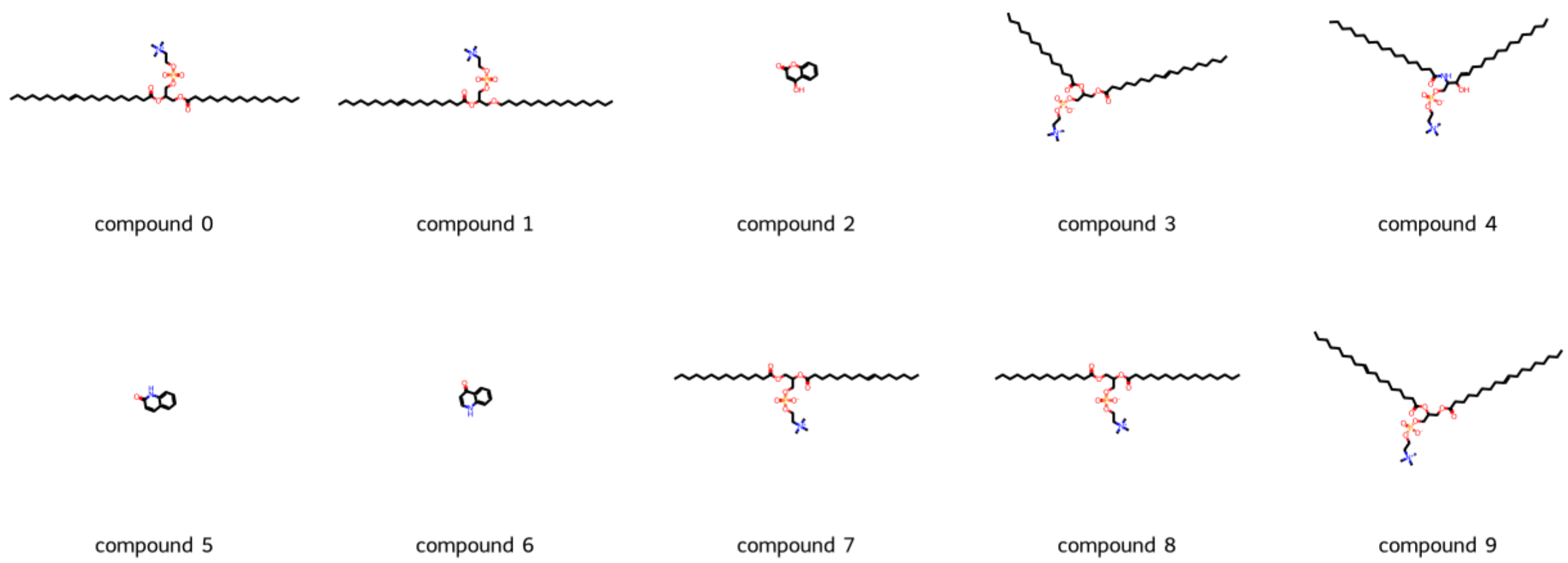

Fig. S4. Structural annotation of the top ten retrieved compounds from the reference database after computing Spec2Vec.

Example Mass2Motif visualization generated by MS2LDA 2.0

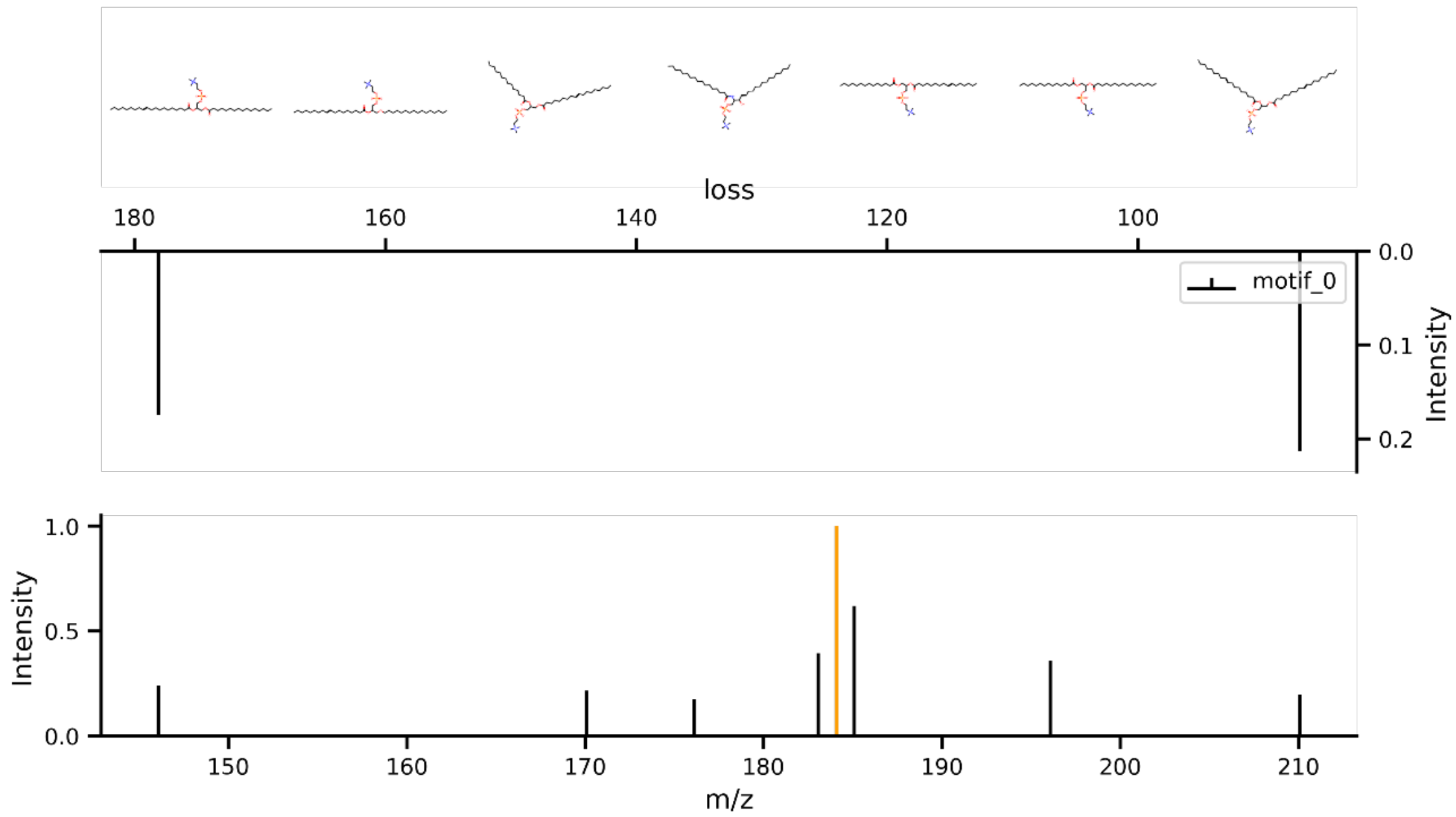

Fig. S5. Example of output, top panel shows structure recommendations from MAG. The bottom panel displays the fragments associated with this Mass2Motif (black) as a standard spectrum. The orange-highlighted fragment is consistently observed across all recommended structures and represents the characteristic fragmentation of the phosphate group. Mirrored above the fragments are the neutral losses related to the Mass2Motif, arranged from right to left in increasing mass difference from the precursor ion.

MAG benchmark for the Urine-derived MotifSet

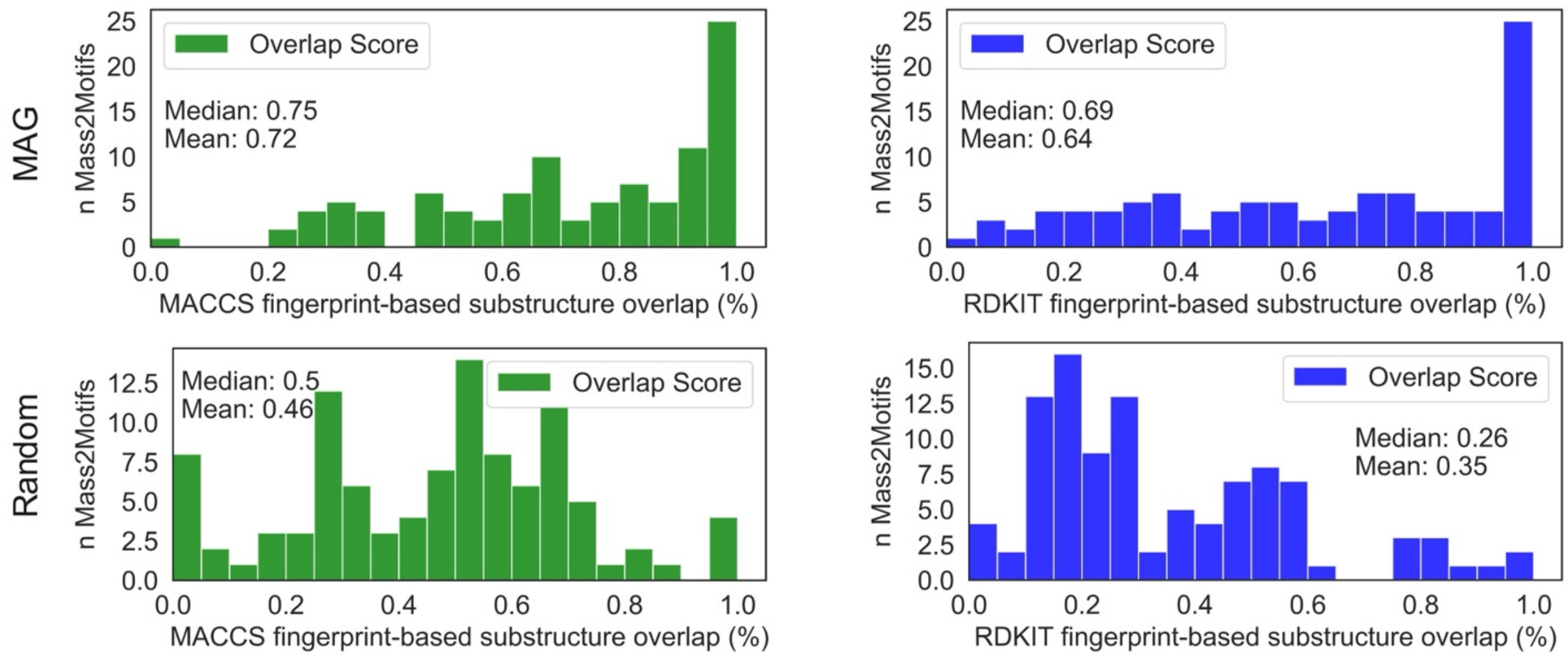

Fig. S6. Substructure overlap distribution of the scores with MACCS fingerprints (green) and RDKit fingerprints (blue) for MAG compared to randomly selected molecules for the urine-derived MotifSet. This represents how MAG outperforms the baseline of randomly selected molecules by 0.25.

MAG benchmark for the GNPS derived MotifSet

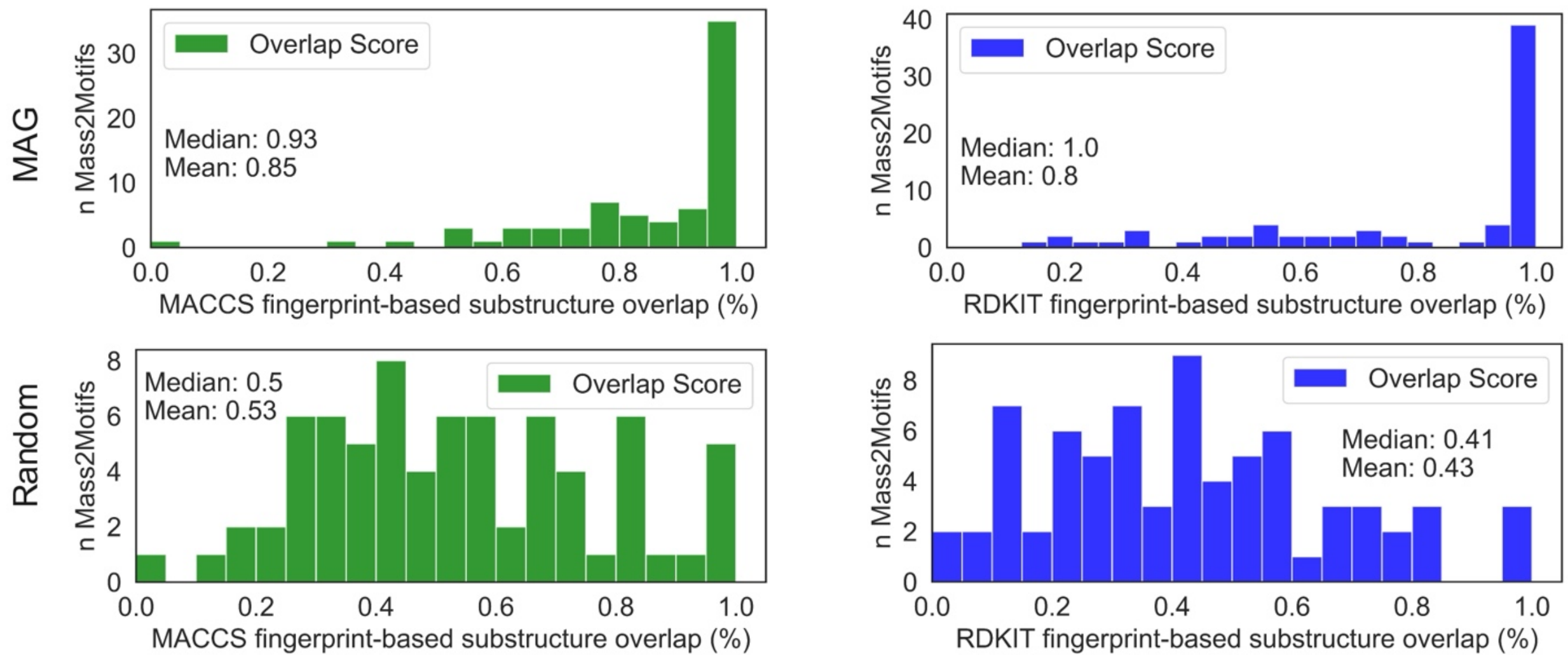

Fig. S7. Substructure overlap distribution of the scores with MACCS fingerprints (green) and RDKit fingerprints (blue) for MAG compared to randomly selected molecules for the GNPS MotifSet. This represents how MAG outperforms randomly selected molecules in terms of overlapping scores by 0.3.

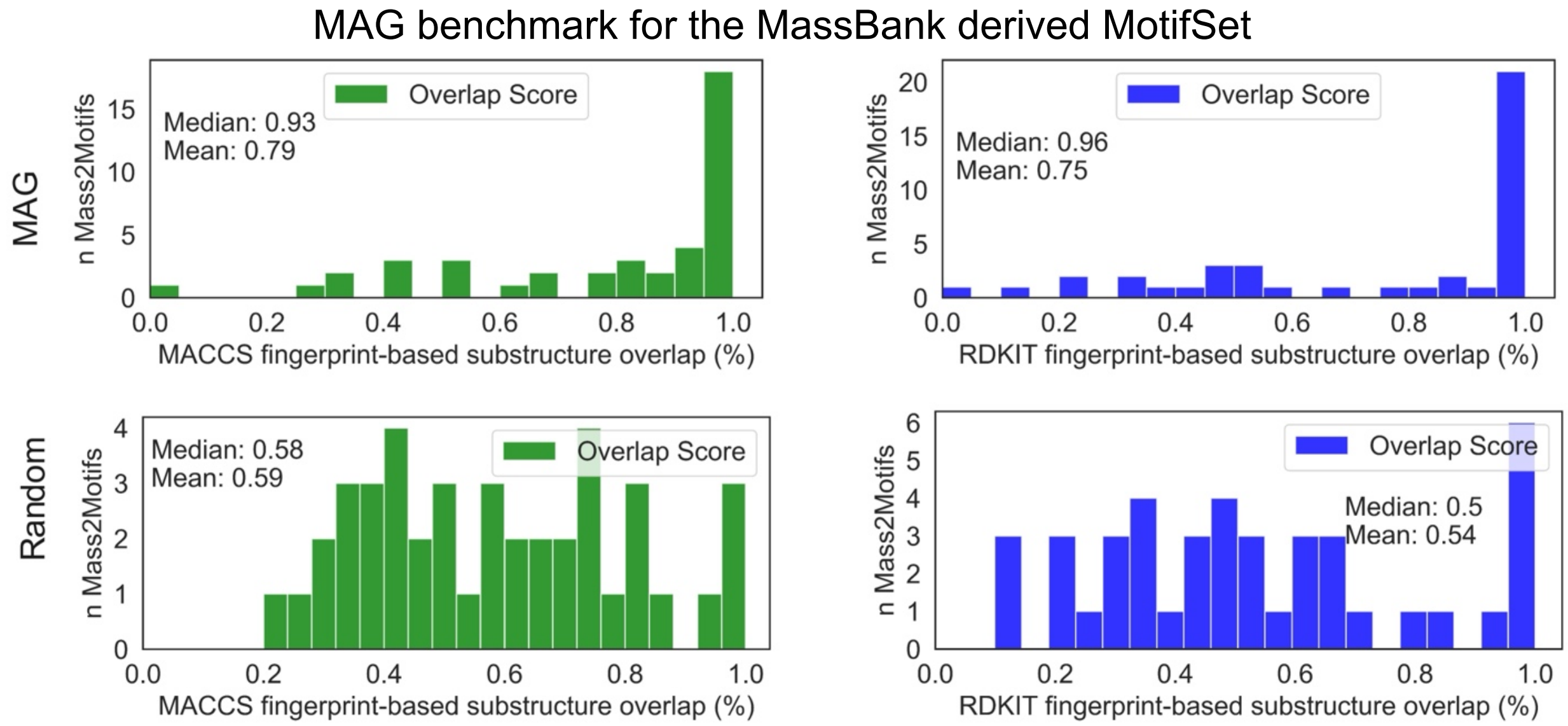

Fig. S8. Substructure overlap distribution of the scores with MACCS fingerprints (green) and RDKit fingerprints (blue) for MAG compared to randomly selected molecules for the MassBank MotifSet. This represents how MAG outperforms randomly selected molecules in terms of overlapping scores by 0.2.

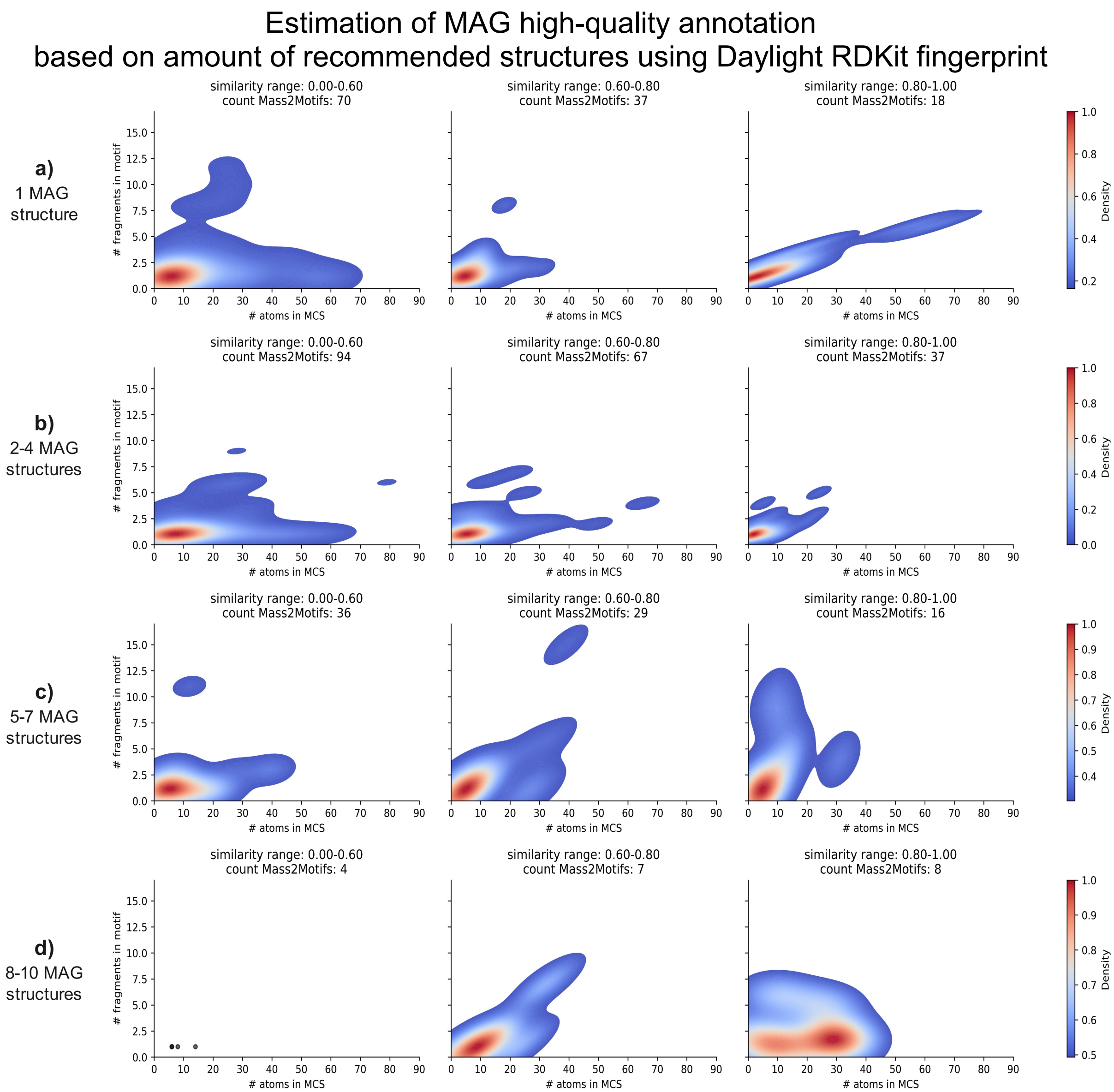

Fig. S9. All subplots show the distribution of Mass2Motifs annotations as a function of number of fragments (y-axis) and the number of MCS atoms (x-axis). The color scale indicates the density of annotations. Distributions are stratified by the SOS score (based on Daylight RDKit fingerprint) of the MAG annotation, grouped into three ranges: 0-0.6, 0.6-0.8, 0.8-1.0). a) one MAG recommendation makes it difficult to distinguish between high and low-quality annotations, because low-quality annotations have an overlapping distribution with high-quality annotations and the amount of low and high-quality annotations is also similar. b) for 2-4 MAG recommendations the distributions of low and high-quality annotations still overlap, but there are overall more high-quality annotations than low-quality annotations. c) 5-7 MAG recommendations allow for the first time to distinguish between high and low-quality annotation based on the amount fragments present. Mass2Motif with more than 5 fragments are mostly present when the SOS score is above 0.6. Additionally, the number of high-quality annotations is now substantially greater than the number of low-quality annotations. d) 8-10 MAG recommendations show mostly high-quality annotations.

Pesticide case study Mass2Motif for dimethylcarbamate

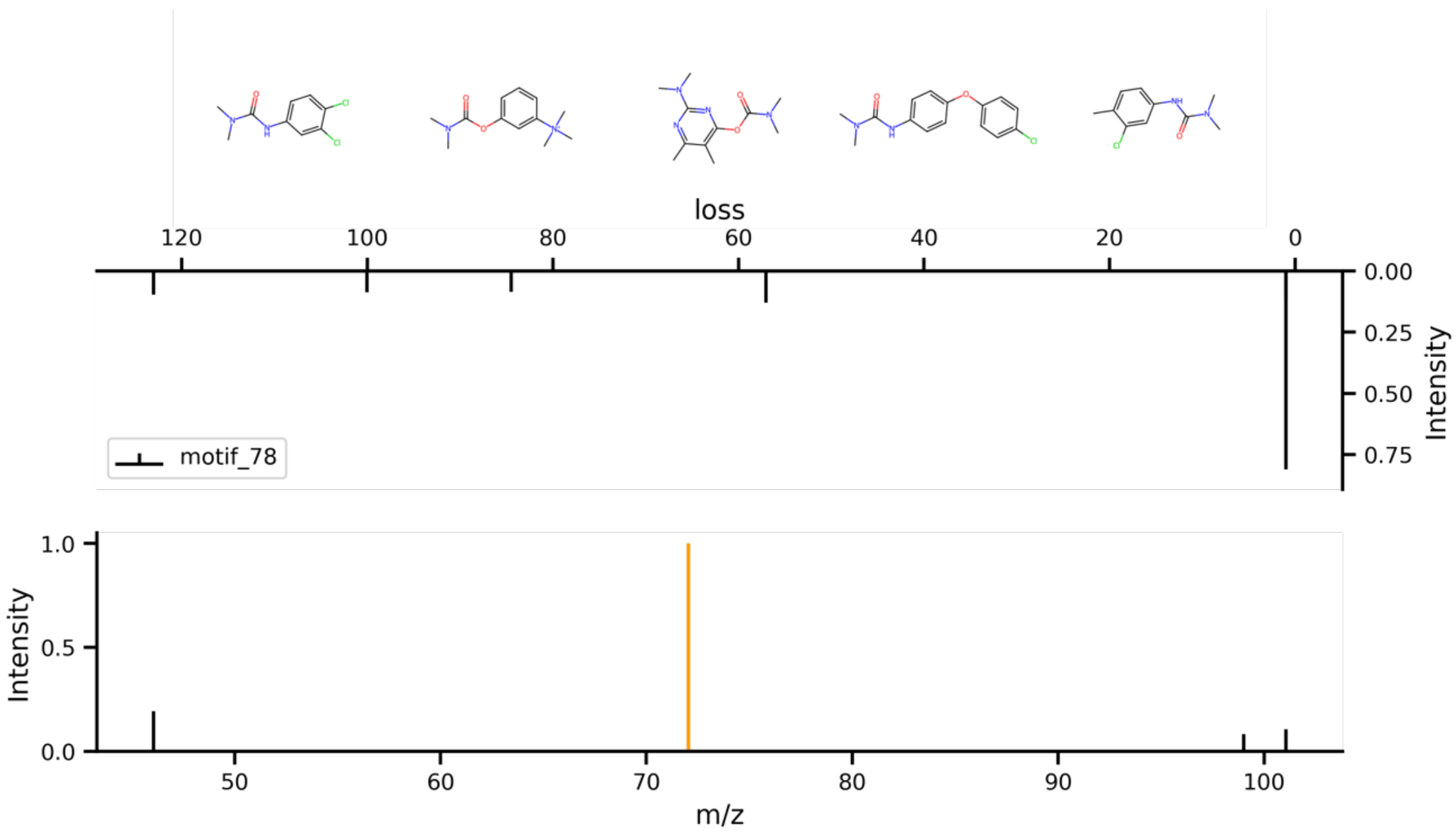

Fig. S10. Mass2Motif 78 is associated with dimethyl-carbamate substructures. The top panel shows structure recommendations from MAG, all featuring dimethyl-carbamate moieties. The bottom panel displays the fragments associated with this Mass2Motif as a standard MS/MS spectrum. The orange-highlighted fragment at m/z 72.04 is consistently observed across all recommended structures and represents the characteristic fragmentation of the dimethyl-carbamate group. Mirrored above the fragments are the corresponding neutral losses, arranged from right to left in increasing mass difference from the precursor ion.

Pesticide case study Mass2Motif for triazole pesticides

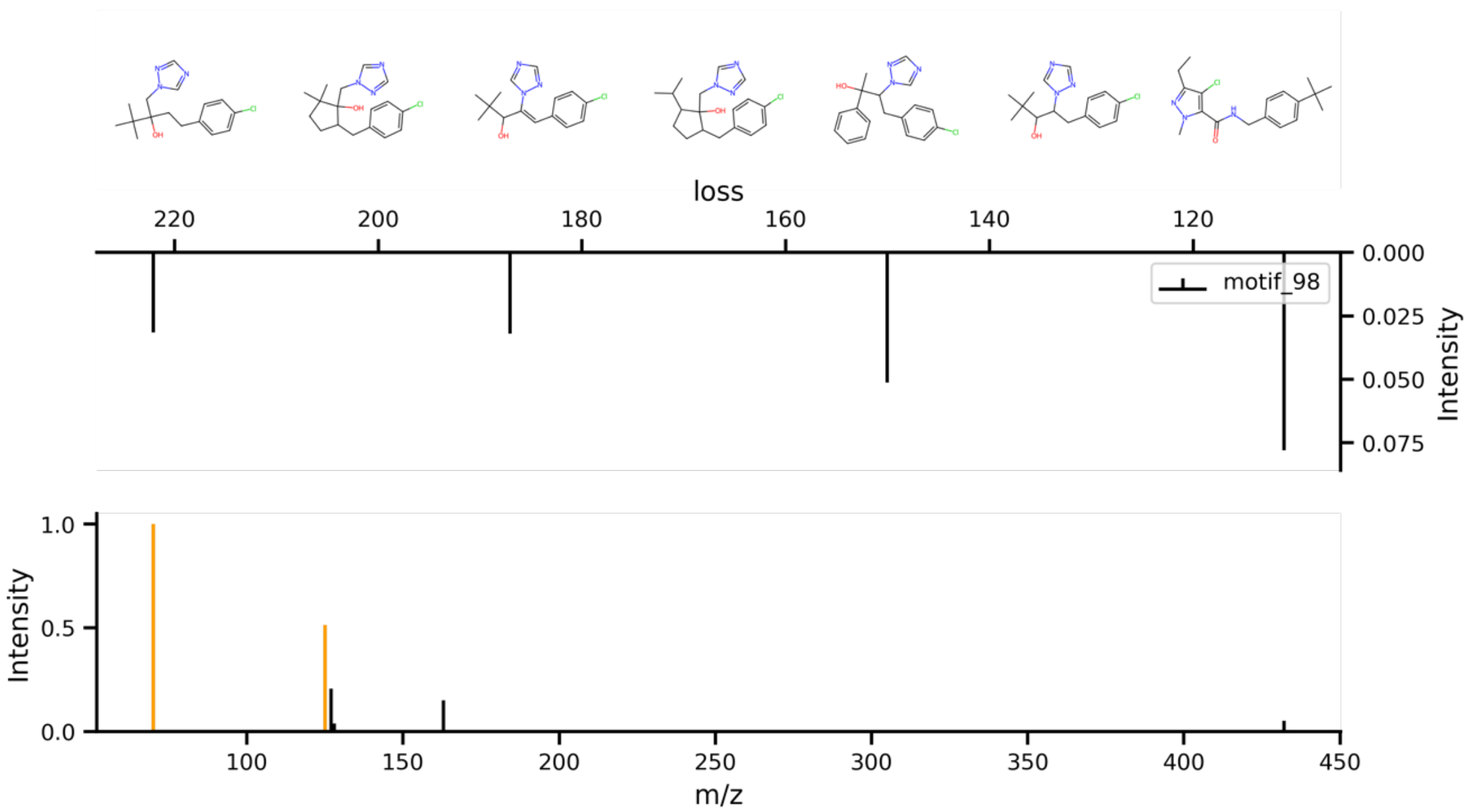

Fig. S11. Mass2Motif 98 represents a common pesticide related pattern of two substructures. At the top the structure recommendations by MAG are shown. Below are the intensities of the losses related to the Mass2Motif and it is read from the precursor from right to left. At the bottom the fragments related to the Mass2Motif are shown as a normal spectrum plot. The orange highlighted fragments of 70.04 and 125.02 is present in all the spectra from structure recommendations from the top.

Mass2Motif 137 (pyridine backbone) from erinacerin family

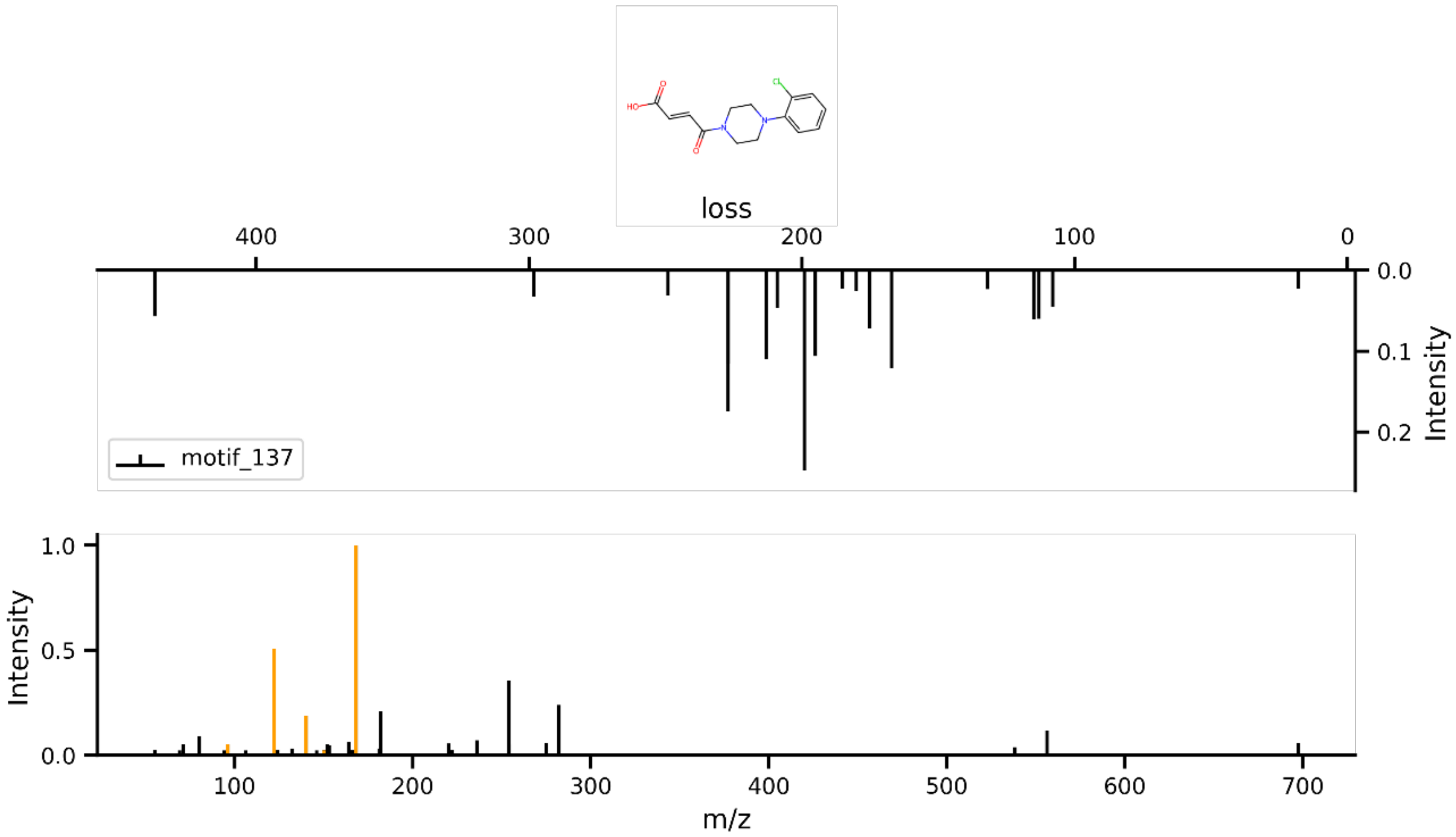

Fig. S12. Mass2Motif 137 is associated with a pyridine backbone. The top panel shows the only structure from MAG. The bottom panel displays the fragments associated with this Mass2Motif as a standard MS/MS spectrum. The orange-multiple highlighted fragments are consistently observed in the recommended structure. Mirrored above the fragments are the corresponding neutral losses, arranged from right to left in increasing mass difference from the precursor ion.

Mass2Motif 22 (2 (propylidenamino-) acetic acid moiety) from erinacerin family

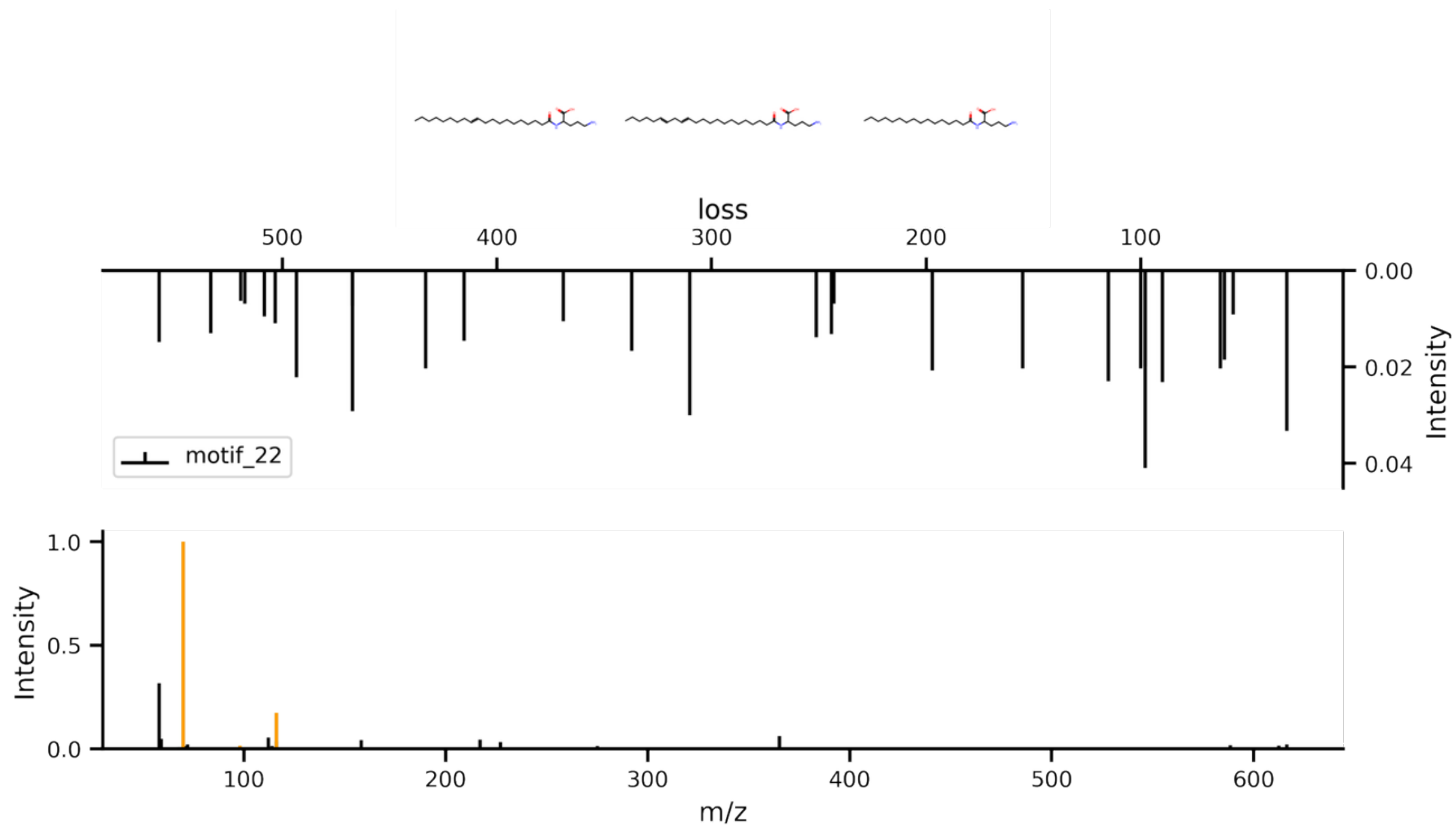

Fig. S13. Mass2Motif 22 is associated with a 2 (propylidenamino-) acetic acid moiety. The top panel shows the only structure from MAG. The bottom panel displays the fragments associated with this Mass2Motif as a standard MS/MS spectrum. The orange-peaks highlighted 70.07 and 116.11 m/z are consistently observed in the recommended structures. Mirrored above the fragments are the corresponding neutral losses, arranged from right to left in increasing mass difference from the precursor ion.

Mass2Motif 108 (indole moiety) from erinacerin family

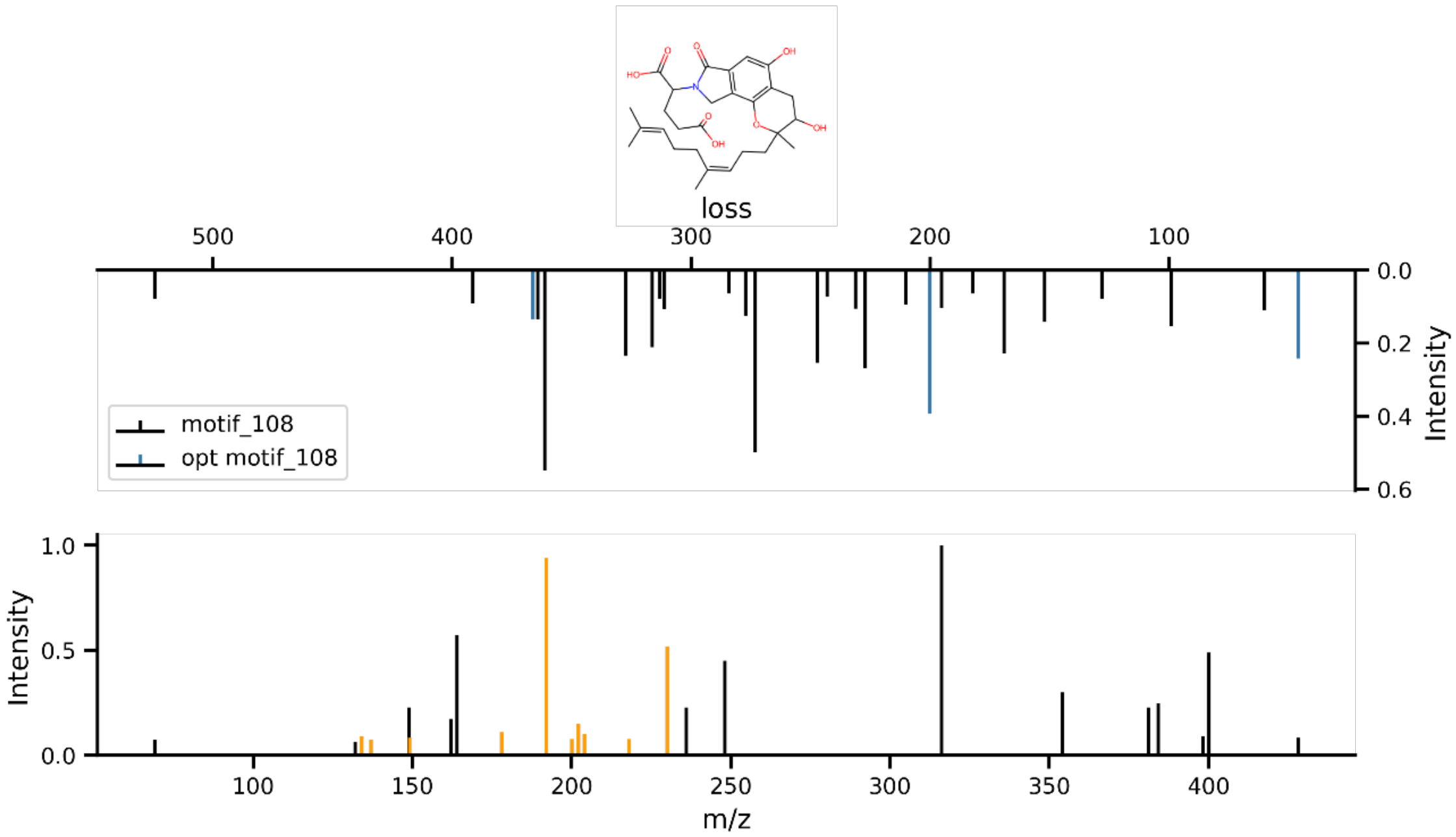

Fig. S14. Mass2Motif 108 is associated with an indole substructure. The top panel shows the only structure from MAG. The bottom panel displays the fragments associated with this Mass2Motif as a standard MS/MS spectrum. The orange-multiple highlighted fragments are consistently observed in the recommended structure. Mirrored above the fragments are the corresponding neutral losses, arranged from right to left in increasing mass difference from the precursor ion.

Mass2Motif network and pattern matching substructures in the hericenone family

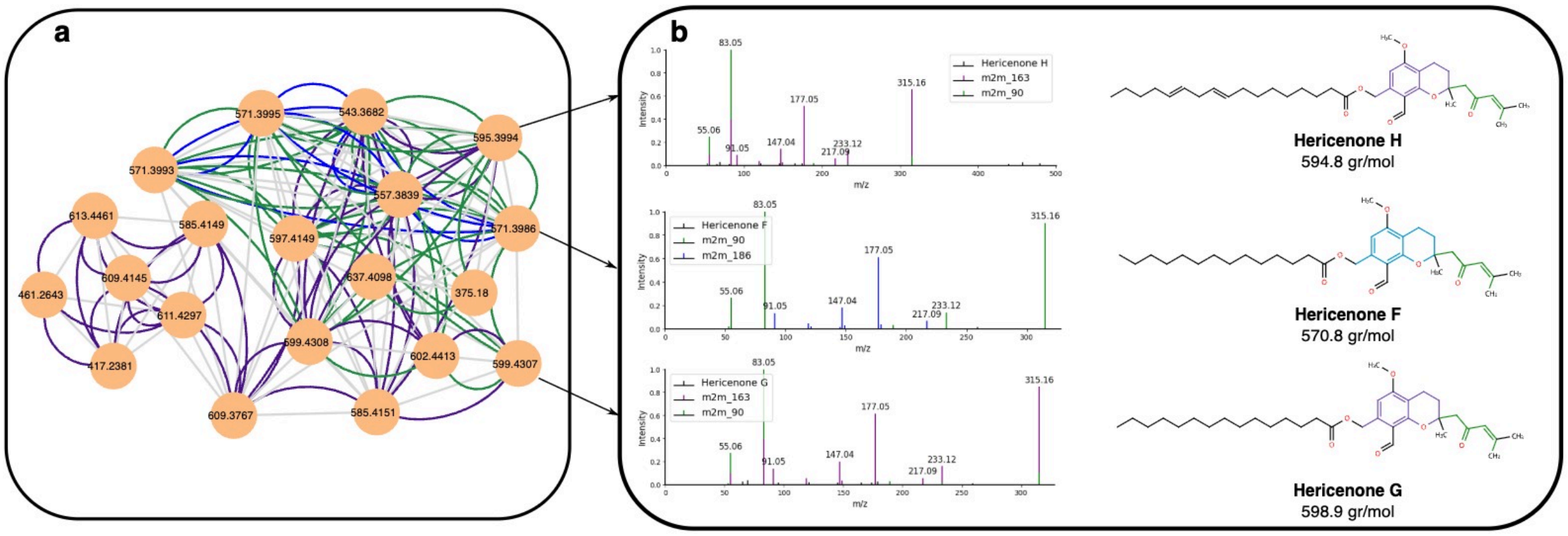

Fig. S15. MS2LDA 2.0 results from the fungal natural product dataset for the hericenone family. a) Molecular family from hericenones originated reported by Kathib et al [7]. b) Spectra of the molecules manually annotated as hericenone H, F and G respectively with the Mass2Motifs related fragments colored on top. Structures of the complete hericenone molecules with the inferred Mass2Motifs by MAG colored. We note that the data (mass spectra and molecular network) were originally generated by Khatib et al. (2025), while this network figure with shared Mass2Motifs was generated during this study (for further details check Code Availability).

Mass2Motif 163 (benzodihydropyran substructure) from hericenone family

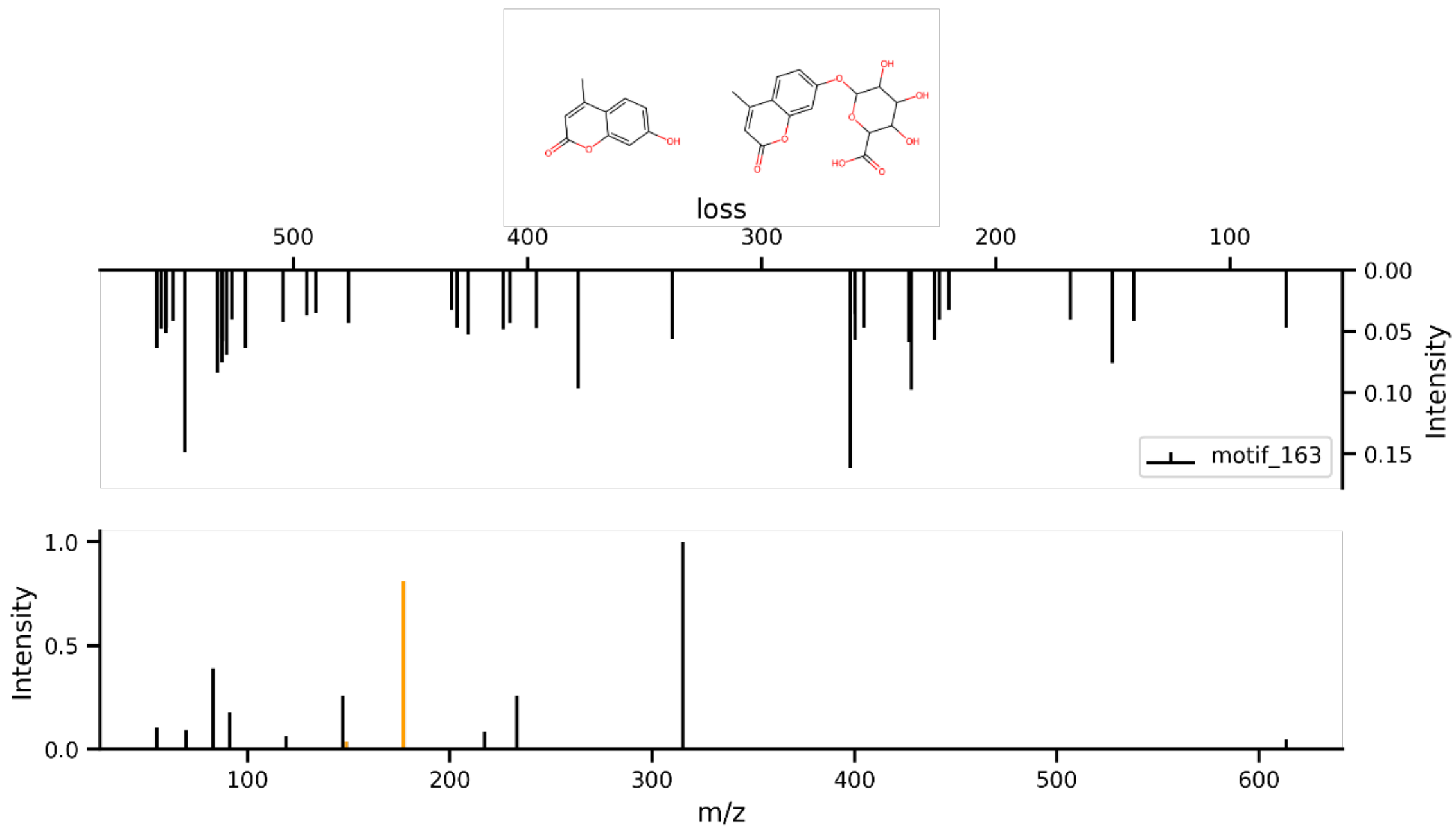

Fig. S16. Mass2Motif 163 is associated with benzodihydropyran substructures. The top panel shows structure recommendations from MAG, all featuring benzodihydropyran moieties. The bottom panel displays the fragments associated with this Mass2Motif as a standard MS/MS spectrum. The orange-highlighted fragment at m/z 177.05 is consistently observed across all recommended structures and represents the characteristic fragmentation of the benzodihydropyran substructure. Mirrored above the fragments are the corresponding neutral losses, arranged from right to left in increasing mass difference from the precursor ion.

Mass2Motif 186 (benzodihydropyran substructure) from hericenone family

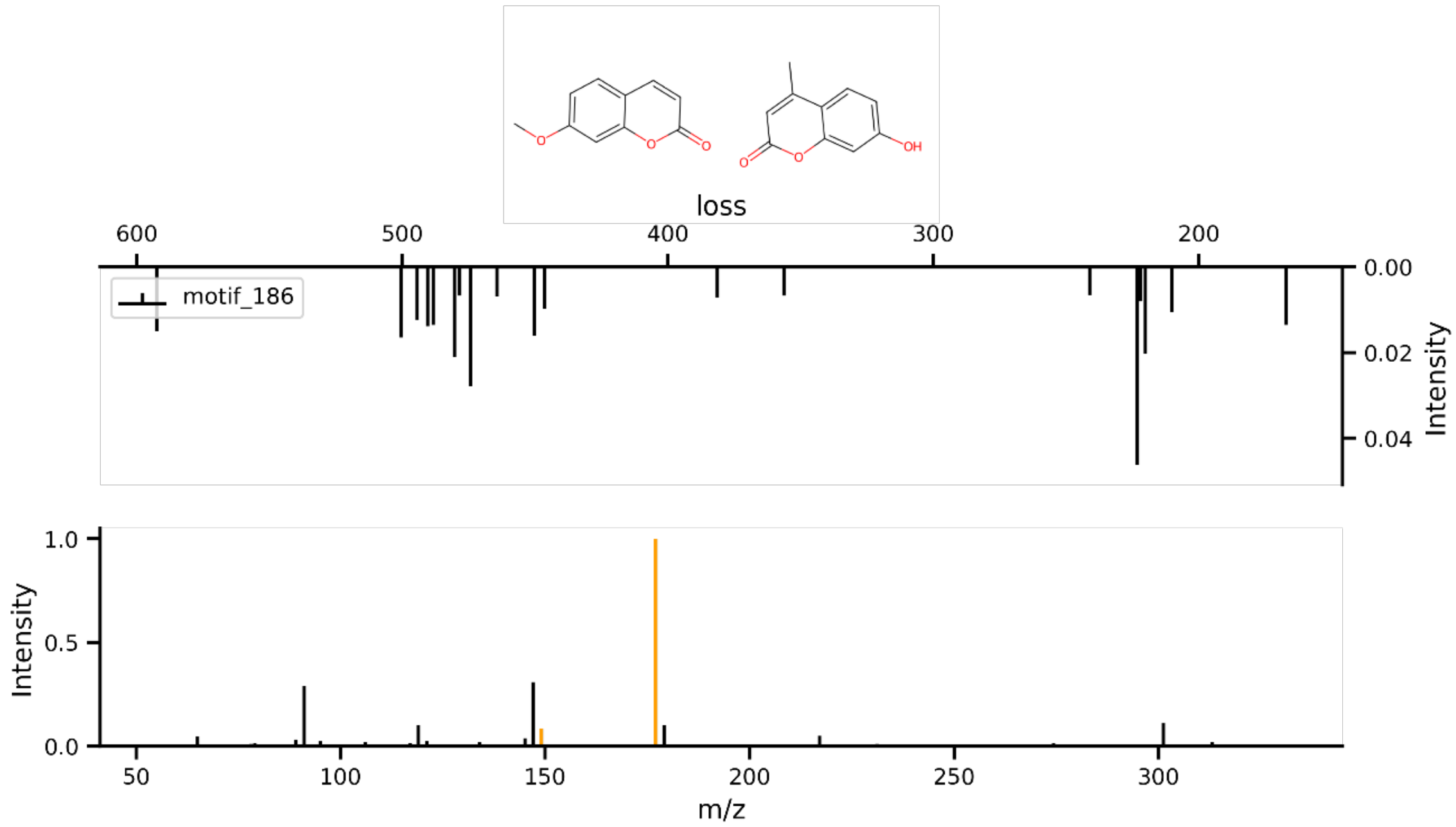

Fig. S17. Mass2Motif 186 is associated with benzodihydropyran substructures. The top panel shows structure recommendations from MAG, all featuring benzodihydropyran moieties. The bottom panel displays the fragments associated with this Mass2Motif as a standard MS/MS spectrum. The orange-highlighted fragments at m/z 177.05 and 149.06 are consistently observed across all recommended structures and represents the characteristic fragmentation of the benzodihydropyran substructure. Mirrored above the fragments are the corresponding neutral losses, arranged from right to left in increasing mass difference from the precursor ion.

Mass2Motif 90 (2-(2-methylbut-2-enamido)acetic and 4-fluoro-2-(4-hydroxyoxan-3-yl)-5-methyl-6-[(4-pyrazol-1-ylphenyl)methyl]-3H-isoindol-1-one structures) from hericenone family

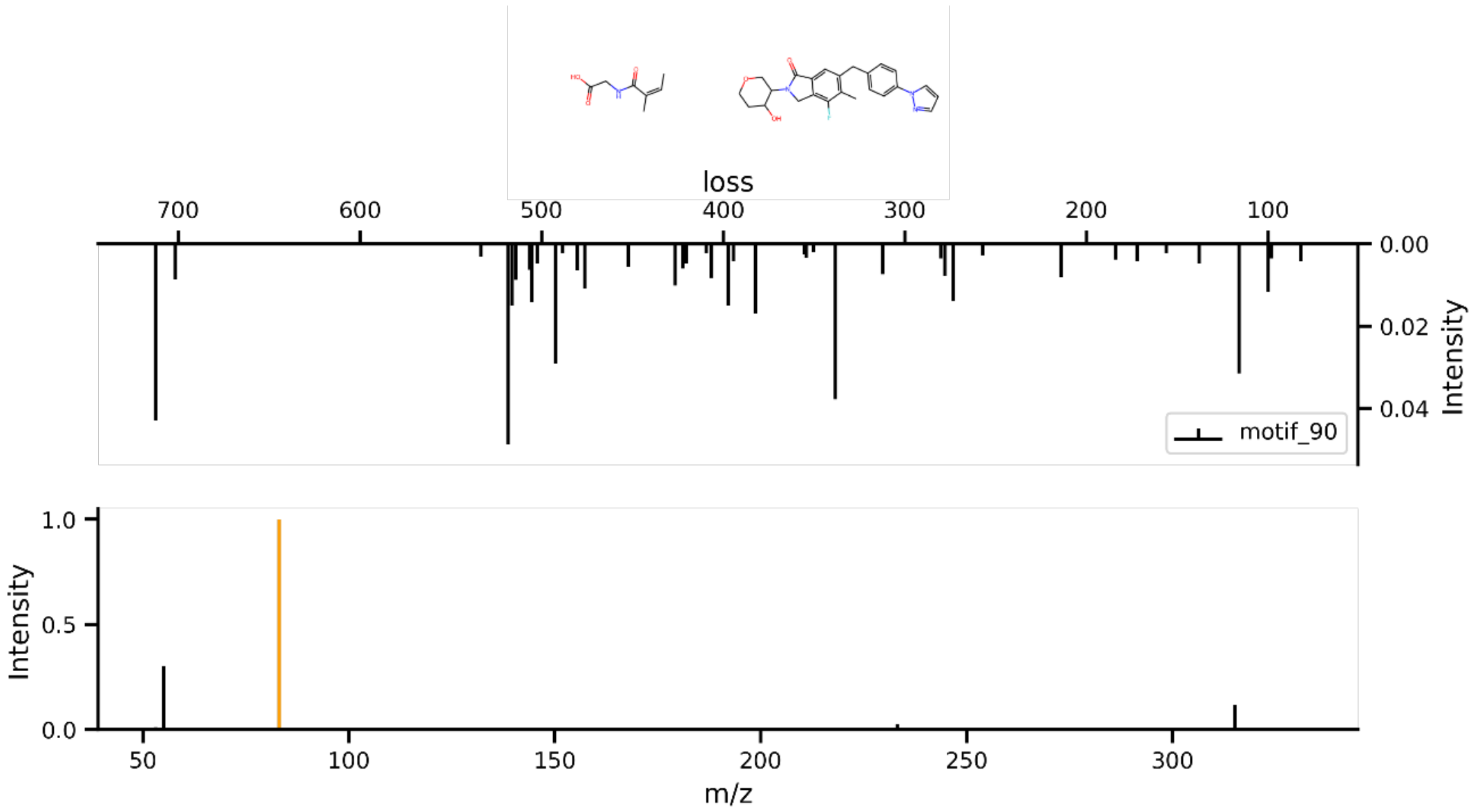

Fig. S18. Mass2Motif 90 is associated with 2-(2-methylbut-2-enamido)acetic and 4-fluoro-2-(4-hydroxyoxan-3-yl)-5-methyl-6-[(4-pyrazol-1-ylphenyl)methyl]-3H-isoindol-1-one structures. The top panel shows structure recommendations from MAG. The bottom panel displays the fragments associated with this Mass2Motif as a standard MS/MS spectrum. The orange-highlighted fragment at m/z 83.05 is consistently observed across both recommended structures. Mirrored above the fragments are the corresponding neutral losses, arranged from right to left in increasing mass difference from the precursor ion.

Mass2Motif network and pattern matching substructures in the ergostane family

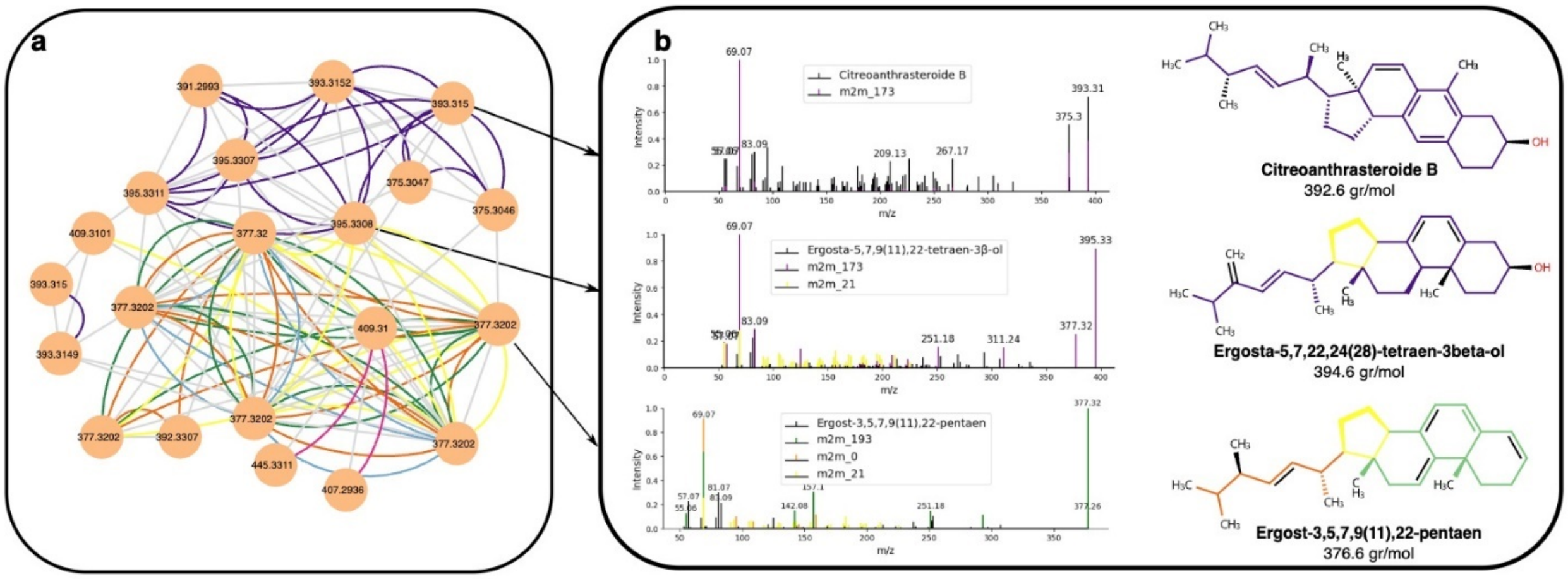

Fig. S19. MS2LDA 2.0 results from the fungal natural product dataset for the ergostane family. a) Molecular family from ergostane originated reported by Khatib et al.[7], b) Spectra of the molecules manually annotated as citreoanthrasteroide B, Ergosta-5,7,22,24(28)-tetraen-3beta-ol and ergost-3,5,7,9(11),22-pentaen with their Mass2Motifs related fragments colored on top. Structures of the complete ergostane molecules with the inferred Mass2Motifs by MAG colored. We note that the data (mass spectra and molecular network) were originally generated by Khatib et al. (2025), while this network figure with shared Mass2Motifs was generated during this study (for further details check Code Availability).

Mass2Motif 173 (steroid scaffold) from ergostane family

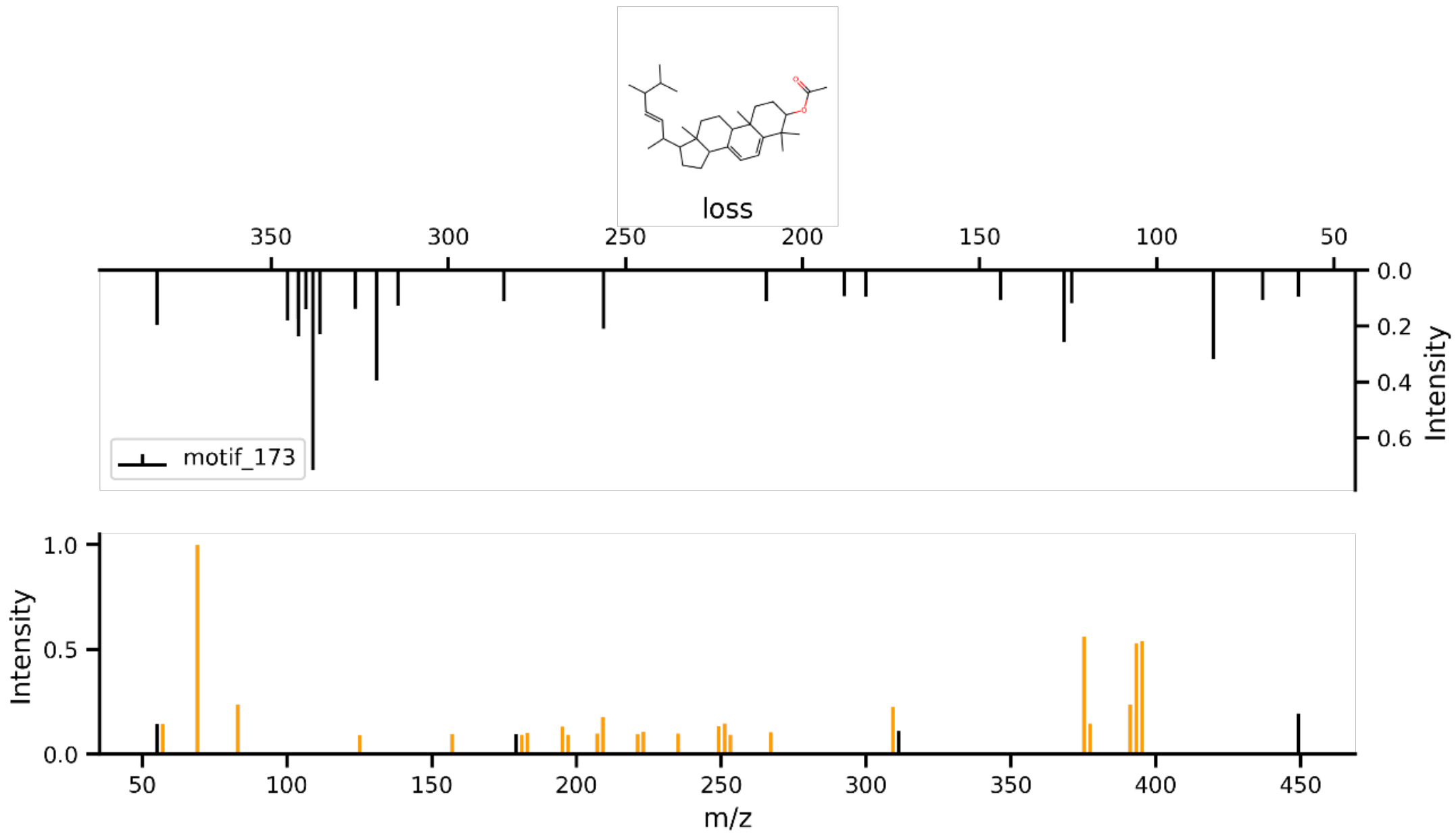

Fig. S20. Mass2Motif 173 is associated with a steroid scaffold and a carbon side chain. The top panel shows structure recommendations from MAG. The bottom panel displays the fragments associated with this Mass2Motif as a standard MS/MS spectrum. The orange-multiple highlighted fragments are consistently observed across both recommended structures. Mirrored above the fragments are the corresponding neutral losses, arranged from right to left in increasing mass difference from the precursor ion.

Mass2Motif 21 (2,15-dihydroxy-7-methyl-6-oxabicyclo[11.3.0]hexadeca-3,11-dien-5-one substructure) from ergostane family

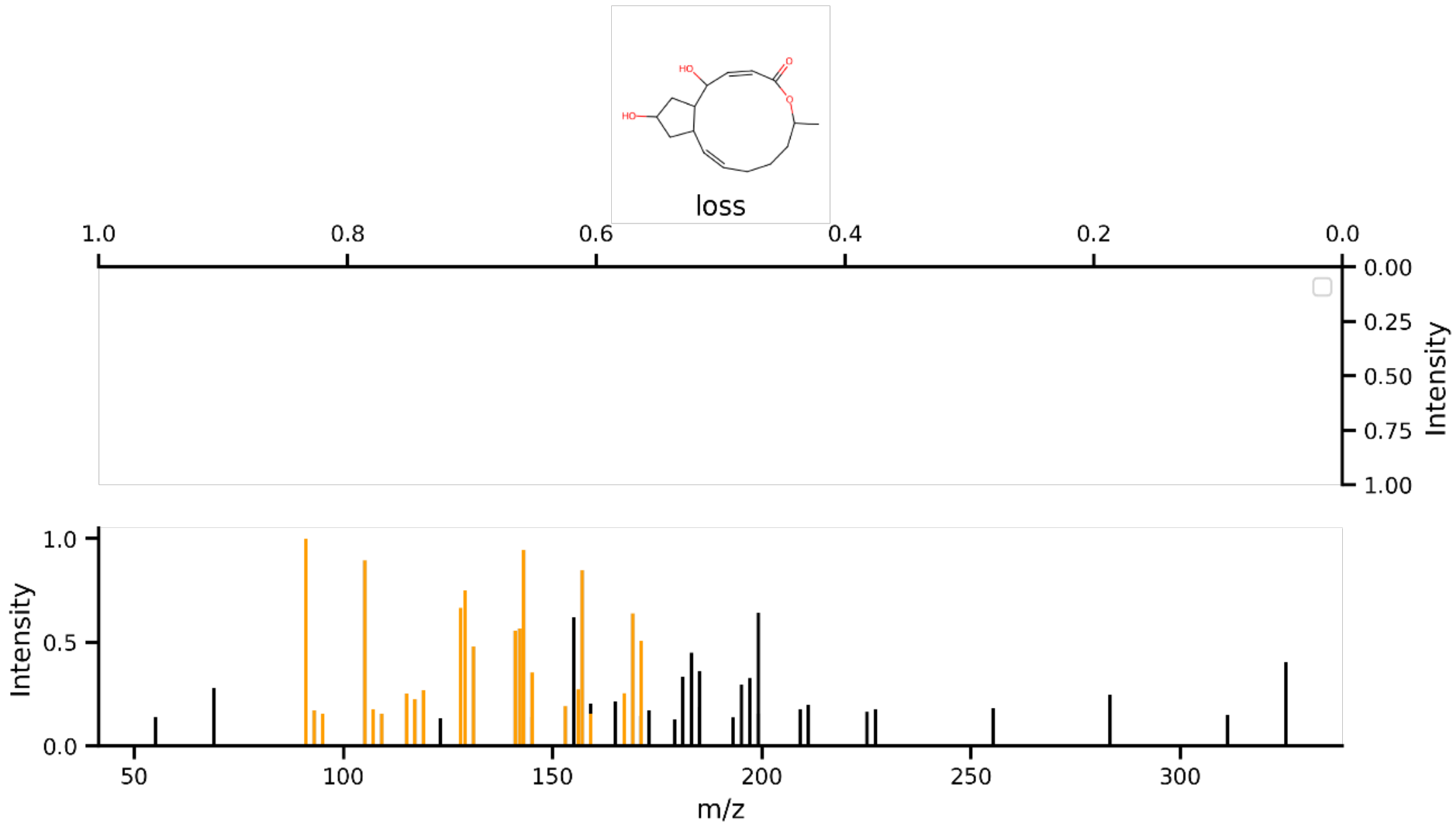

Fig. S21. Mass2Motif 21 is associated with 2,15-dihydroxy-7-methyl-6-oxabicyclo[11.3.0]hexadeca-3,11-dien-5-one. The top panel shows the only structure from MAG. The bottom panel displays the fragments associated with this Mass2Motif as a standard MS/MS spectrum. The orange-multiple highlighted fragments are consistently observed in the recommended structure. Mirrored above the fragments are the corresponding neutral losses (empty), arranged from right to left in increasing mass difference from the precursor ion.

Mass2Motif 0 (2,15-dihydroxy-7-methyl-6-oxabicyclo[11.3.0]hexadeca-3,11-dien-5-one substructure) from ergostane family

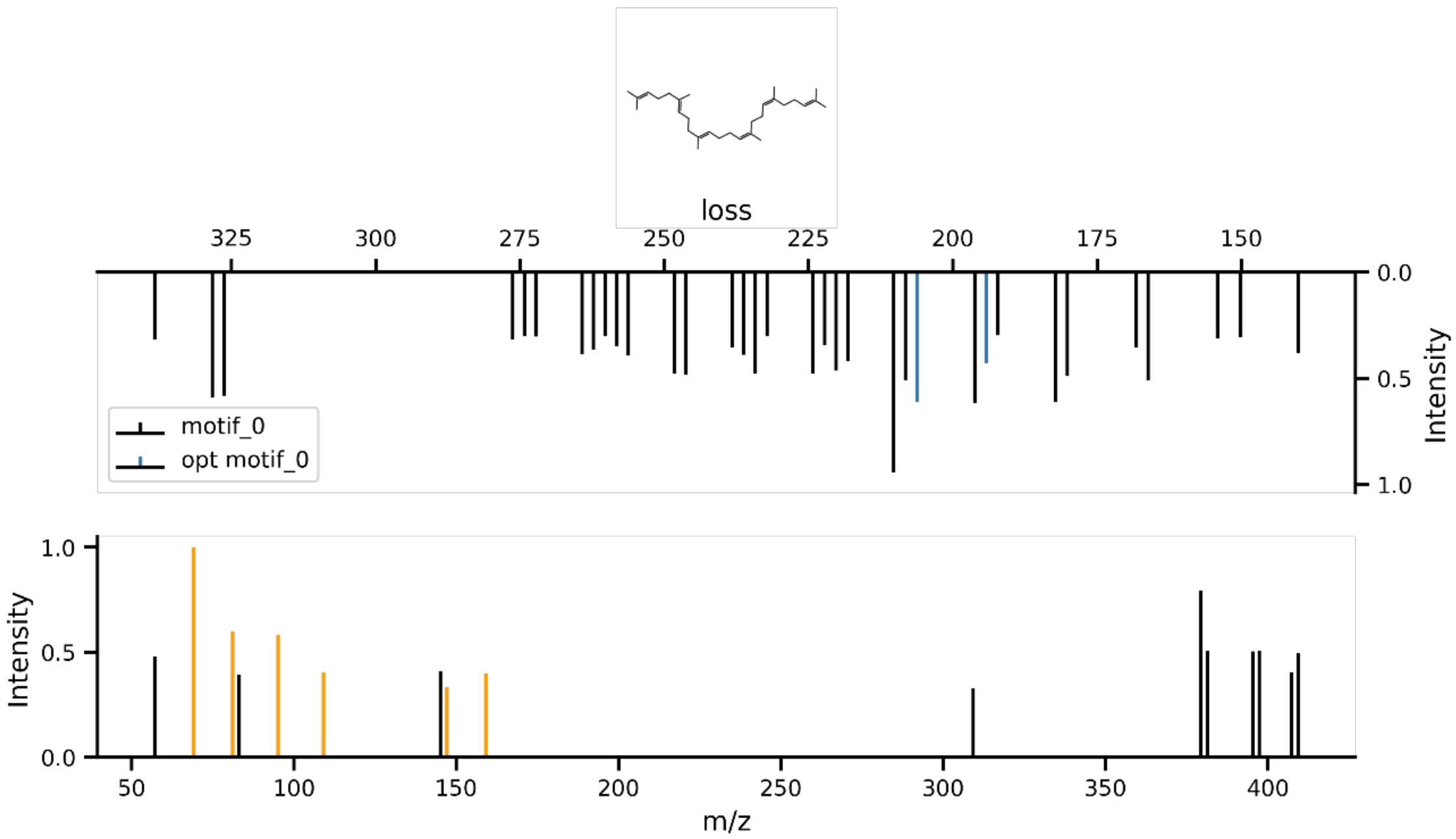

Fig. S22. Mass2Motif 0 is associated with 2,15-dihydroxy-7-methyl-6-oxabicyclo[11.3.0]hexadeca-3,11-dien-5-one. The top panel shows the only structure recommended by MAG. The bottom panel displays the fragments associated with this Mass2Motif as a standard MS/MS spectrum. The orange-multiple highlighted fragments are observed in the recommended structure. Mirrored above the losses of 206.2 and 194.2 m/z are also part of the recommended structure. Losses are arranged from right to left in increasing mass difference from the precursor ion.

Mass2Motif 193 (steroid scaffold) from ergostane family

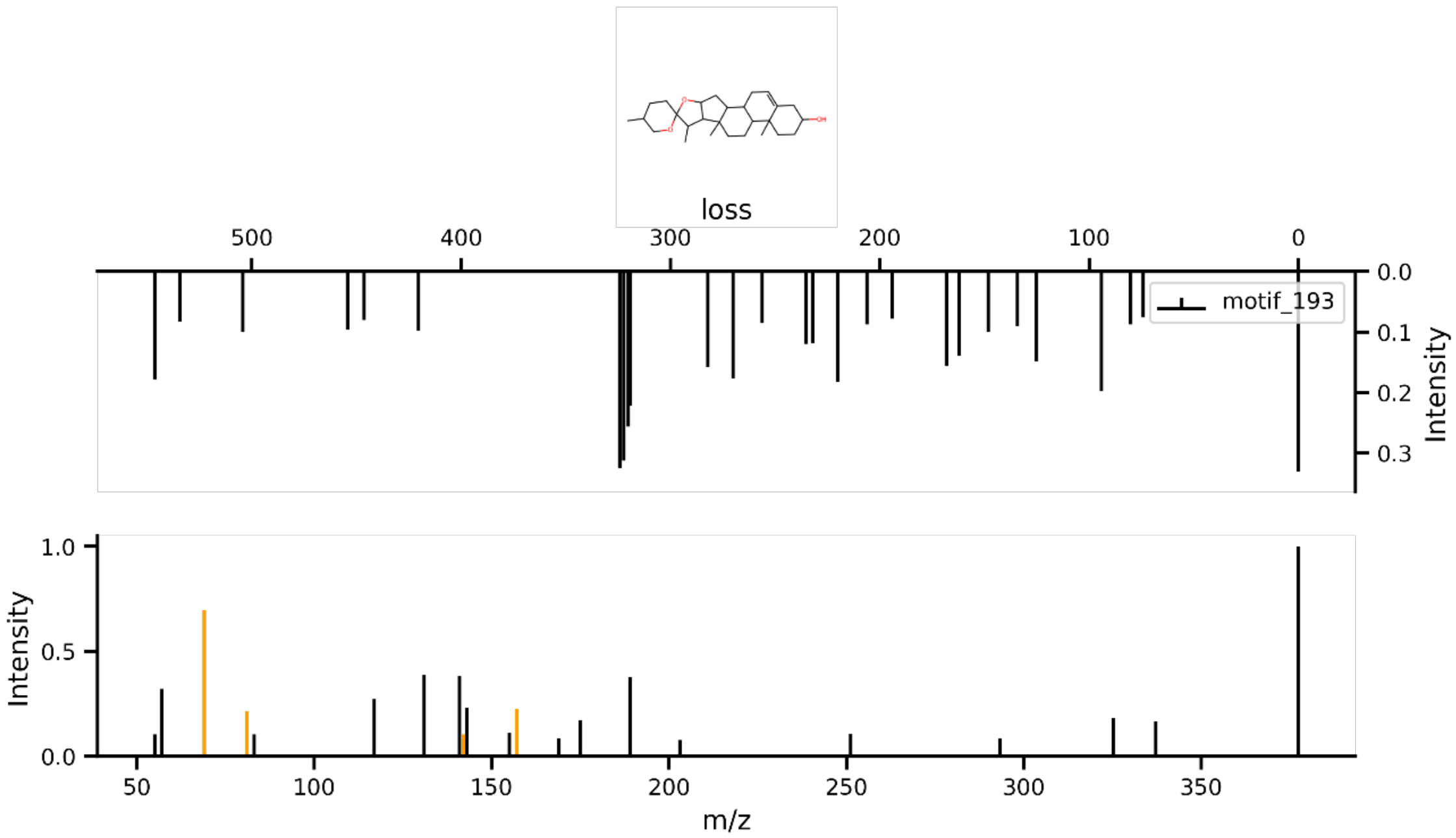

Fig. S23. Mass2Motif 193 is associated with a steroid backbone. The top panel shows the only structure from MAG. The bottom panel displays the fragments associated with this Mass2Motif as a standard MS/MS spectrum. The orange-multiple highlighted fragments are consistently observed in the recommended structure. Mirrored above the fragments are the corresponding neutral losses, arranged from right to left in increasing mass difference from the precursor ion.

Mass2Motif 10 MAG fungi case study recommendations

Motif Details: motif_10

Spec2Vec Matching Results

The Spec2Vec matching results displayed here suggest chemical structures (SMILES strings) that closely match the selected motif. Spec2Vec calculates similarities by comparing motif pseudo-spectra against reference spectra from a known database. Matches shown here can help identify possible chemical identities or provide clues about structural characteristics represented by this motif.

Spec2Vec Matching Results

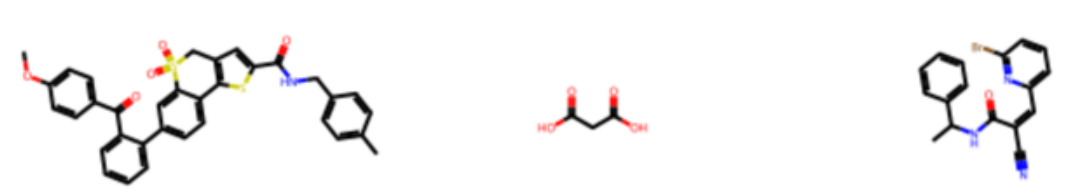

Match 1 Match 2 Match 3

Auto Annotations: ['COc1ccc(C(=O)c2ccccc2-c2ccc3c(c2)S(=O)(=O)Cc2cc(C(=O)NCc4ccc(C)cc4)sc2-3)cc1', 'O=C(O)CC(=O)O', 'CC(NC(=O)C(C#N)=Cc1cccc(Br)n1)c1ccccc1']

Screening Annotations

Ref Motif ID	Ref ShortAnno	Ref MotifSet	Score
motif_13	Alkyl aromatic substructure â€" indicative for aromatic ring...	Massbank library derived Mass2Motifs	0.7742
motif_11	Alkyl aromatic substructure â€" indicative for aromatic ring...	GNPS library derived Mass2Motifs	0.7742
motif_23	No short annotation available	LDB MotifDB POS	0.1723
motif_86	No short annotation available	LDB MotifDB POS	0.1473
motif_50	Steroid core related (C18H21 and smaller fragments thereof -...	GNPS library derived Mass2Motifs	0.1460

Fig. S24. Mass2Motif 10 details shown using the app. The matching results show the structure recommendations from MAG. The bottom panel displays the Mass2Motif-matching results, with their respective Spec2Vec scores. This inspection shows that it is highly probable that Mass2Motif 10 contains an aromatic ring as MAG recommendation and Mass2Motif-matching align.

Mass2Motif 126 MAG fungi case study recommendations

Motif Details: motif_126

Spec2Vec Matching Results

The Spec2Vec matching results displayed here suggest chemical structures (SMILES strings) that closely match the selected motif. Spec2Vec calculates similarities by comparing motif pseudo-spectra against reference spectra from a known database. Matches shown here can help identify possible chemical identities or provide clues about structural characteristics represented by this motif.

Spec2Vec Matching Results

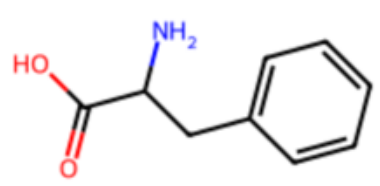

Match 1

Auto Annotations: ['NC(Cc1ccccc1)C(=O)O']

Screening Annotations

Ref Motif ID	Ref ShortAnno	Ref MotifSet	Score
motif_59	Fragments indicative for [phenylalanine-CHOOH] based substru...	GNPS library derived Mass2Motifs	0.7574
motif_37	Fragments indicative for [phenylalanine-CHOOH] based substru...	Massbank library derived Mass2Motifs	0.7574
motif_91	No short annotation available	LDB MotifDB POS	0.6007
motif_97	No short annotation available	LDB MotifDB POS	0.1407
motif_85	No short annotation available	LDB MotifDB POS	0.1319

Fig. S25. Mass2Motif 126 details shown using the app. The matching results show the only structure recommendation from MAG. The bottom panel displays the Mass2Motif-matching results, with their Spec2Vec respective scores. This inspection shows the MAG and Mass2Motif recommendation align, assigning Mass2Motif 126 phenylalanine containing-substructure.

Mass2Motif 61 MAG fungi case study recommendations

Motif Details: motif_61

Spec2Vec Matching Results

The Spec2Vec matching results displayed here suggest chemical structures (SMILES strings) that closely match the selected motif. Spec2Vec calculates similarities by comparing motif pseudo-spectra against reference spectra from a known database. Matches shown here can help identify possible chemical identities or provide clues about structural characteristics represented by this motif.

Spec2Vec Matching Results

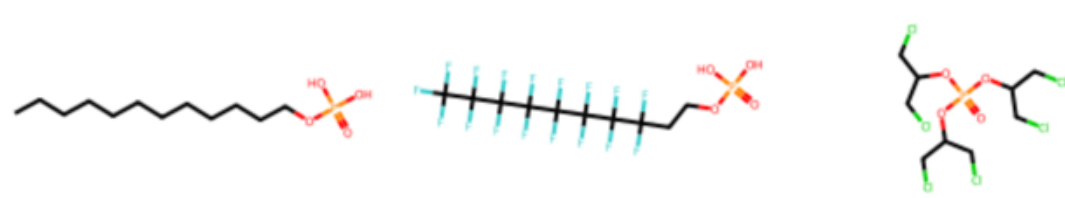

Match 1 Match 2 Match 3

Auto Annotations: ['CCCCCCCCCCCCCOP(=O)(O)O', 'O=P(O)(O)OCCC(F)(F)C(F)(F)C(F)(F)C(F)(F)C(F)(F)C(F)(F)C(F)(F)C(F)(F)F', 'O=P(OC(CCl)CCl)(OC(CCl)CCl)OC(CCl)CCl']

Screening Annotations

Ref Motif ID	Ref ShortAnno	Ref MotifSet	Score
motif_48	Fragment ion indicative for conjugation of a phosphate group...	GNPS library derived Mass2Motifs	0.7079
motif_26	Fragment ion indicative for the presence of a phosphate grou...	Massbank library derived Mass2Motifs	0.7079
motif_1	Fragment of C5H10NO2 and loss of C5H9NO2 (in beer likely to ...	Massbank library derived Mass2Motifs	0.1527
motif_4	Fragment and loss of [proline-H2O] - indicative for conjugat...	GNPS library derived Mass2Motifs	0.1527
motif_90	No short annotation available	LDB MotifDB POS	0.1249

Fig. S26. Mass2Motif 61 details shown using the app. The matching results show the structure recommendations from MAG. All of them contain a phosphate group. The bottom panel displays the Mass2Motif-matching results, with their Spec2Vec respective scores. This inspection shows the MAG and Mass2Motif positively align, assigning Mass2Motif 126 phosphate containing-substructure.

Mass2Motif 156 MAG fungi case study recommendations

Motif Details: motif_156

Spec2Vec Matching Results

The Spec2Vec matching results displayed here suggest chemical structures (SMILES strings) that closely match the selected motif. Spec2Vec calculates similarities by comparing motif pseudo-spectra against reference spectra from a known database. Matches shown here can help identify possible chemical identities or provide clues about structural characteristics represented by this motif.

Spec2Vec Matching Results

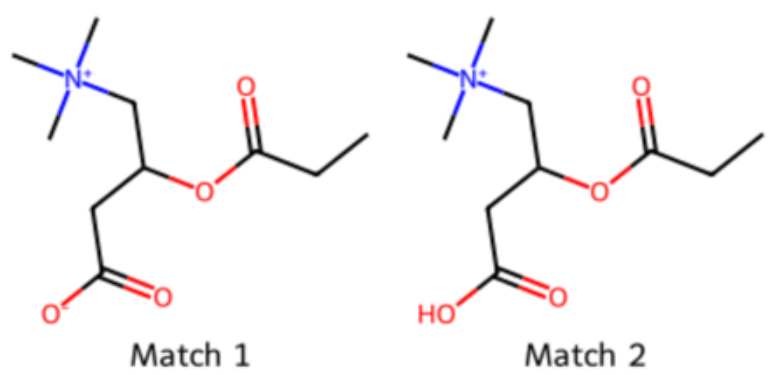

Auto Annotations: ['CCC(=O)OC(CC(=O)[O-])C[N+](C)(C)C', 'CCC(=O)OC(CC(=O)O)C[N+](C)(C)C']

Screening Annotations

Ref Motif ID	Ref ShortAnno	Ref MotifSet	Score
motif_45	Fragments indicative of a glycosylation â€“ i.e. indicative ...	GNPS library derived Mass2Motifs	0.7671
motif_24	Fragments indicative of a glycosylation â€“ i.e. indicative ...	Massbank library derived Mass2Motifs	0.7671
motif_23	No short annotation available	LDB MotifDB POS	0.2647
motif_42	Fragments indicative for kaempferol/glycosylated kaempferol ...	Massbank library derived Mass2Motifs	0.2430
motif_39	No short annotation available	LDB MotifDB POS	0.1678

Fig. S27. Mass2Motif 156 details shown using the app. The matching results show the structure recommendations from MAG. The structures shown are identical, containing an methylammonium group and a carboxylic group. The bottom panel displays the Mass2Motif-matching results, with their Spec2Vec respective scores. This inspection shows the MAG and Mass2Motif do not align, as the manual annotation assign a glycosylation for this Mass2Motif.

Mass2Motif 156 sugar loss from fungi case study

Motif Features Table and Summary Plots

The table below lists the motif features (fragments and losses) that pass the probability filter, including their probabilities within the motif. Below it, a bar plot shows how frequently each feature appears **within the filtered set of documents** for this motif (i.e., documents whose doc-topic probability and overlap score both pass the current threshold ranges).

Motif Features Table

Feature	Probability
frag@85.03	0.4927
loss@27.96	0.0404
loss@133.11	0.0398
loss@161.1	0.0340
frag@57.03	0.0269
frag@145.05	0.0247
frag@60.08	0.0238
loss@366.24	0.0196
loss@234.07	0.0196
frag@323.26	0.0196

Fig. S28. Mass2Motif 156 inspection suing the app. The table shows the fragments and losses representing this Mass2Motif. This Mass2Motig is represented by a loss of 161.1 m/z, that could be connected to a glycosylation.

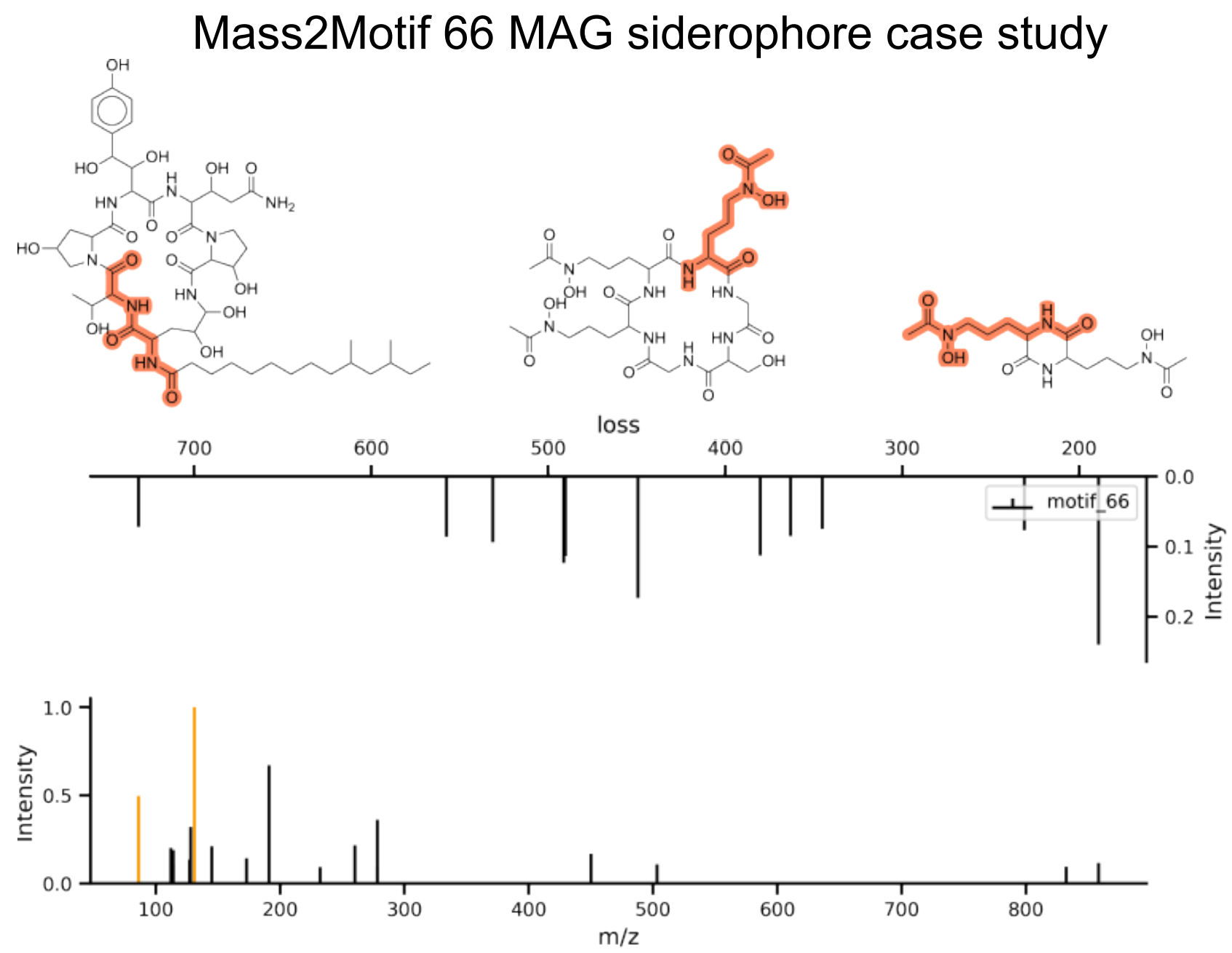

Fig. S29. Mass2Motif 66 is associated with a N-acetyl-N-hydroxy-ornithine substructure. The top panel shows the three recommended structures MAG returned. The bottom panel displays the fragments associated with this Mass2Motif as a standard MS/MS spectrum. The orange highlighted fragments are consistently observed in the recommended structure. Mirrored above the fragments are the corresponding neutral losses, arranged from right to left in increasing mass difference from the precursor ion.

Mass2Motif 34 MAG siderophore case study

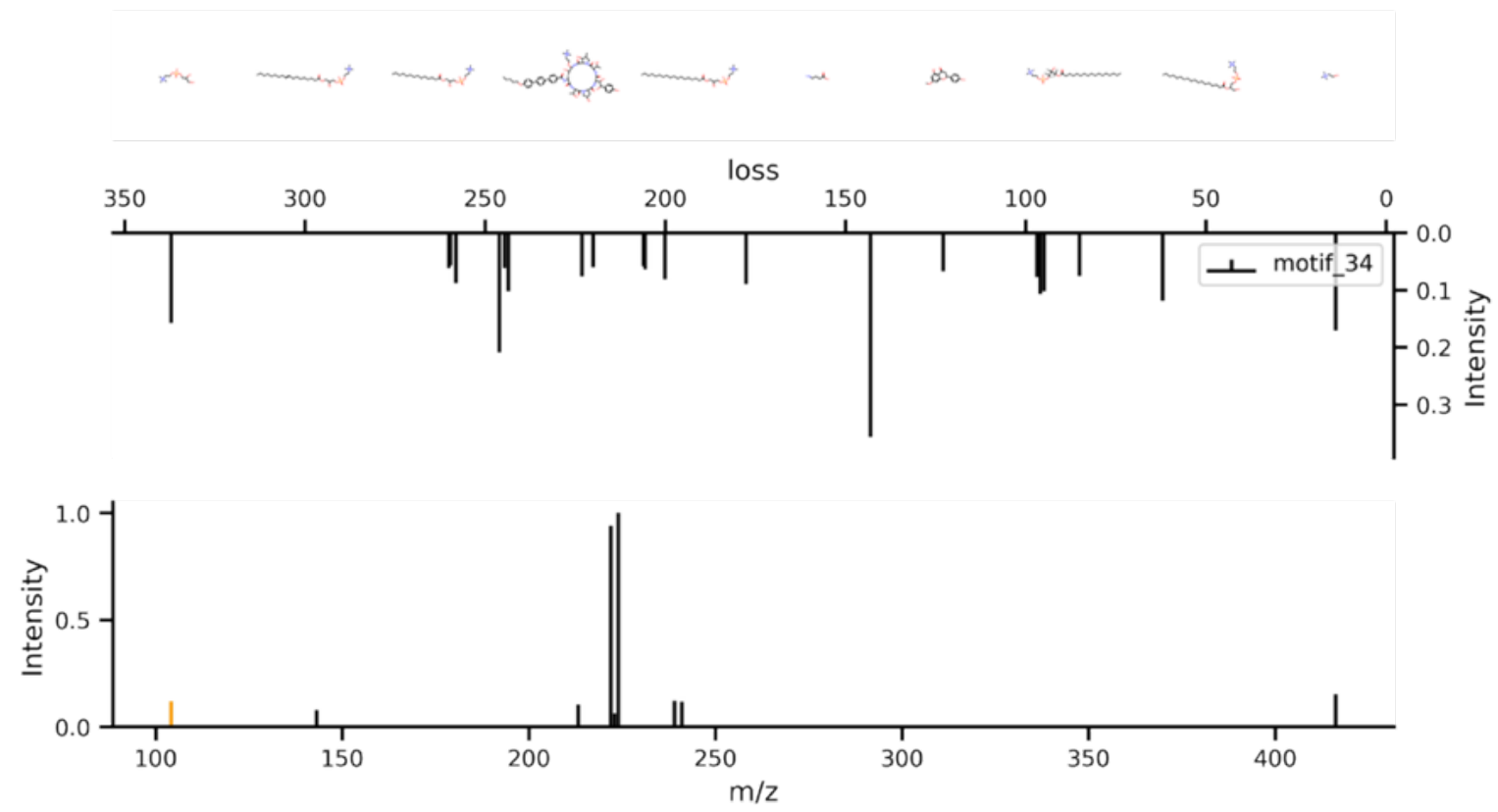

Fig. S30. Mass2Motif 34 is associated with bromotryptamine-like spectral features. The top panel shows the only structure from MAG. The bottom panel displays the fragments associated with this Mass2Motif as a standard MS/MS spectrum. The orange highlighted fragment is consistently observed in the recommended structure. Mirrored above the fragments are the corresponding neutral losses, arranged from right to left in increasing mass difference from the precursor ion.

Mass2Motif 129 MAG siderophore case study

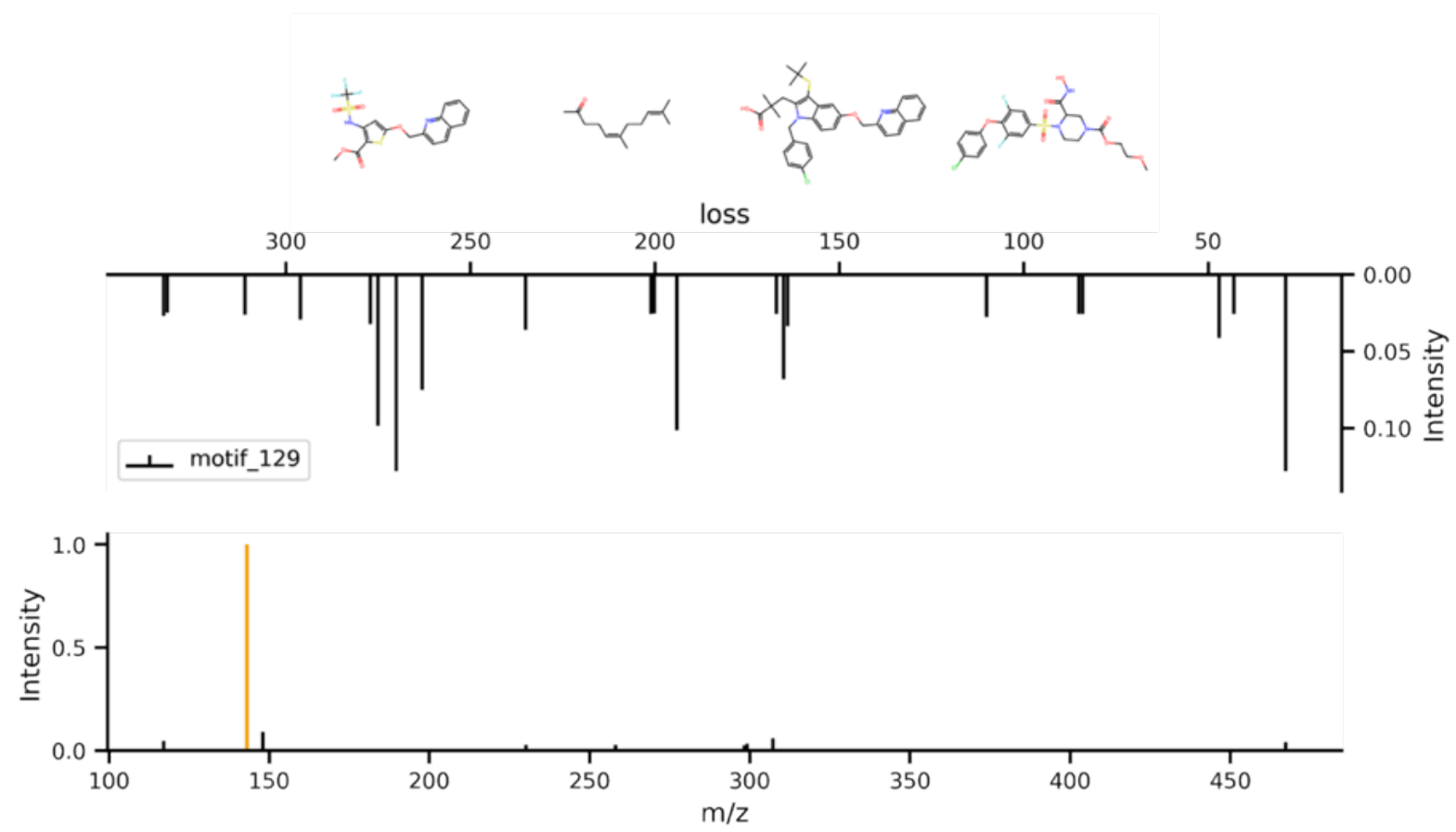

Fig. S31. Mass2Motif 129 is associated with bromotryptamine-like spectral features. The top panel shows the only structure from MAG. The bottom panel displays the fragments associated with this Mass2Motif as a standard MS/MS spectrum. The orange highlighted fragment is consistently observed in the recommended structure. Mirrored above the fragments are the corresponding neutral losses, arranged from right to left in increasing mass difference from the precursor ion.

Four examples where Mass2Motifs explain Suspect list candidates

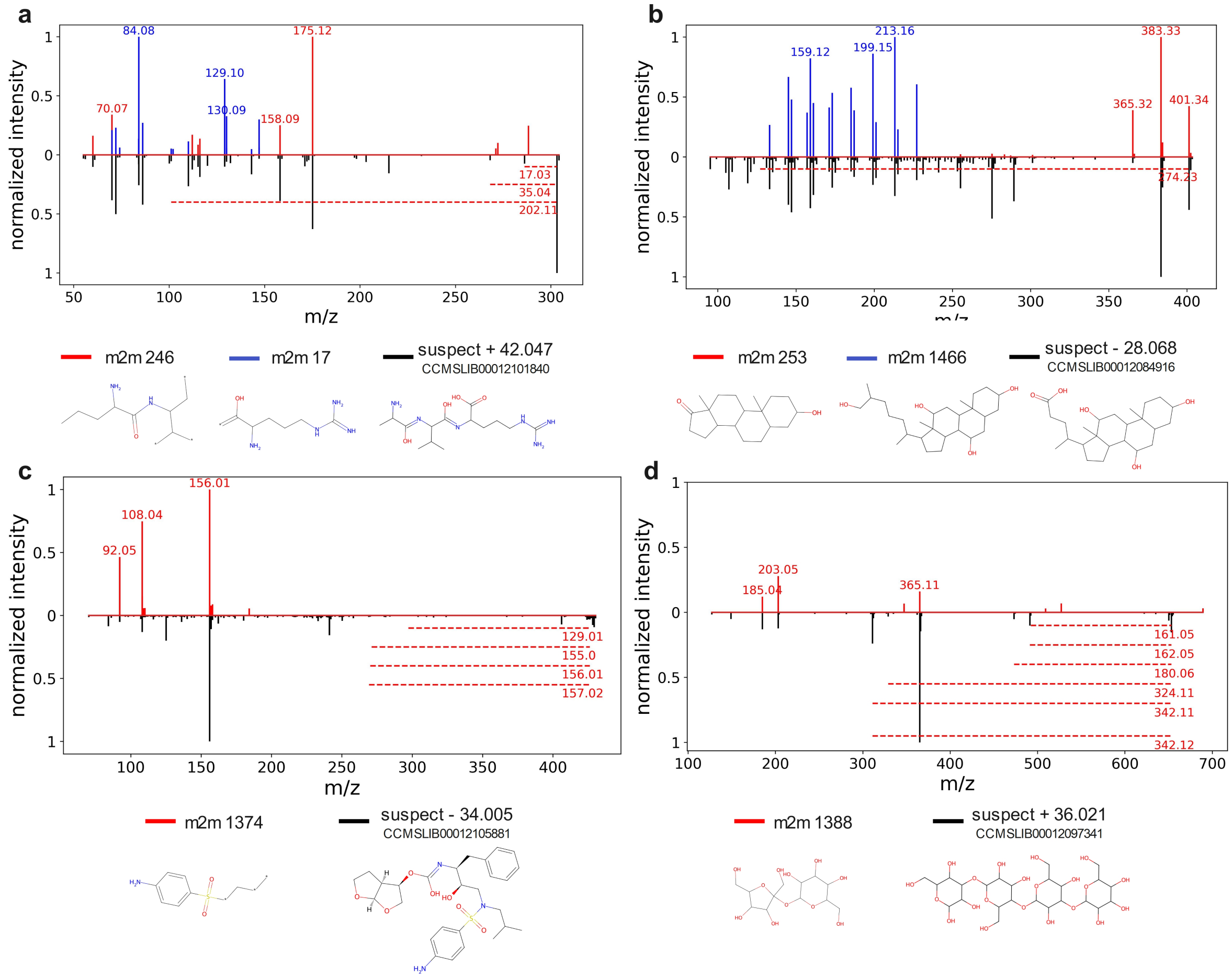

Fig. S32: Results from Mass2Motif exploration with the GNPS spectral suspect list. Four examples (a-d) show Mass2Motifs that represent substructures of identified suspects. The blue and red highlighted fragments represent the corresponding Mass2Motifs, while mirrored spectra in black represent the related suspect spectra. selected. The dotted horizontal lines represent losses belonging to the same colored Mass2Motifs. Below each mirror-plot, the MAG based recommendation (left, under the Mass2Motif (m2m) color identifier) as well as manual suspect reported by Bittremieux et. al. (right, under the suspect color indicator) are shown.

Mass2Motif annotation and probability for suspect candidate (S32 a)

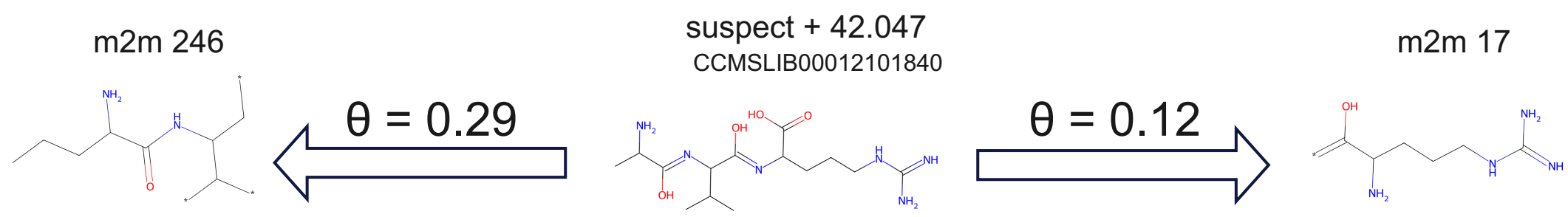

Fig. S33. Topic-document probability between Mass2Motifs 246 and 17 (left and right) and suspect (center). This shows how the Mass2Motifs represent different substructures of the complete suspect molecule.

Mass2Motif annotation and probability for suspect candidate (S32 b)

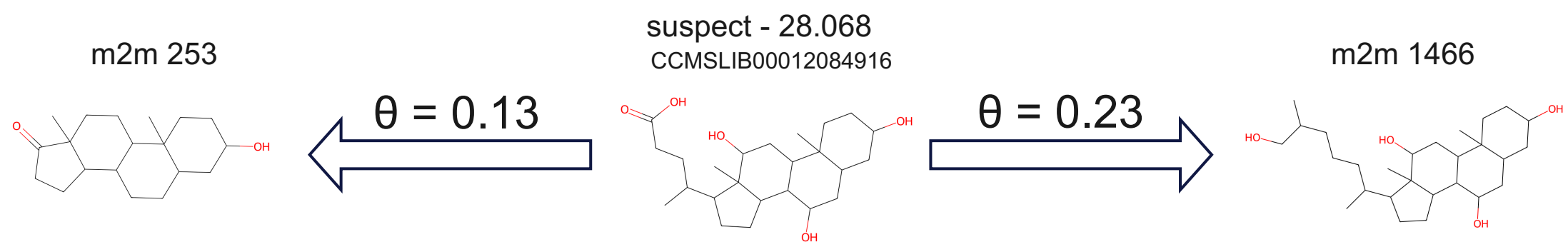

Fig. S34. Topic-document probability between Mass2Motifs 253 and 1466 (left and right) and suspect (center). This shows how the Mass2Motif 1466, which covers most of the suspect molecule, shows higher probability than Mass2Motif 253, which also contains a steroid substructure.

Mass2Motif annotation and probability for suspect candidate (S32 c)

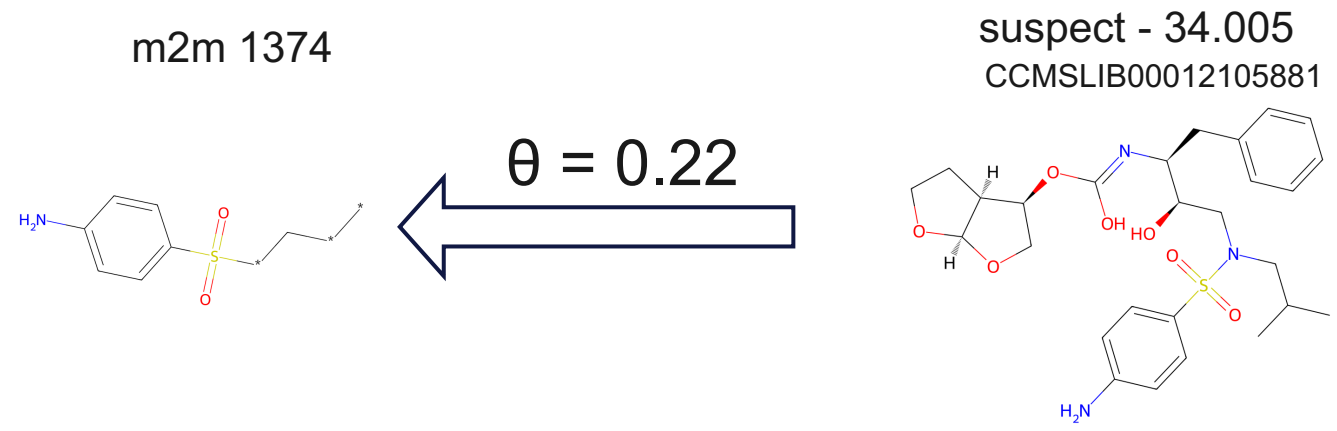

Fig. S35. Topic-document probability between Mass2Motifs 1374 (left) and suspect (right). This shows how the Mass2Motif 1374 represents part of the suspect structure with an aromatic ring attached to a sulfone functional group.

MassBank spectrum of darunavir to confirm fragment 108.04 belonging to sulfonamide moiety

Darunavir; LC-ESI-QFT; MS2; CE: 60; R=35000; [M+H]+

Names	Darunavir<br>N-[(1S,2R)-1-benzyl-2-hydroxy-3-[isobutyl(sulfanilyl)amino]propyl]carbamic acid [(3aS,4R,6aR)-2,3,3a,4,5,6a-hexahydrofuro[2,3-b]furan-4-yl] ester
Classes	N/A<br>Environmental Standard
SMILES	CC(C)CN(CC(C(CC1=CC=CC=C1)NC(=O)OC2COC3C2CCO3)O)S(=O)(=O)C4=CC=C(C=C4)N
InChI	InChI=1S/C27H37N3O7S/c1-18(2)15-30(38(33,34)21-10-8-20(28)9-11-21)16-24(31)23(14-19-6-4-3-5-7-19)29-27(32)37-25-17-36-26-22(25)12-13-35-26/h3-11,18,22-26,31H,12-17,28H2,1-2H3,(H,29,32)/t22-,23-,24+,25-,26+/m0/s1
SPLASH	splash10-0a4i-2900000000-98bfa9c688e4cb2dbbc3

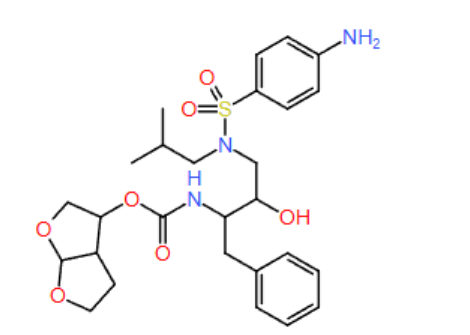

Formula: $C_{27}H_{37}N_3O_7S$ Mass: **547.23522**

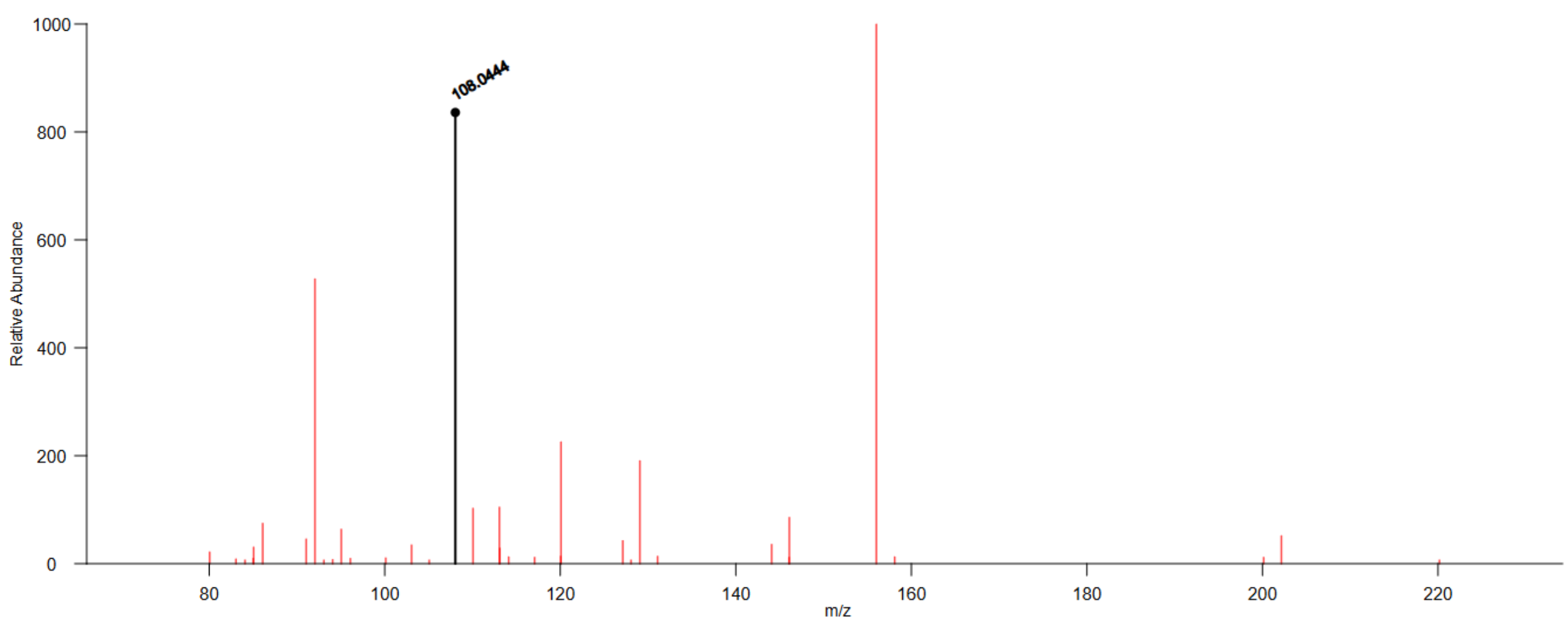

Fig. S36. Massbank spectra with fragment m/z 108.04 with a calculated molecular formula of C6H6NO+. The compound contains a sulfonamide moiety, which is based on the molecular formula responsible for the fragment 108.04.

Mass2Motif annotation and probability for suspect candidate (S32 d)

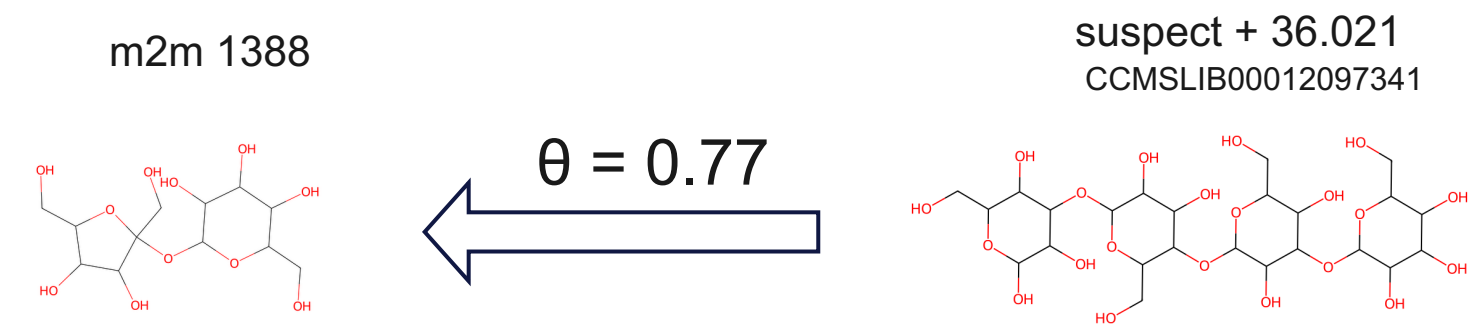

Fig. S37. Topic-document probability between Mass2Motifs 1388 (left) and suspect (right). This shows how the Mass2Motif 1388 a sugar moiety from the complete suspect molecule.

Comparison of speed between MS2LDA and MS2LDA 2.0 using a single core

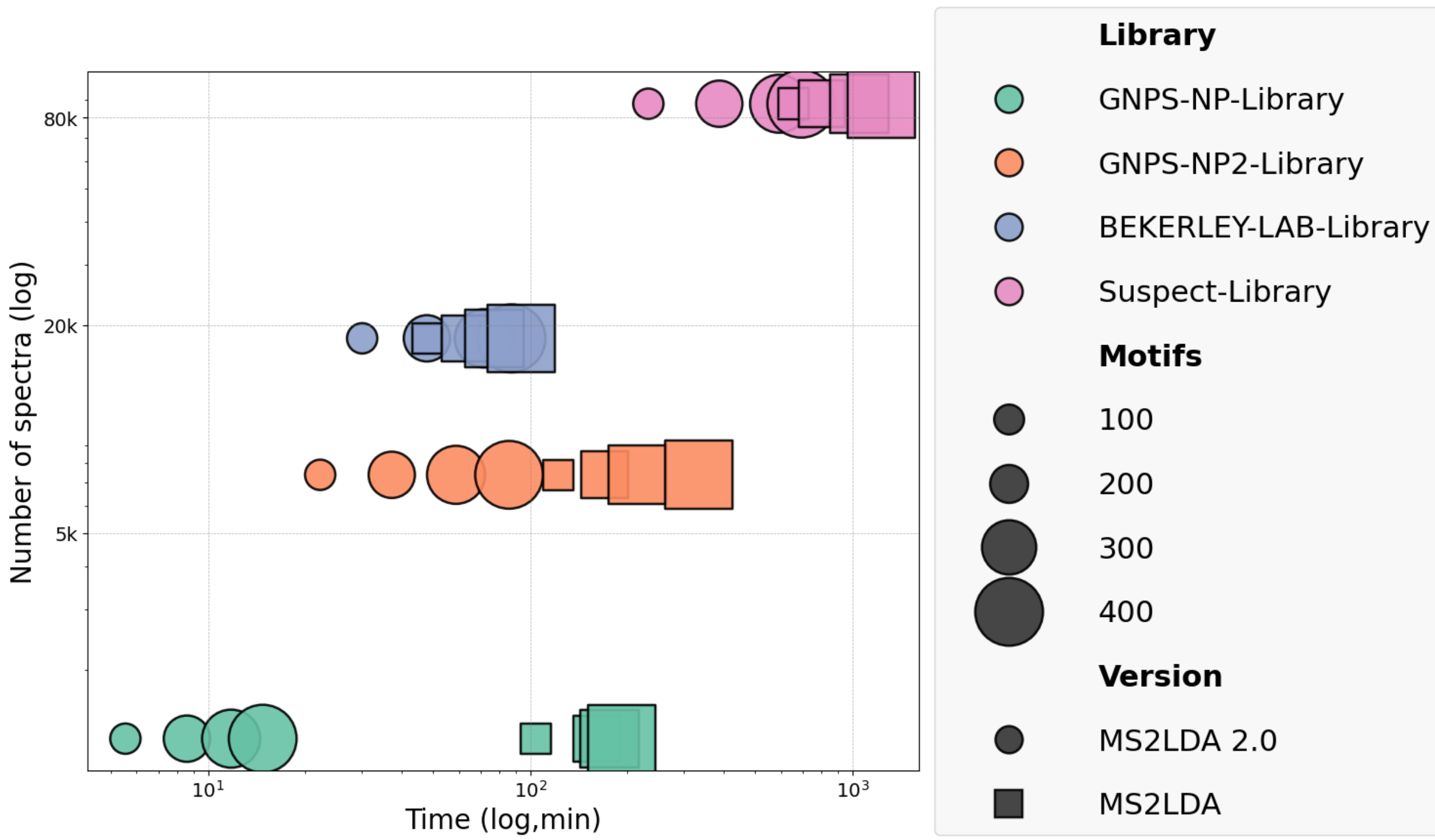

Fig. S38. Comparison of speed performance between MS2LDA and MS2LDA 2.0 across four different libraries: GNPS-NP (1,267 spectra), GNPS-NP2 (7,377 spectra), BERKELEY-LAB (18,352 spectra) and Suspect-List (87,899 spectra), using single core by AMD EPYC 7532 32-Core Processor. Running time is plotted on the x-axis and number of spectra on the y-axis. Software versions are distinguished by shape; number of motifs are represented by size and libraries are indicated by colour.

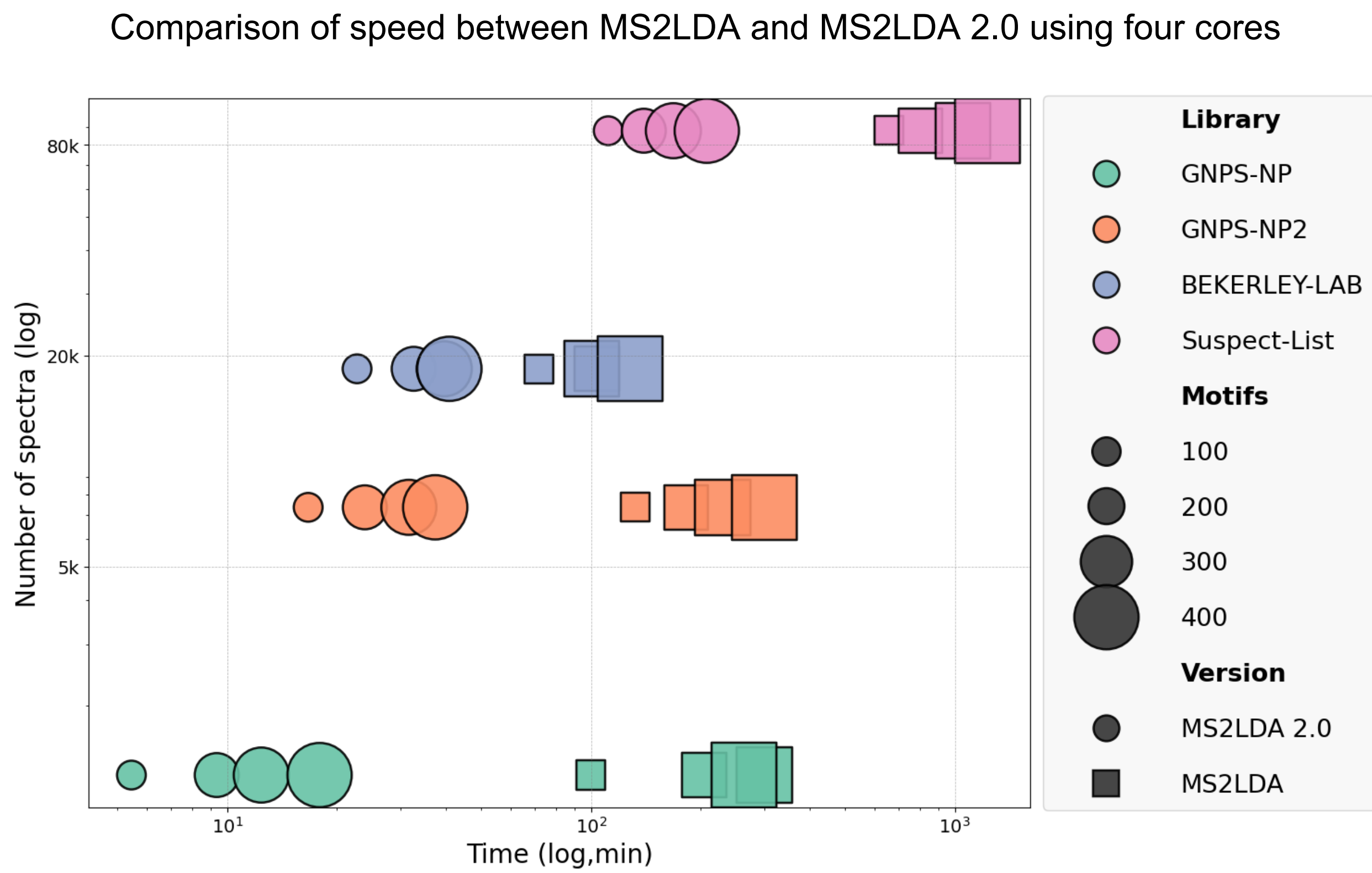

Fig. S39. Comparison of speed performance between MS2LDA and MS2LDA 2.0 across four different libraries: GNPS-NP (1,267 spectra), GNPS-NP2 (7,377 spectra), BERKELEY-LAB (18,352 spectra) and Suspect-List (87,899 spectra), using 4-cores by AMD EPYC 7532 32-Core Processor. Running time is plotted on the x-axis and number of spectra on the y-axis. Software versions are distinguished by shape, number of motifs are represented by size and libraries are indicated by colour.

MS2LDA 2.0 convergence curves for the pesticide case study

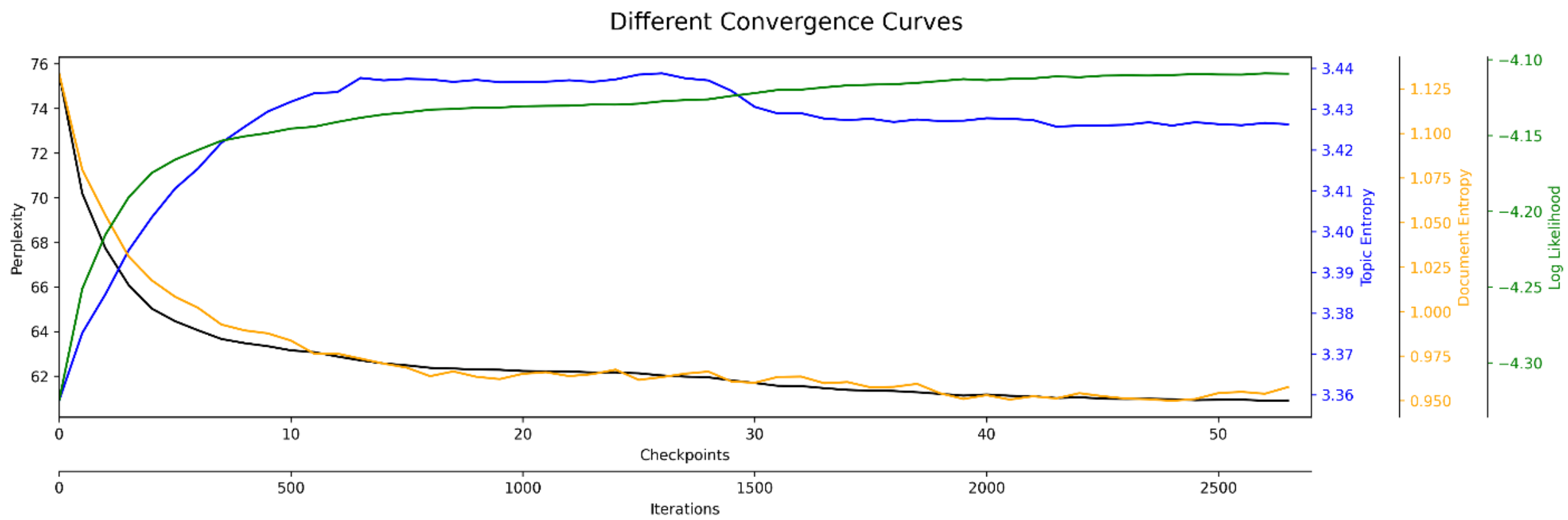

Fig. S40. Convergence curves that were obtained for the case study on pesticides. On the y-axis are plotted: Perplexity, topic entropy, document entropy and log likelihood. On the x-axis checkpoints and iterations (1 checkpoint = 50 iterations). This shows show the evolution of model performance metrics across iterations. Perplexity and document entropy decrease and stabilize after approximately 1800 iterations, indicating improved model fit and reduced uncertainty in document-topic assignments. In contrast, topic entropy and log-likelihood increase initially and likewise reach a plateau beyond 1800 iterations, reflecting improved topic diversity and overall model optimization. These trends collectively indicate stable convergence of the model.

MS2LDA 2.0 convergence curves for the edible mushroom case study

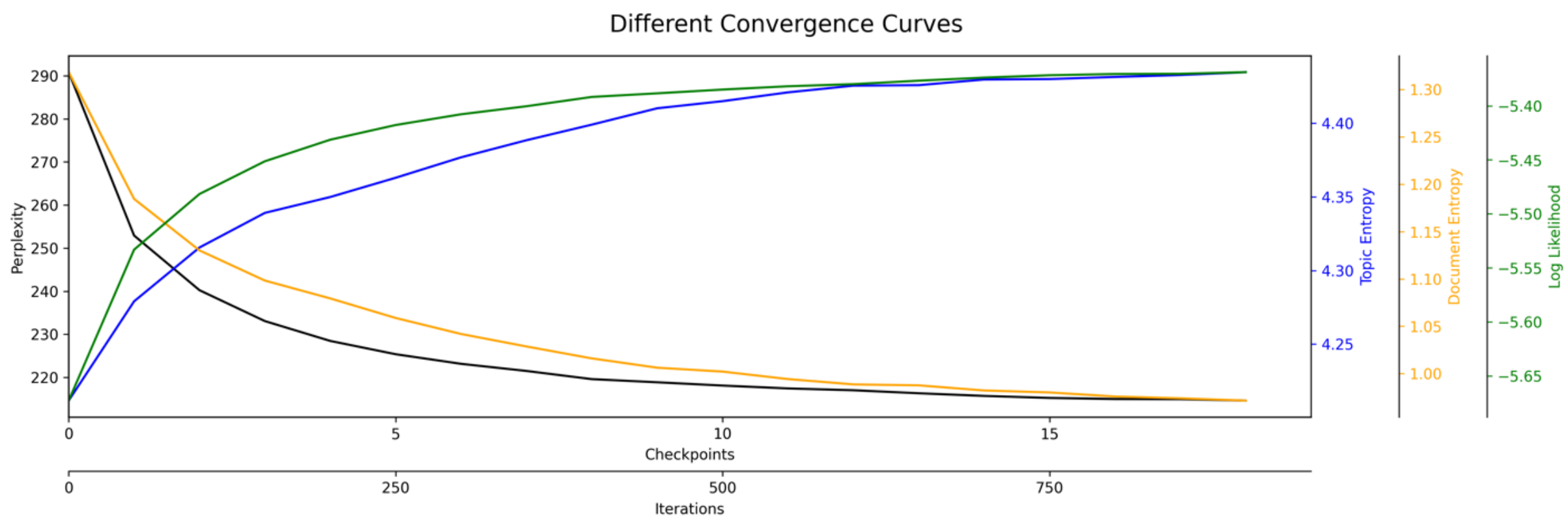

Fig. S41. Convergence curves that were obtained for the case study on edible mushrooms. On the y-axis are plotted: Perplexity, topic entropy, document entropy and log likelihood. On the x-axis checkpoints and iterations (1 checkpoint = 50 iterations). This shows show the evolution of model performance metrics across iterations. Perplexity and document entropy decrease and stabilize after approximately 750 iterations, indicating improved model fit and reduced uncertainty in document-topic assignments. In contrast, topic entropy and log-likelihood increase initially and likewise reach a plateau beyond 750 iterations, reflecting improved topic diversity and overall model optimization. These trends collectively indicate stable convergence of the model.

MS2LDA 2.0 convergence curves for the suspect list case study

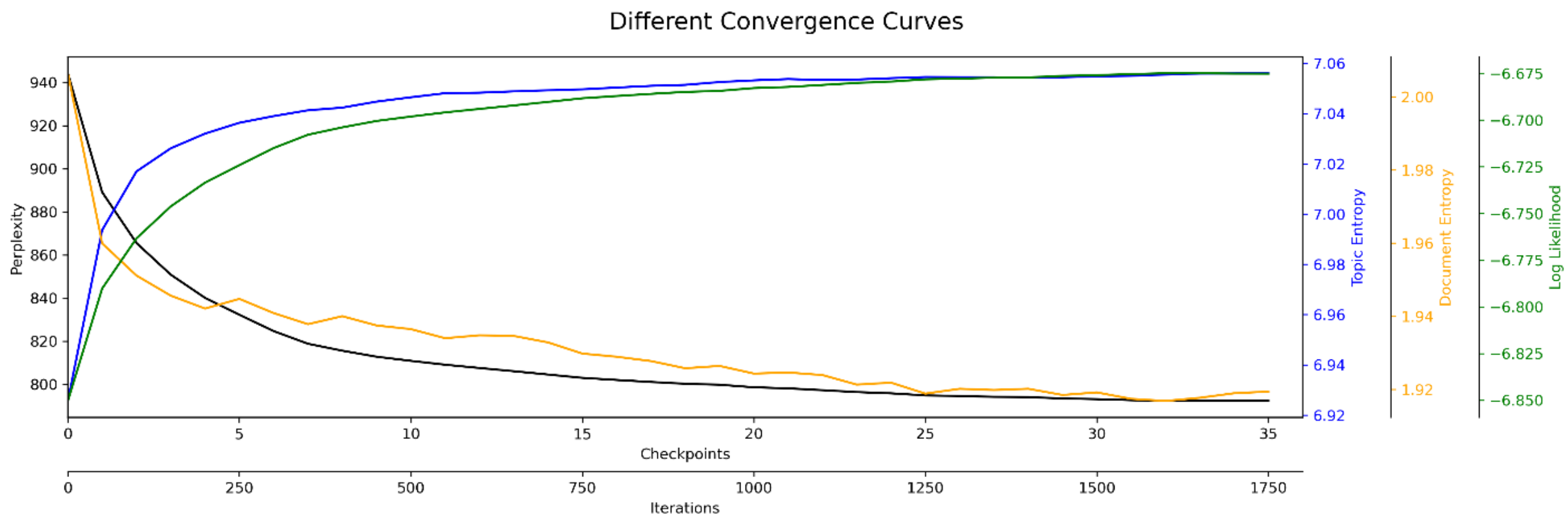

Fig. S42. Convergence curves that were obtained for the suspect list case study. On the y-axis are plotted: Perplexity, topic entropy, document entropy and log likelihood. On the x-axis checkpoints and iterations (1 checkpoint = 50 iterations). This shows show the evolution of model performance metrics across iterations. Perplexity and document entropy decrease and stabilize after approximately 1500 iterations, indicating improved model fit and reduced uncertainty in document-topic assignments. In contrast, topic entropy and log-likelihood increase initially and likewise reach a plateau beyond 1500 iterations, reflecting improved topic diversity and overall model optimization. These trends collectively indicate stable convergence of the model.

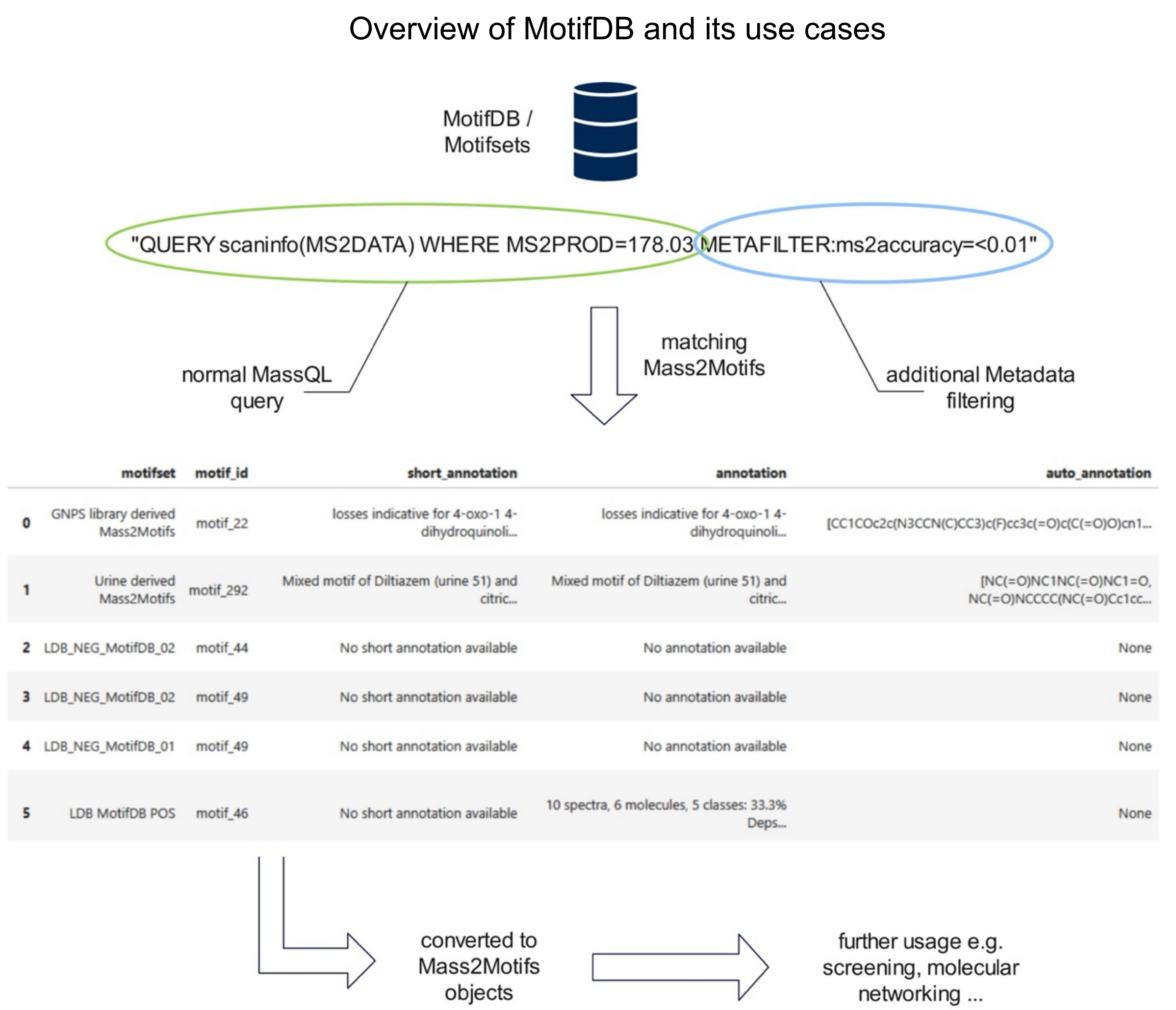

	motifset	motif_id	short_annotation	annotation	auto_annotation
0	GNPS library derived Mass2Motifs	motif_22	losses indicative for 4-oxo-1 4-dihydroquinoli...	losses indicative for 4-oxo-1 4-dihydroquinoli...	[CC1COc2c(N3CCN(C)CC3)c(F)cc3c(=O)c(C(=O)O)cn1...
1	Urine derived Mass2Motifs	motif_292	Mixed motif of Diltiazem (urine 51) and citric...	Mixed motif of Diltiazem (urine 51) and citric...	[NC(=O)NC1NC(=O)NC1=O, NC(=O)NCCCC(NC(=O)Cc1cc...
2	LDB_NEG_MotifDB_02	motif_44	No short annotation available	No annotation available	None
3	LDB_NEG_MotifDB_02	motif_49	No short annotation available	No annotation available	None
4	LDB_NEG_MotifDB_01	motif_49	No short annotation available	No annotation available	None
5	LDB MotifDB POS	motif_46	No short annotation available	10 spectra, 6 molecules, 5 classes: 33.3% Deps...	None

Fig. S43. Graphical MotifDB workflow starting by querying MotifDB via MassQL, then converting it to a Mass2Motif object that can be used for molecular networking or spectra screening or Mass2Motif matching tasks.

MS2LDA 2.0 interface: Start page with modeling settings

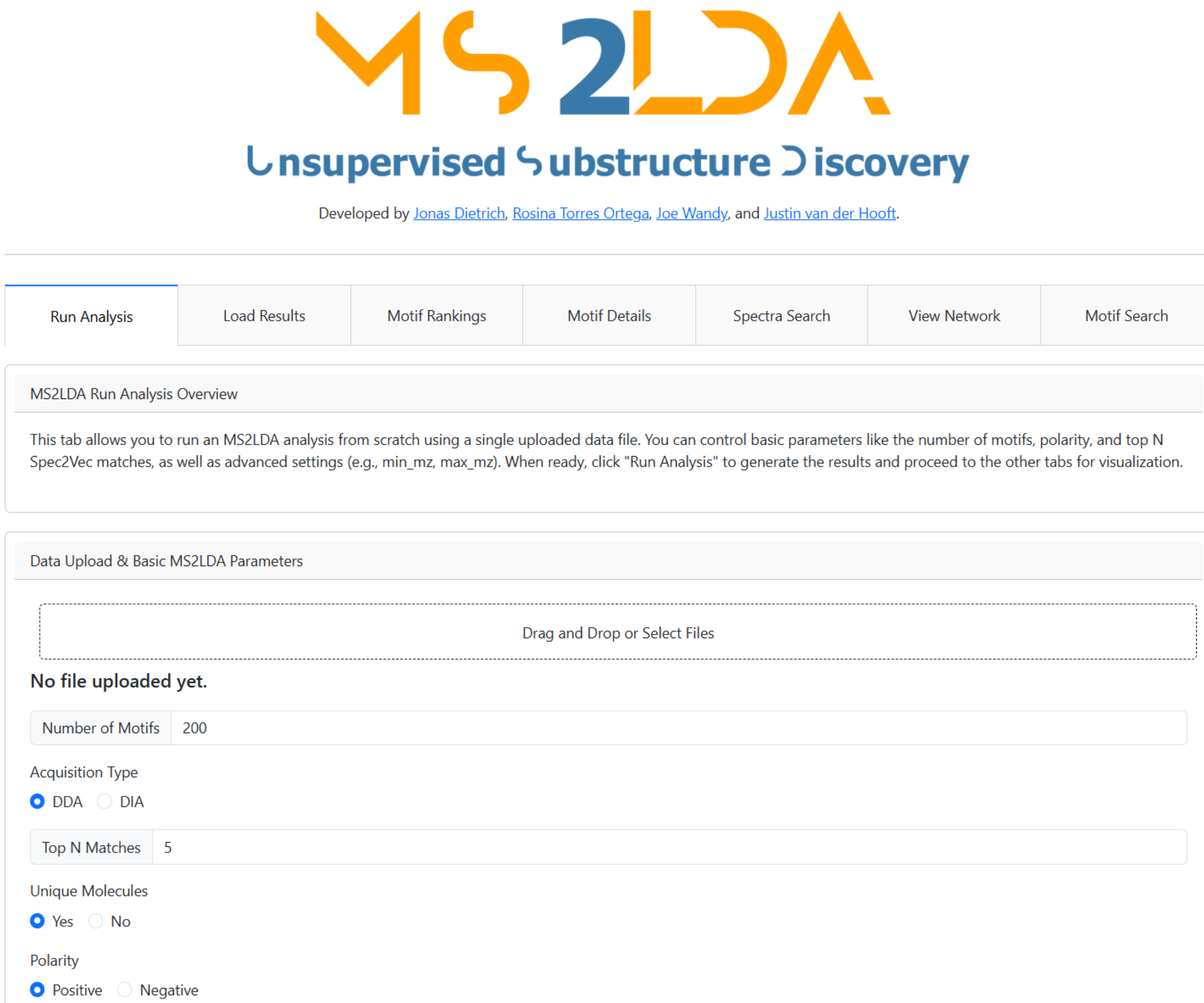

Fig. S44. Example of the landing page from MS2LDA 2.0 app. It starts with the tab "Run Analysis", then after running, the results can be submitted at the "Load Results" section, "Motif Rankins" serves to generally exploring all the Mass2Motifs inferred in an experiments, once you select a motif, you can further inspect it at the "Motif Details" tab, on the "Spectra Search" you can explore spectra containing certain motifs, "View Network" serves to explore the connections between our Mass2Motifs, if they share some fragments and "Motif Search" .

MS2LDA 2.0 interface: Motif Ranking tab

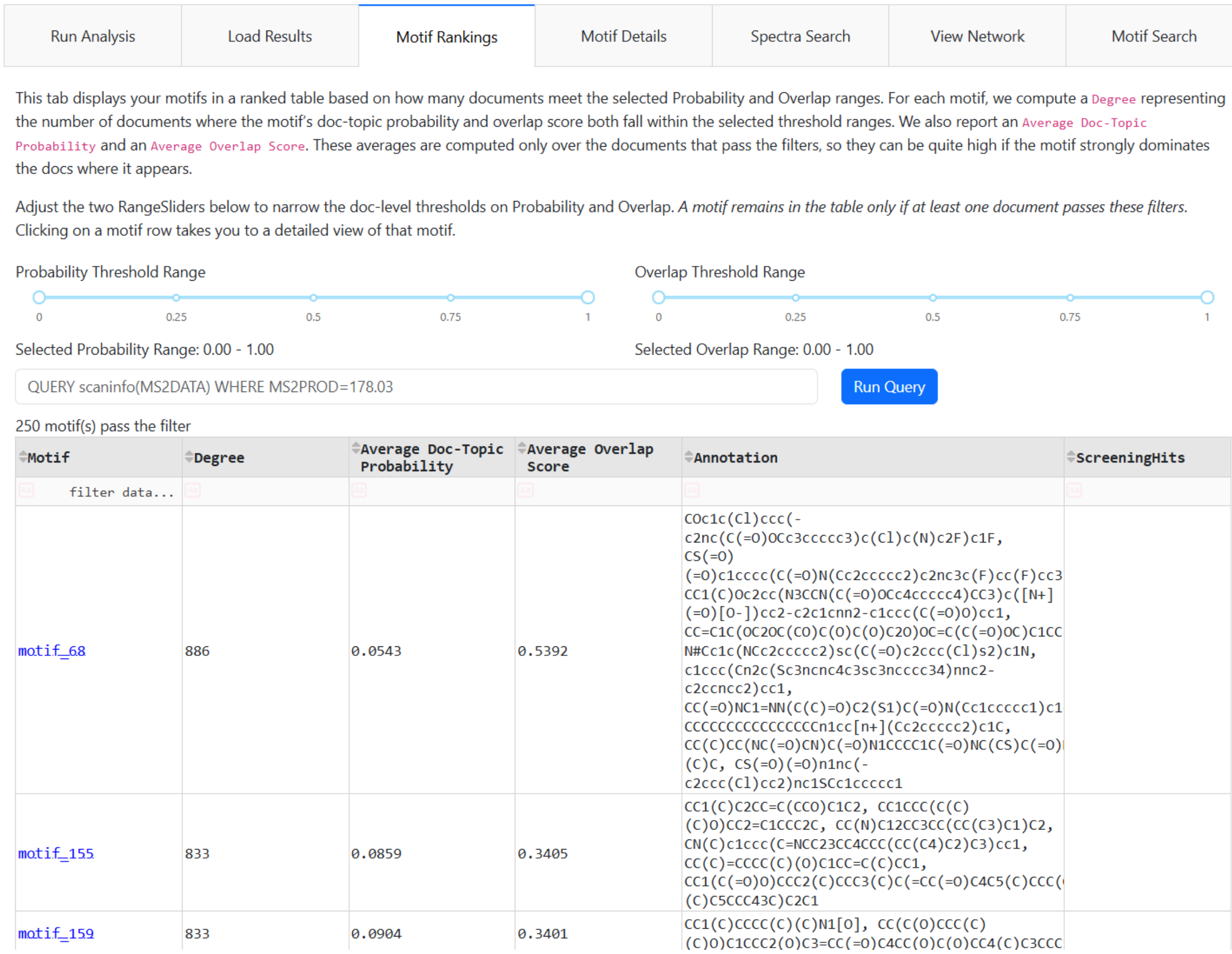

Fig. S45. Mass2Motif exploration interface in the "Motif Rankins" tab of the application. All Mass2Motifs are presented in an interactive table for exploration, accompanied by summary statistics to facilitate interpretation. The Degree indicates the number of MS/MS spectra in which given Mass2Motif occurs. The Average Doc-Topic Probability reflects how strongly a Mass2Motif is associated with each MS/MS spectrum on average, higher value meaning that a motif is commonly present in the data. The Average Doc-Topic Probabilities shows how much overlap there is among the fragment/neutral loss in the Mass2Motifs, with lower values indicating more distinct and interpretable motifs.

MS2LDA 2.0 interface: Motif Details tab

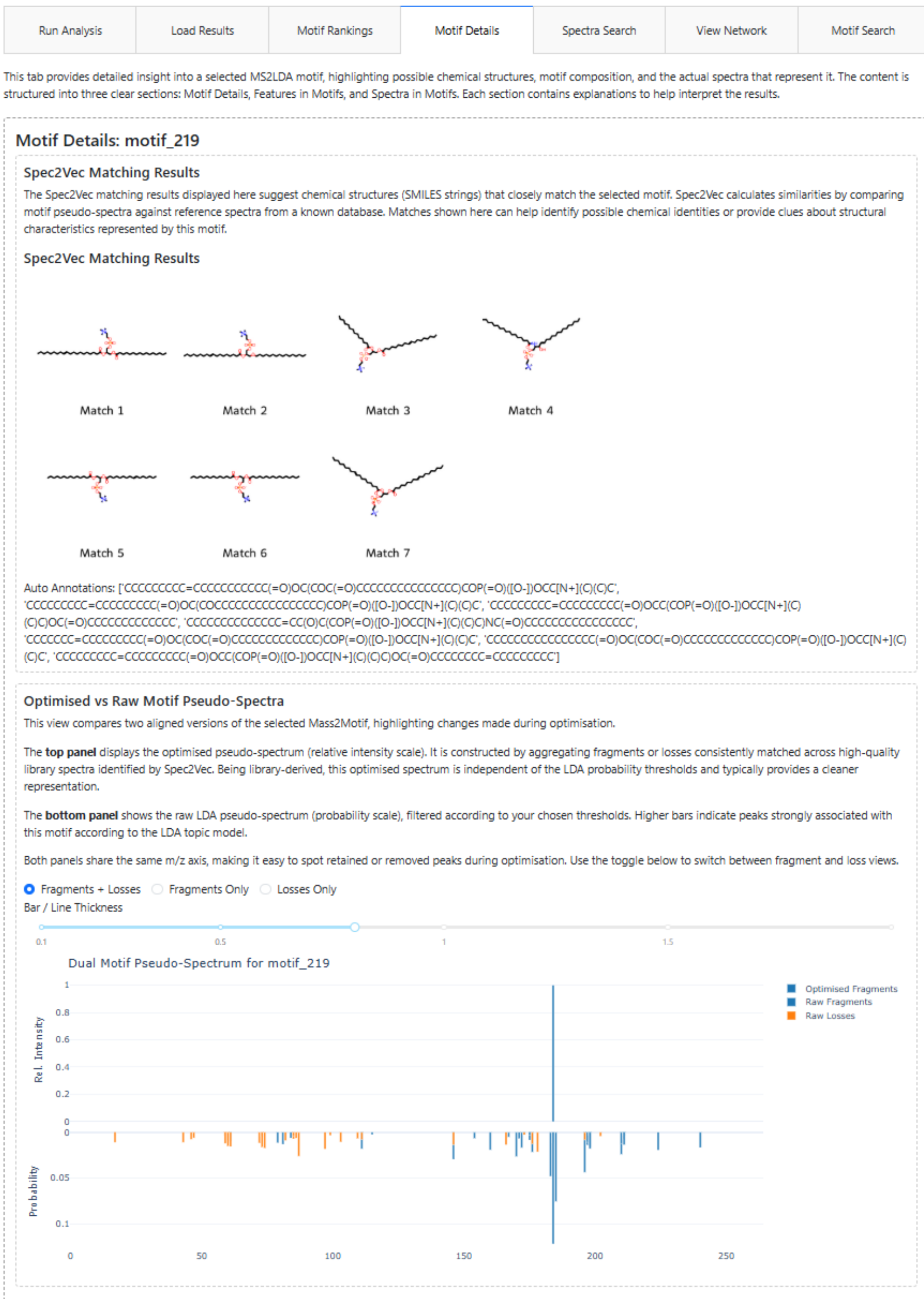

Fig. S46. Mass2Motif exploration interface in the "Motif Details" tab of the application. This section shows in detail the Mass2Motif selected. On top the recommended structures by MAG are shown. Below, a mirror spectrum shows on the bottom the Mass2Motif as a standard MS/MS with the fragments and losses and their corresponding probabilities on the x axis. On top it shows the optimized-spectra Mass2Motif, where fragments or losses shown are present in all the structural MAG recommendations given above with their relative intensity.

MS2LDA 2.0 interface: Motif Details tabs' spectrum-Mass2Motif comparison

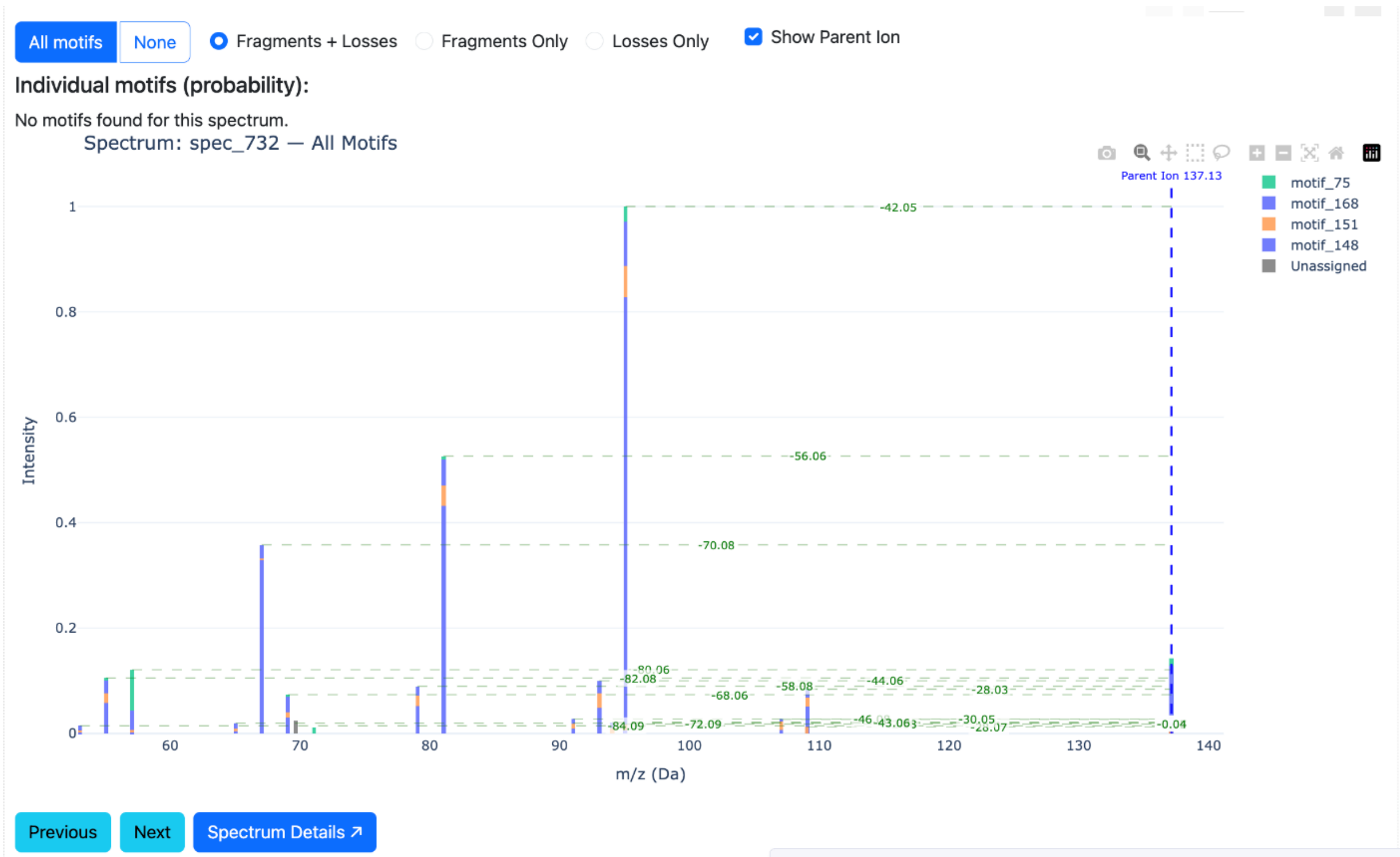

Fig. S47. Mass2Motif exploration interface in the "Motif Details" tab of the application. Below in this tab, MS/MS spectra can be explored with their assigned Mass2Motifs.

MS2LDA 2.0 interface: Spectra Search tab

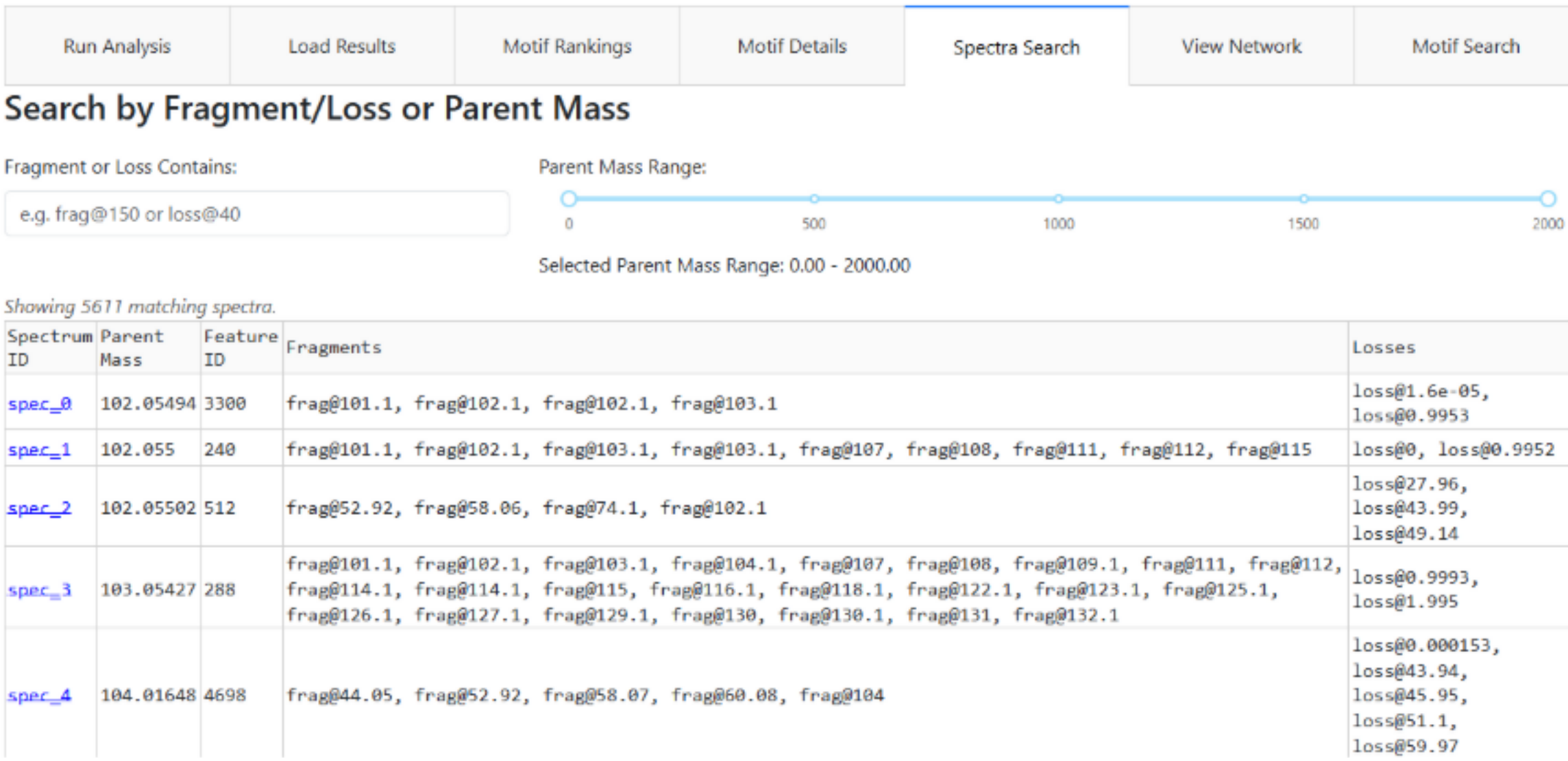

Fig. S48. "Spectra Search" section shown from the application. In this section, is possible to filtering spectra by parent mass range, by fragment and loss contained.

MS2LDA 2.0 interface: View Network tab

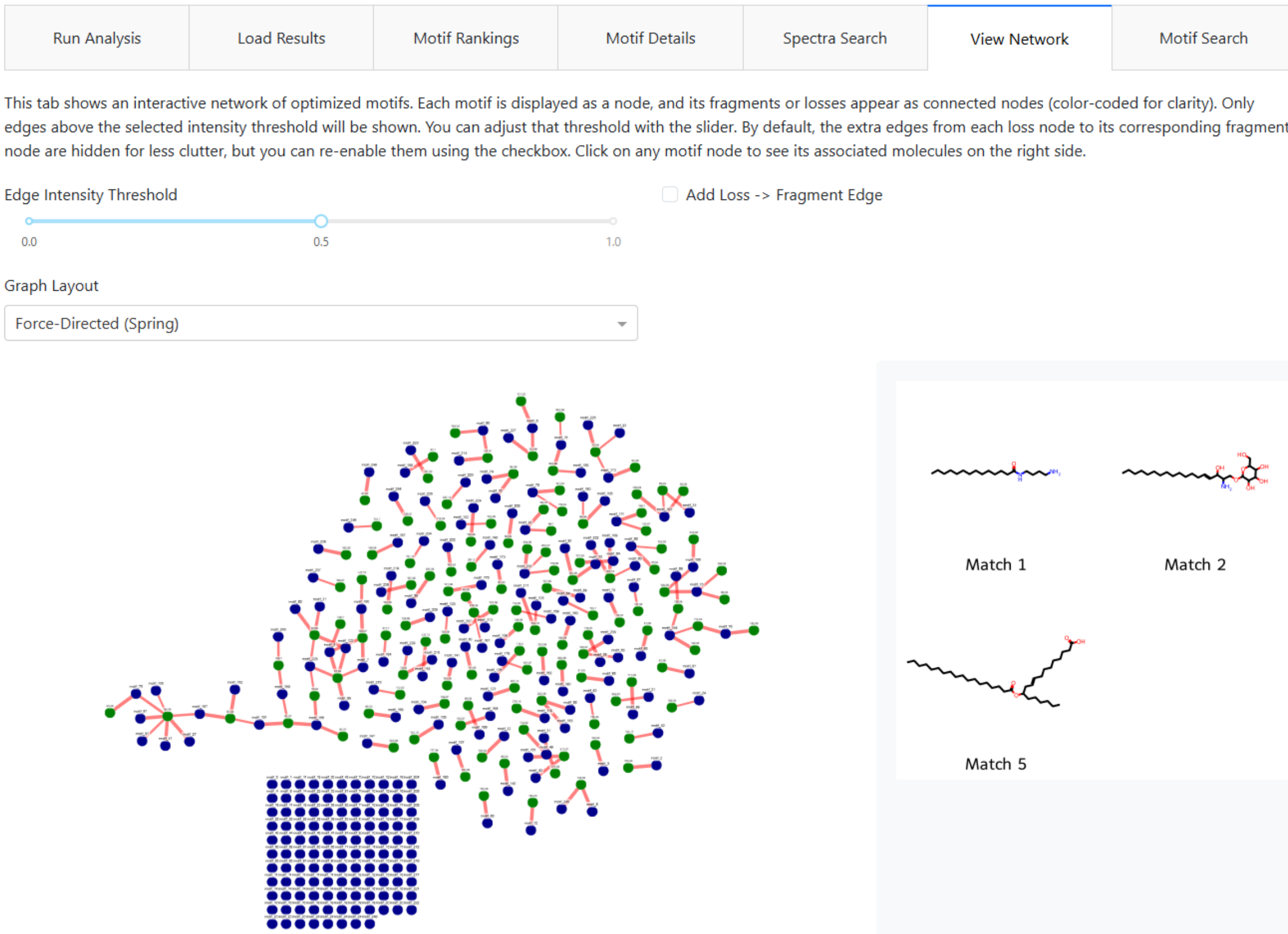

Fig. S49. "View Network" section shown from the application. This section allows to visualize the Mass2Motifs inferred using different layouts (Graph Layout), for example "Force-Directed". When a node is selected, the MAG recommendations are shown. The blue nodes represent the Mass2Motifs, green nodes represent fragments and yellow nodes represent losses. The network can be customized by changing the edge intensity threshold.

MS2LDA 2.0 interface: Motif Search tab

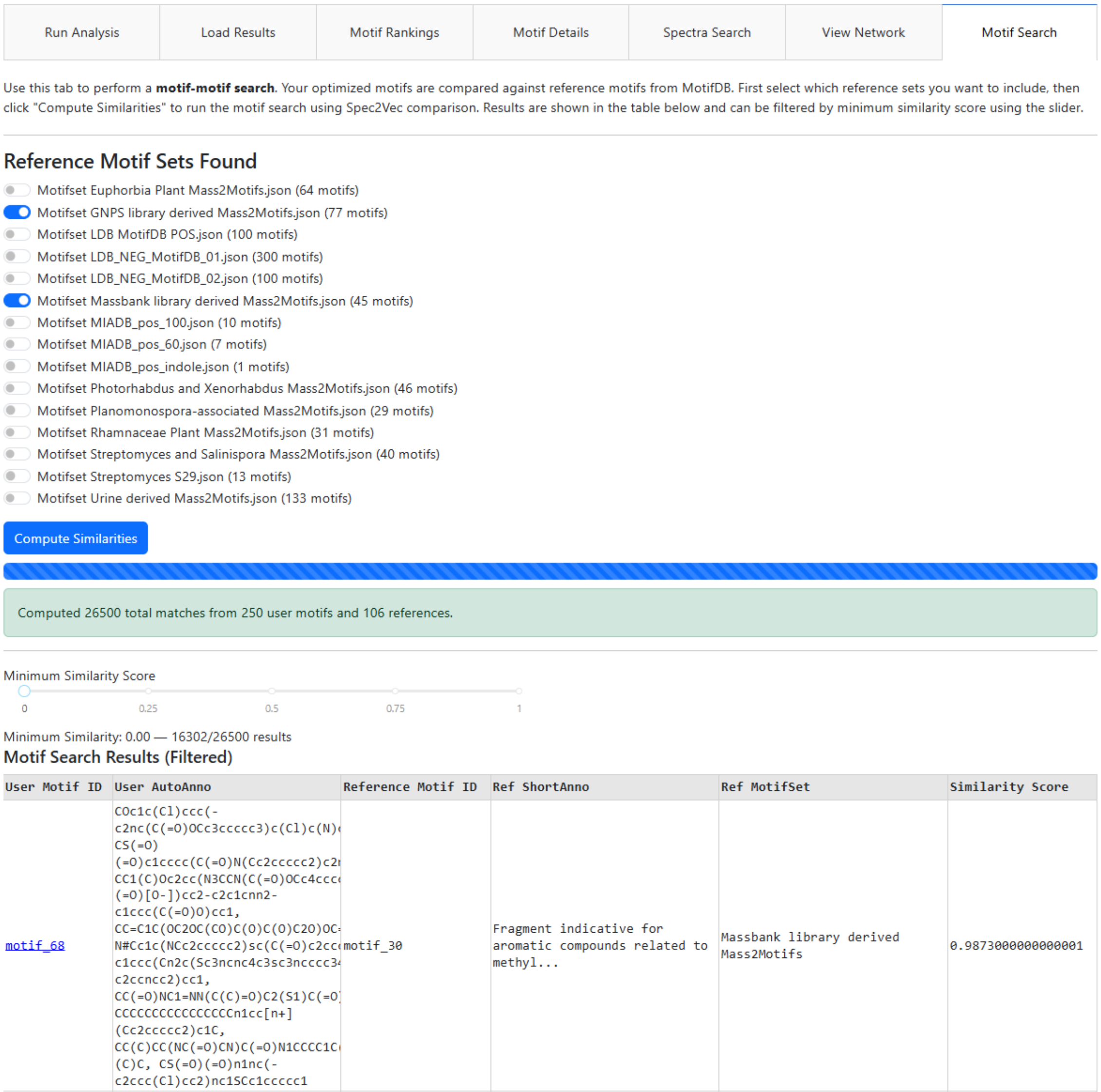

Fig. S50. "Motif Search" section shown from the application. This section enables to compute the Mass2Motif-matching, by computing Spec2Vec similarities between the inferred Mass2Motifs and MotifSets present in MotifDB. On top a selection of different MotifSets is given. After computing similarities, a table of results is shown. The results can be filtered using the Minimum Similarity Score sliding bar. In the results table, information about the Mass2Motif from the dataset and the Mass2Motif belonging to the MotifSet is shown plus their similarity score.

2. Supplementary Tables

Table S1. List of all 211 spiked pesticides.

Compound Name	Compound Name	Compound Name
2,4-D	Abamectin	Acephate
Acetamiprid	Aldicarb	Ametoctradin
Amidosulfuron	Asulam	Azamethiphos
Azoxystrobin	Bendiocarb	Bentazone
Benthiavalicarb-isopropyl	Benzovindiflupyr	Bifenazate
Bifenthrin	Bitertanol	Bixafen
Boscalid	Brodifacoum	Bromadiolone
Bromoxynil	Bromuconazole	Bupirimate
Buprofezin	Carbaryl	Carbendazim
Carbetamide	Carbofuran	Carfentrazone-ethyl
Chlorantraniliprole	Chlorbromuron	Chloridazon
Chlorpyrifos	Clodinafop-propargyl	Clofentezine
Clomazone	Clothianidin	Cyantraniliprole
Cyazofamid	Cybutryne	Cyflufenamid
Cyflumetofen	Cymoxanil	Cyproconazole
Cyprodinil	Cythioate	Cyfluthrin
Cyhalothrin	Cypermethrin	Deltamethrin
Desmedipham	Dichlofluanid	Difenoconazole
Diflubenzuron	Dimethenamid	Dimethoate
Dimethomorph	Dinoterb	Diuron
DNOC	Dodemorph	Dodine
Emamectin B1a	Epoxiconazole	Ethirimol
Ethoprophos	Etoxazole	Famoxadone
Fenamidone	Fenamiphos	Fenhexamid
Fenoxaprop-p-ethyl	Fenoxycarb	Fenpropidin
Fenpropimorph	Fenpyrazamine	Fipronil
Flonicamid	Florasulam	Fluazifop
Fluazinam	Flucycloxuron	Fludioxonil
Flufenacet	Flufenoxuron	Flumioxazin
Fluopicolide	Fluopyram	Fluoxastrobin
Flupyradifurone	Flupyrsulfuron-methyl	Fluroxypyr
Fluroxypyr-meptylester	Flutolanil	Fluxapyroxad
Foramsulfuron	Fosthiazate	Haloxyfop
Haloxyfop-P-methyl	Imazalil	Imazamox
Imidacloprid	Indoxacarb	Iodosulfuron-methyl
Ioxynil	Iprovalicarb	Isoproturon
Isopyrazam	Isoxaben	Isoxadifen-ethyl
Isoxaflutole	Kresoxim-methyl	Lenacil
Linuron	Malathion	Mandipropamid
MCPA	MCPP	Mepanipyrim
Mesosulfuron-methyl	Mesotrione	Metalaxyl
Metamitron	Metazachlor	Metconazole
Methabenzthiazuron	Methamidophos	Methiocarb
Methomyl	Methoxyfenozide	Metobromuron
Metolachlor	Metoxuron	Metrafenone

Metribuzin	Metsulfuron-methyl	Mevinphos
Myclobutanil	Napropamide	Nicosulfuron
Omethoate	Oxadixyl	Oxamyl
Oxathiapiprolin	Oxydemeton-methyl	Paclobutrazol
Penconazole	Pencycuron	Pendimethalin
Penflufen	Penthiopyrad	Phenmedipham
Picoxystrobin	Pinoxaden	Pirimicarb
Pirimiphos-methyl	Prochloraz	Profenofos
Propamocarb	Propiconazole	Propisochlor
Propyzamide	Prosulfocarb	Prosulfuron
Prothioconazole, desthio	Pymetrozine	Pyraclostrobin
Pyridaben	Pyridate	Pyrimethanil
Pyriproxyfen	Pyroquilon	Pyroxsulam
Quinmerac	Quinoclamine	Quinoxyfen
Quizalofop-ethyl	Rimsulfuron	Silthiofam
Simazine	Spinosyn A	Spinosyn D
Spirodiclofen	Spiromesifen	Spirotetramat
Spiroxamine	Sulcotrione	Tebuconazole
Tebufenpyrad	Tembotrione	Tepraloxydim
Terbuthylazine	Terbutryn	Tetraconazole
Thiabendazole	Thiacloprid	Thiamethoxam
Thiencarbazone-methyl	Thifensulfuron-methyl	Thiophanate-methyl
Tolclofos-methyl	Tolylfluanid	Topramezone
Triazophos	Tribenuron-methyl	Triclopyr
Trifloxystrobin	Triflumizole	Triflumuron
Triflusulfuron-methyl	Triforine	Trinexapac-ethyl
Tritosulfuron	Zoxamide	

Table S2. Probabilities between Mass2Motif and spiked pesticides for three different compound classes.

Compound Name	Mass2Motif	θ Probability
Organophosphates		
Omethoate	6	1.00
Dimethoate	6	0.99
Mevinphos	6	0.81
Cythioate	6	0.74
Oxydemeton-methyl	6	0.40
Tolclofos-methyl	6	0.32
Pirimiphos-methyl	6	0.02
Malathion	6	0.05
Benzoylureas		
Diflubenzuron	90	0.99
Flufenoxuron	90	0.46
Sulfonylureas		
Nicosulfuron	236	1.00

Foramsulfuron	236	0.98
Mesosulfuron-methyl	236	0.97
Flupyrsulfuron-methyl	236	0.62
Rimsulfuron	236	0.57
Amidosulfuron	236	0.10

Table S3. Results from the query Mass2Motif 137 against MotifDB.

motifset	motif_id	short_annotation	annotation
LDB MotifDB POS	motif_92	No short annotation available	15 spectra, 6 molecules, 3 classes: 66.7% Paraconic acids, 16.7% Acids, 16.7% Pulvinic Acid Derivatives
Euphorbia Plant Mass2Motifs	motif_333	Diterp 309 307 291 283 279	Diterp 309 307 291 283 279
Euphorbia Plant Mass2Motifs	motif_14	Diterp 611 333 277 275 259	Diterp 611 333 277 275 259
Urine derived Mass2Motifs	motif_59	Verapamil	Verapamil related (anithypertensive)

Table S4. Results from the query Mass2Motif 108 against MotifDB

motifset	motif_id	short_annotation	annotation
MIADB_pos_100	motif_19	Indole_in_mostly_corynanthean_series	Indole_in_mostly_corynanthean_series
MIADB_pos_100	motif_42	Vobasane-containing-bisindole alkaloids	Vobasane-containing-bisindole alkaloids
Urine derived Mass2Motifs	motif_122	Paracetamol mercapturate related (i.e. metabolites containing N-acetylcysteine adduct with paracetamol)	Paracetamol mercapturate related (i.e. metabolites containing N-acetylcysteine adduct with paracetamol)
MIADB_pos_100	motif_51	hydroxylated or methoxylated Indole_Type1__MIAskeleton	hydroxylated or methoxylated Indole_Type1__MIAskeleton
Urine derived Mass2Motifs	motif_177	Drug or xenobiotic - present in three urines and both sulphation and glucuronidation of core structure	Drug or xenobiotic - present in three urines and both sulphation and glucuronidation of core structure

Table S5. Results from the query Mass2Motif 186 against MotifDB.

motifset	motif_id	short_annotation	annotation
LDB MotifDB POS	motif_92	No short annotation available	15 spectra, 6 molecules, 3 classes: 66.7% Paraconic acids, 16.7% Acids, 16.7% Pulvinic Acid Derivatives
LDB MotifDB POS	motif_23	No short annotation available	6 spectra, 4 molecules, 3 classes: 50.0% Chromanes and Chromones, 25.0% Terpenoids :

			Triterpenes, 25.0% Depsides (Didepsides)
Euphorbia Plant Mass2Motifs	motif_333	Diterp 309 307 291 283 279	Diterp 309 307 291 283 279
LDB_NEG_MotifDB_02	motif_4	No short annotation available	No annotation available
Euphorbia Plant Mass2Motifs	motif_3	Loss of hexadienoic acid and its fragment / difference of 18 corresponds to the loss of-NH3 adduct	Loss of hexadienoic acid
Euphorbia Plant Mass2Motifs	motif_92	Diterp 297 279 269	Diterp 297 279 269
Euphorbia Plant Mass2Motifs	motif_14	Diterp 611 333 277 275 259	Diterp 611 333 277 275 259

Table S6. Results from the query Mass2Motif 90 against MotifDB.

motifset	motif_id	short_annotation	annotation
Urine derived Mass2Motifs	motif_29	Major loss is related to loss of sulphate group from phenolic (aromatic) structure (SO3 loss)	Major loss is related to loss of sulphate group from phenolic (aromatic) structure (SO3 loss)
GNPS library derived Mass2Motifs	motif_115	Sterone steroid related	Sterone steroid related Mass2Motif
Euphorbia Plant Mass2Motifs	motif_333	Diterp 309 307 291 283 279	Diterp 309 307 291 283 279
Urine derived Mass2Motifs	motif_22	Mixed motif of Quinidine (urine 97) and different cores in other urines	Mixed motif of Quinidine (urine 97) and different cores in other urines
LDB_NEG_MotifDB_02	motif_37	No short annotation available	No annotation available
Urine derived Mass2Motifs	motif_68	Alkyl aromatic substructure â€“ indicative for aromatic ring with 2-carbon alkyl chain attached i.e. phenylethene fragment	Alkyl aromatic substructure â€“ indicative for aromatic ring with 2-carbon alkyl chain attached i.e. phenylethene fragment
Urine derived Mass2Motifs	motif_106	unsat diC-FA	Unsaturated di-carboxilic fatty acids related Mass2Motif
Euphorbia Plant Mass2Motifs	motif_14	Diterp 611 333 277 275 259	Diterp 611 333 277 275 259

Table S7. Results from the query Mass2Motif 173 against MotifDB.

motifset	motif_id	short_annotation	annotation
Urine derived Mass2Motifs	motif_236	Mixed motif of modified Diltiazem related (urines 51 and 85) and smaller (methoxylated) phenolic substructure related Mass2Motif	Mixed motif of modified Diltiazem related (urines 51 and 85) and smaller (methoxylated) phenolic substructure related Mass2Motif
Rhamnaceae Plant Mass2Motifs	motif_3	-related motif	Myricetin-related motif

Table S8. Results from the query Mass2Motif 0 against MotifDB.

motifset	motif_id	short_annotation	annotation
LDB MotifDB POS	motif_16	No short annotation available	16 spectra, 9 molecules, 4 classes: 55.6% Depsidones, 22.2% Quinones, 11.1% Acids, 11.1% Paraconic acids
LDB MotifDB POS	motif_0	No short annotation available	4 spectra, 4 molecules, 3 classes: 50.0% Paraconic acids, 25.0% Xanthones and bis-Xanthones, 25.0% Depsidones
Urine derived Mass2Motifs	motif_291	Check - nitrogen containing ring core structure - could be from alkaloid?	Check - nitrogen containing ring core structure - could be from alkaloid?

Table S9. Description of MotifSets used for motif-matching for the medicinal mushroom dataset results

Motifset Name	Feature Set	Description	No. motifs
GNPS library	0.005 Da	MS/MS spectra obtained from reference compounds and isolated molecules from diverse sources with a focus on bacterial and plant related molecules - positive ionisation mode mass spectrometry on various instruments.	78
MassBank	0.005 Da	MS/MS spectra obtained from reference compounds and isolated molecules from diverse sources with a slight focus on plant related molecules - positive ionisation mode mass spectrometry on various instruments	46
LDB MotifDB POS	0.01 Da	MotifDB produced by the positive mode spectra of the LDB (250+ compounds with 745 spectra including different adducts and acquisition from three LC-MS instruments: Agilent 6530, Thermo Q-Exactive Focus, Waters Xevo G2-XS). Spectra were annotated with SMILES for MAGMa and substructure annotation. The chemical classes are from Huneck&Yoshimura (1996), and were used to annotate each motif. Parameters: 100 free motifs - binning of 0.01 Da	100

Table S10. Screening results from the App MS2LDA, of the mushroom dataset against GNPS, MassBank and LDB-positive MotifSets. The threshold was set as similarity above 0.7.

user_motif_id	user_auto_annotation	ref_motif_id	ref_short_annotation	ref_motif set	score
motif_10	COc1ccc(C(=O)c2ccccc2-c2ccc3c(c2)S(=O)(=O)Cc2cc(C(=O)NCc4ccc(C)cc4)sc2-3)cc1, O=C(O)CC(=O)O, CC(NC(=O)C(C#N)=Cc1cccc(Br)n1)c1ccccc1	motif_13	Alkyl aromatic substructure indicative for aromatic ring	Massbank library derived Mass2Motifs	0.7742
motif_10	COc1ccc(C(=O)c2ccccc2-c2ccc3c(c2)S(=O)(=O)Cc2cc(C(=O)NCc4ccc(C)cc4)sc2-3)cc1, O=C(O)CC(=O)O, CC(NC(=O)C(C#N)=Cc1cccc(Br)n1)c1ccccc1	motif_11	Alkyl aromatic substructure indicative for aromatic ring	GNPS library derived Mass2Motifs	0.7742
motif_156	CCC(=O)OC(CC(=O)[O-])C[N+](C)(C)C, CCC(=O)OC(CC(=O)O)C[N+](C)(C)C	motif_45	Fragments indicative of a glycosylation	GNPS library derived Mass2Motifs	0.7671
motif_156	CCC(=O)OC(CC(=O)[O-])C[N+](C)(C)C, CCC(=O)OC(CC(=O)O)C[N+](C)(C)C	motif_24	Fragments indicative of a glycosylation	Massbank library derived Mass2Motifs	0.7671
motif_126	NC(Cc1ccccc1)C(=O)O	motif_59	Fragments indicative for [phenylalanine-CHOOH] based substructure	GNPS library derived Mass2Motifs	0.7574000000000001
motif_126	NC(Cc1ccccc1)C(=O)O	motif_37	Fragments indicative for [phenylalanine-CHOOH] based substructure	Massbank library derived Mass2Motifs	0.7574000000000001
motif_61	CCCCCCCCCCCCOP(=O)(O)O, O=P(O)(O)OCCC(F)(F)C(F)(F)C(F)(F)C(F)(F)C(F)(F)C(F)(F)C(F)(F)C(F)(F)F, O=P(OC(CCl)CCl)(OC(CCl)CCl)OC(CCl)CCl	motif_48	Fragment ion indicative for conjugation of a phosphate group	GNPS library derived Mass2Motifs	0.7079000000000001
motif_61	CCCCCCCCCCCCOP(=O)(O)O, O=P(O)(O)OCCC(F)(F)C(F)(F)C(F)(F)C(F)(F)C(F)(F)C(F)(F)C(F)(F)C(F)(F)F, O=P(OC(CCl)CCl)(OC(CCl)CCl)OC(CCl)CCl	motif_26	Fragment ion indicative for the presence of	Massbank library derived Mass2Motifs	0.7079000000000001

			a phosphate group		
motif_148	CN(C)CCCCC(N)C(=O)O, NCCCCC(N)C(=O)O	motif_57	Fragment ions indicative for alkylamine substructure C5H10N	GNPS library derived Mass2Motifs	0.7067
motif_148	CN(C)CCCCC(N)C(=O)O, NCCCCC(N)C(=O)O	motif_35	Fragment ions indicative for alkylamine substructure C5H10N	Massbank library derived Mass2Motifs	0.7067

Table S11. CFM-ID 4.0 calculation for the peptide suspect with id = spec_20879. Used InChI for CFM-ID calculations: i= 'InChI=1S/C14H28N6O4/c1-7(2)10(20-11(21)8(3)15)12(22)19-9(13(23)24)5-4-6-18-14(16)17/h7-10H,4-6,15H2,1-3H3,(H,19,22)(H,20,21)(H,23,24)(H4,16,17,18) - 42.047 Da'

Frag or Loss	CFM-ID SMILES	Mass2Motif 17	Mass2Motif 246
Frag@60.06	NC(N)=[NH2+]	NO	YES
Frag@70.07	CC(C)C#[NH+]	YES	YES
Frag@72.08	CC(C)C=[NH2+]	YES	YES
Frag@143.12	C=C([NH3+])C(=O)NC C(C)C	YES	NO
Frag@175.12	NC(N)NCC=CC(=[NH2 +])C(O)O	NO	YES
Loss@17.03	NH3	NO	YES

Table S12. CFM-ID 4.0 calculation for the steroid suspect. Used InChI for CFM-ID calculations: InChI=1S/C24H40O5/c1-13(4-7-21(28)29)16-5-6-17-22-18(12-20(27)24(16,17)3)23(2)9-8-15(25)10-14(23)11-19(22)26/h13-20,22,25-27H,4-12H2,1-3H3,(H,28,29)/t13-,14+,15-,16-,17+,18+,19+,20+,22+,23+,24-/m1/s1

Frag or Loss	CFM-ID SMILES	Mass2Motif 1466	Mass2Motif 253
Frag@133.1	CCC(C)CCC(O)[OH2+]	YES	NO
Frag@145.1	C=CCC(C)CCC(O)[OH 2+]	YES	NO
Frag@147.12	CCCC(C)CCC(O)[OH2 +]	YES	NO
Frag@157.1	CC1CCC(O)CC1CC=[OH+]	YES	NO
Frag@159.12	CC1CCC(O)CC1CC[O H2+]	YES	NO
Frag@161.13	CC12C=CC=CC1=CC(=[OH+])CC2	YES	NO
Frag@185.13	CC12CCC(O)CC1CC([OH2+])CC2	YES	NO
Frag@187.15	CC1CCCC1C(C)CCC(O)[OH2+]	YES	NO

Frag@199.15	CC1CC([OH2+])CC2C C(O)CCC12C	YES	NO
Frag@213.16	CC(CCC(O)[OH2+])C1 =CC=CC1(C)CO	YES	NO
Frag@215.16	CC(CCC(O)[OH2+])C1 CC=CC1(C)CO	YES	NO
Frag@227.18	CC12CCC(O)CC1CC(O)CC2CC=[OH+]	YES	NO
Frag@365.32	CCC(C)C1CCC2C3C(O)CC4CC(O)CCC4(C) C3CC([OH2+])C12C	NO	YES

Table S13. CFM-ID 4.0 calculation for the sulfonamide suspect. Used InChI for CFM-ID calculations: InChI=1S/C27H37N3O7S/c1-18(2)15-30(38(33,34)21-10-8-20(28)9-11-21)16-24(31)23(14-19-6-4-3-5-7-19)29-27(32)37-25-17-36-26-22(25)12-13-35-26/h3-11,18,22-26,31H,12-17,28H2,1-2H3,(H,29,32)/t22-,23-,24+,25-,26+/m0/s1

Frag or Loss	CFM-ID SMILES	Mass2Motif 1374
Frag@92.05	C#CCC#CC=[NH2+]	YES
Frag@108.04	/	YES
Frag@156.01	[NH2+]=C1C=CC(=S(= O)=O)C=C1	YES

Table S14. Comparison between Mass2Motif objects and Matchms Spectrum objects. The main difference between Mass2Motif objects and matchms spectrum objects is that Mass2Motifs do not need fragments and losses can be stored within the object.

	MS2LDA 2.0 Mass2Motif object	Matchms Spectrum object
Contains fragments	Optional	Mandatory
Contains losses	Optional	No (calculated based on precursor mass)
Access fragments	.peaks.mz	.peaks.mz
Access losses	.losses.mz (retrieved)	.lossses.mz (calculated based on precursor mass)
Access metadata	.get(“metadata”)	.get(“metadata”)

Table S15. Parameter definitions and descriptions

Parameter type	Parameter	Type	Recommended Range	Default	Description
Preprocessing parameters					
	Min_mz	int	0-no limit	0	Minimum m/z value of fragments
	Max_mz	int	1000-2000	2000	Maximum m/z value of fragments
	Min_frags	int	2-10	5	Minimum number of fragments

	Max_frags	int	500-2000	1000	Maximum number of fragments
	Min_intensity	float	0.001-0.1	0.01	Minimum intensity of fragments
	Max_intensity	float	1	1	Maximium intensity of fragments
Convergence parameters					
	Step_size	int	50-200	50	Number that defines after how any iterations the convergence score is calculated
	Window_size	int	5-15	10	Range across which two consecutive convergence score should not change above the set threshold
	Threshold	float	0.01-0.001	0.005	Maximum allowed change for convergence score between two convergence scores across the window size
	Type	string	Perplexity_history, entropy_history_doc, entropy_history_topic, log_likelihood_history	Perplexity_ history	Type of convergence score
Annotation_ parameters					
	Criterium	string	Best or biggest	biggest	Returns the cluster with the most similar spectrum or the cluster with the most spectra
	Cosine_ similarity	float	0-1	0.9	For calculating the cosine distance for the agglomerative clustering (= 1 – Cosine_similarity). Therefore, number of clusters is set to None and the algorithm used to determine the clusters is hardcoded to “complete”
	N_mols_ retrieved	int	3-20	5	Number of most similar spectra retrieved from Spec2Vec database
N_motifs		int	Dataset type and size dependent; 50-200 as starting point	200	Number of generated Mass2Motifs

N_iterations		int	500-10000	10000	Number of iterations for LDA algorithm (can stop earlier when convergence criterium is reached)
Model_ parameter					
	Rm_top	int	The meaningless often occurring fragments the higher	0	Number of most occurring fragments that should be removed
	Min_cf	int	Higher for noisy spectra	0	Minimum number of occurrences for a fragment across the dataset
	Min_df	int	Higher for noisy spectra	3	Minimum number of occurrences for a fragment in a spectrum (based on intensity; intensity of 0.3 = 30 occurrences)
	Alpha	float	0.01-1	0.6	Dirichlet distribution for spectrum-Mass2Motif
	Eta	float	0.001-0.05	0.01	Dirichlet distribution for Mass2Motif-fragments
	Seed	int	User defined	42	Random seed (reproducibility)
Train_ parameters					
	Parallel	int	0-3	3	Parallelization of LDA modeling (see tomotopy documentation)
	Workers	int	0-number of cores available	0	Number of cores used (0 uses all available)
Dataset_ parameters					
	Acquisition _type	string	DDA or DIA	DDA	The difference is that DIA does not calculate any losses
	Significant _digits	int	1-3	2	Defines after how many numbers the fragments and losses are rounded
	Charge	int	-1,1	1	Descriptive value for positive (1) or negative (-1) mode
	Name	string	User defined	Ms2lda_ dashboa rd_run	Name of the motifset
	Output_folder	string	User defined	Ms2lda_ results	Name of the output folder

Fingerprint_ parameters					
	Fp_type	string	MACCS or RDKit	MACCS	Type of fingerprint used for motif fingerprint
	Threshold	float	0-1	0.8	Describes how often a bit in the fingerprint needs to be present across all MAG annotations to be part of the motif fingerprint (0.9 = 90%)
Motif_ parameter		int	10-50	50	Number of retrieved most important fragments and losses per Mass2Motif

Table S16: Comparison between the MassQL-modified version for MotifDB and the original MassQL format. The main difference is the storage of losses and metadata that can both be queried in the MotifDB version.

	MotifDB	MassQL
Contains fragments	Optional	Mandatory
Contains losses	Optional	No (calculated based on precursor mass)
Query MS1 data	No	Yes, massql syntax
Query MS2 data	Yes, massql syntax	Yes, massql syntax
Query metadata	Yes, metafiler:column=value	No

3. Supplementary Notes

3.1 Automated Mass2Motif Annotation Guidance and Benchmarking

3.1.1 The three steps of Mass2Motif Annotation Guidance

One of the core innovations of MS2LDA 2.0 is its ability to automatically guide the annotation of Mass2Motifs by suggesting structures that are likely to contain the detected substructure of interest.
The following paragraph describes the three steps that lead to a Mass2Motif Annotation Guidance (MAG): a library search with Spec2Vec[1], masking of fragments and losses, and finally a clustering of structural candidates based on the preceding steps. To illustrate MAG, we use a Mass2Motif from the case study on pesticides (Fig. S1).

The first step of MAG uses Spec2Vec to retrieve the most similar spectra between the Mass2Motif and a reference database containing over 500,000 spectra (see Methods section for details). The number of similar library spectra retrieved can be configured, with the default set to 10. If the task were to find full structural matches, Spec2Vec similarity scores above 0.7 or higher would be considered valuable and reliable information for an annotation. However, for substructure annotation, the scores are generally lower (often between 0.3 and 0.7) and less reliable. This reduced reliability also affects the relative ranking of spectra, meaning that a score of 0.62 may indicate a better substructure match than a score of 0.64.

The top ten spectra most similar to the Mass2Motif, based on Spec2Vec similarity, differ in terms of their base peaks (Fig. S2). To algorithmically detect such anomalies within the top ten spectra, a masking procedure is applied.
Masking is a procedure analogous to backward elimination: each fragment or loss feature is temporarily removed (masked), and the Spec2Vec similarity is recalculated. This approach helps localize the fragments and losses that contribute most to the structure matches and allows for clustering based on these important features. In our implementation, all fragments and losses are iteratively replaced with -1, and the Spec2Vec similarity between the masked Mass2Motif and each spectrum is recalculated. This results in a pattern suitable for clustering (Fig. S3).

Fig. S3 shows the result of agglomerative clustering, which identified three distinct clusters. The largest cluster (red) containing 7 compounds is recommended by MAG. Upon examining the structures behind the spectra, we see that MAG correctly clustered compound 2 (cluster 2), compounds 5 and 6 (cluster 3), and compounds 0, 1, 3, 4, 7, 8, and 9 (cluster 1), according to structural similarity (Fig. S4).

3.1.2 Mass2Motif Optimization

As shown in Fig. S4, Spec2Vec can sometimes suggest a group of chemical structures that do not share meaningful substructure similarities, for example, compound 1 and compound 2 in Fig. S4. This happens because the algorithm bases its suggestions solely on observed fragments and losses, which can be misleading.

MAG attempts to address this by clustering more similar compounds together. However, even within a single Mass2Motif, unrelated fragments or losses can still be present. These may result from noise or artifacts in the dataset, or from genuinely new substructures that are not included in any reference database. If such novel substructures are expected, manual inspection and annotation of the Mass2Motif becomes necessary.

In many cases, however, the problematic fragments are not truly novel but stem from misassignments made by the LDA algorithm—for instance, due to unique side chains or dataset-specific features. To refine the results, we remove all fragments that are not consistently found across the recommended group of structures (Fig. S5), only one fragment is common to compounds 0, 1, 3, 4, 7, 8, and 9—implying that the other fragments are likely unrelated to the core substructure shared by this group.

3.1.3 Substructure Overlap Score Definition

The first step in benchmarking MAG was to define a metric that quantifies how well a predicted substructure aligns with a manual annotated structure. We defined a substructure representation for each Mass2Motif using the Mass2Motif fingerprint (see Method section) and compared it to the fingerprint derived from manual annotations. The manual annotations were derived from three MotifSets in MotifDB: Urine-derived Mass2Motifs[2], GNPS[3] library Mass2Motifs, and Massbank[3] library-derived Mass2Motifs.
Since the Mass2Motif annotations in MotifDB are given as free-text descriptions, each description was manually converted to a SMILES representation. For example, the annotation “C7H7 and C5H5 fragments – indicative of methylbenzene substructure (aromatic)” was translated to the SMILES string “CC1=CC=CC=C1”. SMILES were retrieved from PubChem[4] using the compound names, and all mappings are documented in our GitHub repository (https://github.com/vdhooftcompmet/MS2LDA/tree/main/notebooks/Paper_results).
To compare manual and automatic annotations, we introduce the Substructure Overlap Score (SOS, formula 1). This metric quantifies how much of the smaller fingerprint is present in the larger one (with smaller, we refer to the number of 1s in a fingerprint and not the number of bits).

$$\text{SOS}=\frac{(fp_{smaller} \cap fp_{bigger})}{\sum_{b=0}^{B} fp_{smaller_b}} \qquad (1)$$

The decision to use always the smaller fingerprint instead of always using the manually annotated fingerprint as substructure was made since manual annotations in MotifDB do not consistently refer to well-defined substructures. For instance, annotations range from general substructures such as "phthalate substructure" to full compound-based

descriptions such as “(5-Hydroxy-2,2-dimethyl-4-oxo-3,4-dihydro-2H-chromen-7-yl)oxy substructure.”
In the main manuscript, we report SOS values based on MACCS fingerprints, as they align more closely with the information derived from mass spectrometry data. Unlike Daylight (created by RDKit[5]) fingerprints, which are sensitive to subtle differences in molecular topology, MACCS fingerprints are key-based and remain consistent across variations that would not produce different fragmentation patterns. However, MACCS fingerprints may also overestimate similarity by grouping together distinct structures that would yield different fragmentation spectra. Despite this, MACCS provides a pragmatic and arguably more realistic estimate of annotation accuracy than RDKit fingerprints alone. As shown in Fig. S6–S8, the differences between RDKit- and MACCS-based SOS scores are relatively minor.

3.1.4 Extended Benchmarks

This section presents benchmarks for three selected MotifSets. In addition to the results obtained using MACCS fingerprints, we also include results based on RDKit (Daylight) fingerprints. To validate the use of the SOS (Substructure Overlap Score) as a performance metric for MAG, we compared the MotifSet predictions with randomly selected compounds from the Spec2Vec reference database and processed them in the same way as for the Mass2Motif fingerprints.

Overall, the SOS score benchmarks demonstrate the improved performance of MAG compared to a random baseline (Fig. S6-S8). While randomly selected compounds from the reference database may occasionally show high structural overlap, primarily due to the presence of very common substructures, such matches are less abundant. In contrast, MAG consistently identifies structurally coherent matches, as reflected by significantly higher SOS scores in terms of distribution, mean, and average values. Through the comparison of randomly selected molecules as a baseline and the MAG annotation, we validated that MAG is able to suggest plausible structural matches for newly discovered Mass2Motifs with shared chemistry in mass spectral libraries.

3.2 Correlation between Mass2Motif features and annotation performance

To further evaluate MS2LDA 2.0 and especially MAG for substructure annotation, we described in the study that applying MS2LDA 2.0 on the MSn[6] library can give insights in the modelling and annotation aspect at the same time. While MACCS fingerprints are used in the study as a part of the SOS score using as a metric for MAG, in Fig. S9, we show results based on using Daylight RDKit fingerprints instead. In general, daylight fingerprints are more conservative since they do not allow isomers to match, which can follow the same fragmentation pathway. The general patterns seen with the MACCS fingerprints is also present in Fig. S9, but overall, the scores are lower, 79 Mass2Motifs have a high-quality annotation, 140 Mass2Motifs have an intermediate-quality annotation,

and 204 have a lower-quality annotation based on daylight fingerprints. The trend that more MAG annotations in combination with higher number of fragments and a larger maximum common substructure have a higher chance of being well annotated still holds.

3.3 Case Study Pesticides

3.3.1 Additional Mass2Motifs related to Pesticides

Beyond the Mass2Motifs highlighted in the case study on pesticide section in the main text, other pesticide-related Mass2Motifs could also be successfully identified.
For instance, the fragment at *m/z* 72.044 serves as a distinctive marker for dimethylated carbamates, which is a common substructure in many pesticides. Although this class is often defined by a single fragment, its presence provides a strong indication for flagging relevant compounds (Fig. S10).

Another prevalent pesticide group are the triazole pesticides. A specific triazole pesticide class is characterized by a triazole ring and a chlorinated benzene, which are captured by Mass2Motif 98 through the fragments at *m/z* 70.040 (triazole) and *m/z* 125.015 (chlorinated benzene). We note that, even though these substructures are not directly connected, MS2LDA 2.0 is able to recognize the co-occurrence pattern, demonstrating its capacity to associate distinct but recurring fragments (Fig. S11).

3.3.2 LDA probabilities for reported pesticides classes and their Mass2Motifs

For every spectrum in the input file for the MS2LDA 2.0 modelling, a probability score is calculated, which explains how much a spectrum is associated with a Mass2Motif. The scores are between 0 and 1, where 1 means a spectrum is only strongly associated with a Mass2Motif and 0 shows no association. In Table S2, the probability scores between three different Mass2Motifs representing organophosphates, benzoylureas, and sulfonylureas and their related spiked pesticides are shown.

The association between the compounds and Mass2Motifs highlighted by the probabilities reaches from 0.05 to 1.00 and was discussed in the case study on pesticides in the main text.

3.4 Case Study Natural Products

MS2LDA 2.0 identified common substructures from the molecular families described by Khatib et. al.[7]. We followed a strategy to dereplicate the Mass2Motifs present for each family: 1) we mapped the Mass2Motifs on the molecular families previously reported, 2) we explored the annotation of these motifs by MAG, 3) if a hit was retrieved, we queried the top three peaks present per Mass2Motif against MotifDB to explore its potential manual annotation using MassQL[8], and 4) we explore the results from the Mass2Motif-matching functionality against three MotifSets: GNPS, MassBank, and LDB MotifDB POS.

3.4.1 Erinacerin family findings

Finally, for the erinacerin family, mainly described in the main text, the manually annotated by Khatib et. al.[7], as erinacerin N with a precursor mass of 381.20 *m/z*, contains Mass2Motif 137 and Mass2Motif 22 (Figs S12 and S13, respectively), the MAG recommendation for Mass2Motif 137 identified as 4-[4-(2-chlorophenyl)piperazin-1-yl]-4-oxobut-2-enoic acid by MAG, being the pyridine substructure also part of erinacerin N. Some of the hits for this motif from MassQL results matched part of the structure (Table S7), such as motif_59, annotated as verapamil related, this has also a six-carbon ring as part of the molecule (3S)-2-[(1R)-1-[5-acetyl-2-(hydroxymethyl)-4-oxopyridin-1-yl]-2-methylbutyl]imino-3-hydroxy-4-methylpentanoic acid. For Mass2Motif 22, the MAG recommendation, strongly supports the same substructure reported by the original paper, although no hits were retrieved by MassQL. For the other compound manually annotated, erinacerin F, contains only Mass2Motif 108 (Fig. S22), we obtained five hits from MassQL (Table S8), most of which featured an indole-related structure, which aligns highly with the recommendation from MAG for this Mass2Motif.

For this family, both MAG and MassQL results aligned well with both compounds (erinacerin N and F) reported in the original paper. This is not always the case for every family reported in the paper. For example, the hericenone family contained redundant Mass2Motifs, where it shows the importance in hyper tuning parameters such as number of topics, alpha and beta. For this family, MassQL does not align very well with the MAG recommendation either. This could be due to differences in spectra quality.

3.4.2 Hericenone family findings

For the hericenone family we identified three main Mass2Motifs in its molecular family: Mass2Motif 163, Mass2Motif 186, and Mass2Motif 90, all nodes represented by at least one of them (Fig. S15). From the family reported by Khalib et. al.[7] the node with a precursor mass of 595.39 m/z, manually annotated as hericenone H, is represented by Mass2Motif 163 (Figure S16) and 90 (Figure S18). Hericenone G, with a precursor mass of 599.43 m/z, shared the same motifs. Finally, the manually annotated node with a precursor mass of 571.3986 *m/z*, is represented by Mass2Motif 186 (Fig. S17) and 90. All manually annotated nodes form the hericenone family shared Mass2Motif 90, which was annotated as a 2-(2-methylbut-2-enamido)acetic moiety by its first recommendation as a 4-fluoro-2-(4-hydroxyoxan-3-yl)-5-methyl-6-[(4-pyrazol-1-ylphenyl)methyl]-3H-isoindol-1-one by the second structural recommendation (Fig. S12, b). By inspecting these results, we can verify that the first recommendation 2-(2-methylbut-2-enamido) acetic, aligns more with the carbon chain side shown in all the hericenone molecules. On the other hand, both Mass2Motif 163 and Mass2Motifs 186 represent the same substructure, a benzodihydropyran, recommended by MAG, both substructures aligning well with the manually annotated molecules. These two motifs shared most of the peaks, that is the probable reason they retrieved the same structure.

All motifs were queried against all MotifDB entries. For Mass2Motif 163, we did not obtain any hits, even though their MAG recommendation is very similar to the Mass2Motif 186

recommendation, i.e. both share a two coumarin-like related structures. However, the top three peaks (by intensity) differ between the two Mass2Motifs, which could influence the retrieved hits obtained through MassQL. For Mass2Motif 186, MotifDB correctly retrieved terpenoid-like Mass2Motifs for its annotation (Table S5).

Finally, for Mass2Motif 90, the MassQL results are not conclusive, since it retrieves a wide variety of structures such as sterone related, phenols, diterpenes, etc. (Table S6). In this case, the discrepancy may be due to the presence of only one top peak (in red), which is the only peak present in the structure retrieved by MAG (Fig. S18).

The above findings demonstrate how the increased annotation capabilities through various routes in MS2LDA provide the user with additional sources for structure annotation guidance that return useful and relevant structural information on the experimentally discovered Mass2Motifs.

3.4.3 Ergostane family findings

We identified the top abundant Mass2Motifs present in the reported ergostane molecular family: Mass2Motif 173, Mass2Motif 0, Mass2Motif 21, and Mass2Motif 193. All nodes were represented by at least one of these motifs (Fig. S19). Mass2Motif 173 (Fig. S20) is present in two out of three manually annotated nodes, one corresponding to citreoanthrasteroide B, with a precursor mass of 393.315 *m/z* and ergosta-5,7,22,24(28)-tetraen-3beta-ol with a precursor mass of 395.33 *m/z,* its MAG recommendation corresponds highly with the original structures, with an steroid scaffold and a carbon side chain, as [17-(5,6-dimethylhept-3-en-2-yl)-4,4,10,13-tetramethyl-1,2,3,9,11,12,14,15,16,17-decahydrocyclopenta[a]phenanthren-3-yl] acetate. The MassQL retrievals (Table S7) pointed out Diltiazem or flavonoid motif like, not aligning with MAG annotations.

Ergosta-5,7,22,24(28)-tetraen-3beta-ol is also represented by Mass2Motif 21 (Fig. S21), which MAG recommendation points out a 2,15-dihydroxy-7-methyl-6-oxabicyclo[11.3.0]hexadeca-3,11-dien-5-one, which is not closely similar to the original structure proposed but they share the five-fused ring with the steroid backbone. We did not retrieve any results from MassQL-quering for this Mass2Motif.

For the last manually annotated compound, ergost-3,5,7,9(11),22-pentaen, it also contains Mass2Motif 21, and Mass2Motifs 0 and 193. For Mass2Motif 0 (Fig. S22), the MAG recommendation closely resembles the chain side carbon structure of this family reported previously (Fig. S19, b), the MassQL results retrieved a diverse class of motif-related structures such as depsidones, quinones and paraconic acids (Table S8). Mass2Motif 193 (Fig. 23) MAG recommendation features a sterol core structure without any side chain, a 5',7,9,13-tetramethylspiro[5-oxapentacyclo[10.8.0.02,9.04,8.013,18]icos-18-ene-6,2'-oxane]-16-ol. There were no retrieved Mass2Motifs from MassQL for this Mass2Motif.

These results show how MAG is a powerful assistant for Mass2Motif annotation, different Mass2Motifs may represent different part of a compound such as Mass2Motif 21 and

Mass2Motif 0 represent ergost-3,5,7,9(11),22-pentaen. Thus, analyzing the Mass2Motifs related to some compounds can help to reveal their structure.

3.4.4 Additional Mass2Motif annotations via motif-motif matching

In addition to the family compounds described in the original paper[7], we were able to annotate additional compounds present in the dataset. Using the Viz app from MS2LDA 2.0, we computed the similarity between the inferred Mass2Motifs and GNPS, MassBank and LDB MotifDB POS. These are the description of the MotifSets used (Table S9).

The Mass2Motifs with a cosine similarity above 0.7, are displayed in Table S10. Most of the MAG-recommended structures align closely with the manual annotations from MotifDB. For example, Mass2Motif 10 matched Mass2Motif 13 and 11 with scores of 0.77 from Massbank and GNPS MotifSets respectively, both annotated as alkyl aromatic substructure indicative for aromatic ring, which correlates with the MAG results shown in Fig. S24. Another good example was Mass2Motif 126 (Fig. S25), where the MAG recommendation correlates with the manual annotation of phenylalanine-like, with a high score of 0.757 against Mass2Motif 37 (from MassBank) and Mass2Motif 59 (from GNPS). Mass2Motif 61 is also noteworthy, as it is associated with a fragment ion indicative of the presence of a phosphate group. The MAG recommended structure clearly includes the phosphate group, as illustrated in Fig. S26. Not all the results completely align with SAG; for example, Mass2Motif 156 (Fig. S27). Although MAG recommendation does not exactly match with the manual annotation; further inspection reveals that one of the losses (161.1 m/z) could indicate the presence of a glycosylation (Fig. S28). This highlights the continued need for expert knowledge and curated annotation for some Mass2Motifs. It it's important to notice that all the Mass2Motif found using a threshold over 0.7 show the same annotation between MotifSet GNPS and MassBank, many motifs between these two sets overlap, since some of compounds and compound classes overlap between the spectral libraries.

3.5 Case Study on Accelerating Biological Discoveries with MAG-empowered MS2LDA

To investigate the impact of MAG on accelerating biological discoveries, we re-analyzed the previously reported siderophore degradation case study[9], and we investigated if Mass2Motifs related to Amphibactin F-like and bromotryptamine-like structures would be produced and annotated by MS2LDA 2.0. Besides reproducing previously found Mass2Motifs, we were especially interested in their MAG annotation as this is one of the key enhancements of the new version. For the first example, Amphibactin F and its degradation product shared a manually annotated motif in the original paper. From our results, these two metabolic features were found to be connected to motif_66. (see Fig. S29). The Mass2Motif optimization highlights the common fragments of *m/z* 86.06 and *m/z* 131.08 (in orange in Fig. S29. Interestingly, the three recommended structures also

contain the manually annotated N-acetyl-N-hydroxy-ornithine substructure, showcasing the acceleration in substructure discovery through MAG by saving time on manual Mass2Motif optimization and annotation through human expert knowledge.

In case of the other example, bromotryptamine-molecules (bromotryptamine, N-formyl bromotryptamine and N-acetyl bromotryptamine) motifs were not further explored in the original study. In our MS2LDA run, we found motif_34 (Fig. S30) and motif_129 (Fig. S31) were associated with these three features, showing how this motif can represent a unique substructure. However, the MAG annotation for both motifs returned unrelated (sub)structures. There is only one optimized fragment in both cases that represents all the recommended structures. In mass spectra data, many kinds of structures can yield a single similar fragment, so here MAG cannot give a lead for substructure annotation. This highlights the need of multiple optimized fragments for a higher chance of obtaining a good annotation (as in the previous example). Other potential reasons for an inconsistent annotation given by MAG are the poor coverage of bromotryptamine like compounds covered in the database or a missing and dominant fragmentation pattern for a substructure. Nevertheless, the MS2LDA 2.0 Mass2Motifs can connect structurally related molecules. Using tools like MassQL, the initial MS2LDA-inferred mass spectral patterns could be further refined, also using human expert knowledge.

3.6 Suspect list

In the case study on the suspect list, four examples were shown where at least one Mass2Motif was linked to an identified suspect, or more precisely to a substructure of the identified suspect. The information provided in 5.1-5.4 includes the topic-document probabilities, CFM-ID[10] explanations, and MassBank reference spectra to confirm the findings and links between Mass2Motifs, the MAG, and manual identified suspects.

In Fig. S32, a suspect that is structurally similar to alanyl-valyl-arginine could be associated with two Mass2Motifs that represent two different building blocks. Mass2Motif 246 is associated to the tris-amino substructure through the fragments m/z 60.06 and 175.12. Mass2Motif 246 and Mass2Motif 17 overlap with m/z 70.07 and 72.08 describing an alkylamine substructure. Furthermore, a loss of 17.03 in Mass2Motif 246 indicates the presence of a free amino group as it is present in the tris-amino substructure. Mass2Motif 17 mainly focuses on the amide substructure of arginine through fragment m/z 143.12. Mass2Motif 246 was the most dominant feature in 126 spectra, and Mass2Motif 17 in 311 spectra.

Fig. S32 b shows two Mass2Motifs that together describe a bile-acid related suspect. Mass2Motif 1466 focuses on the decomposition of the core steroid structure following the fragmentation patterns of androstane or metenolone alike structures41. Mass2Motif 253 is mainly defined by three fragments with m/z values above 350 (365.32, 383.33, 401.34), which have a m/z difference of 18.01, with 365.32 being annotated as a bile-acid core structure by CFM-ID 4.0. Therefore, these fragments probably describe a fragmentation pathway within the bile-acid conjugation side or at the core steroid structure losing two

water molecules. Mass2Motif 253 was dominant in 63 spectra and Mass2Motif 1466 in 481.

In Fig. S32 c, Mass2Motif 1374 describes a sulfonamide substructure, which is a building block of a suspect associated with Darunavir. The main fragments describe the fragmentation of the sulfonamide substructure from the suspect (m/z 156.01) and the further decomposition of the sulfonamide (m/z 108.04 and m/z 92.05). Mass2Motif 1374 was dominant in 96 spectra.

Fig. S32 d shows a suspect associated with 3-alpha,4-beta,3-alpha-galactotetraose, supported by at least two fragments with relatively high abundance. The difference between m/z 185.04 and m/z 203.05 indicates a loss of water, while additionally, several losses including 162.05 (hexose) further highlight how this Mass2Motif is related to the presence of conjugated sugar moieties including hexoses like glucose or galactose. Mass2Motif 1388 was dominant in 90 spectra.

3.6.1 Peptide example

Fig. S33 shows two peptide-related Mass2Motifs with probabilities of 0.12 and 0.29 being linked to a suspect peptide. The two Mass2Motifs shown had the highest probability of being associated with the suspect structure. Table S11 shows the fragments and losses of each motif and its CFM-ID annotation as a SMILES to confirm the MAG predictions.

3.6.2 Steroid example

Fig. S34 shows to Mass2Motifs being related with probabilities of 0.13 and 0.23 with the steroid suspect, which represent the two most associated Mass2Motifs with the suspect structure. Table S12 explains the fragments of the Mass2Motifs by CFM-ID annotations, which can confirm the MAG substructure prediction.

3.6.3. Sulfonamide example

Fig. S35 show one Mass2Motif representing a core substructure of the sulfonamide suspect. The probability of the Mass2Motif being associated with the suspect by the LDA model is 0.22. Table S13 shows annotations of fragments that are present in the Mass2Motif. A fragment present in the Mass2Motif couldn’t be explained by CFM-ID but could be linked to the sulfonamide substructure by the molecular formula from a structure analog in MassBank (Fig. S36).

3.6.4 Glycoside example

Fig. S37 show a glucose substructure related Mass2Motif being related with a probability of 0.77 to a glycoside suspect. The overlapping loss of 162.05 and the m/z difference of 18.01 are known losses corresponding to glucose and water, respectively[11].

3.7 Handling large datasets within reasonable timeframes

One of the main improvements in MS2LDA 2.0 is its runtime performance. This enhancement is partly due to the use of multiple cores during model execution, a feature enabled by the Tomotopy[12] package. We confirmed this by comparing the running performance between the original version and MS2LDA 2.0. In the original version only one single core can be used to process data, so first we compared MS2LDA original version and MS2LDA 2.0 using one single core. Fig. S38 shows MS2LDA 2.0 outperforming the original version for small size libraries (Fig. S38, green dot and squares) with only 1,267, whereas for larger libraries the difference between the two models is reduced, because both implementations become memory-bandwidth bound on a single core, making the optimized C++ routines in Tomotopy less advantageous over NumPy's compiled operations in the original implementation. For instance, we observed for the two largest spectral libraries, the Suspect-Library and BERKELEY-LAB-Library, how the runtime was nearly identical between MS2LDA 2.0 and its predecessor (Fig. S38, blue dots and squares).

However, when using MS2LDA 2.0 with multiple cores, its implementation outperforms the original version in each of the datasets (Fig. S39). Moreover, with four cores (that most laptops have available nowadays), datasets containing fewer than 20,000 spectra can be processed in under an hour, and processing time can be further reduced with more cores or additional server resources.

4. Supplementary Methods

4.1.1 Mass2Motif Object

To be able to store all information needed for Mass2Motifs, we created a Mass2Motif object. The Mass2Motif object is very similar to the matchms[13] spectrum object, but it has been changed in a few functions to be able to work with Mass2Motifs instead of mass spectra (see Table S14 for a detailed comparison). First, Mass2Motifs can consist of only losses and therefore the mandatory fragments needed for matchms spectrum objects had to be replaced. The second difference is that Mass2Motifs do not have a precursor mass and therefore associated losses with a Mass2Motif need to be stored since they cannot be calculated. All metadata as well as fragments and losses are retrieved in the same way as it is done in the matchms framework. This allows users to use most tools (for similarity search or other aims) that were developed with the matchms framework also with Mass2Motifs instead of mass spectra.

4.1.2 MS2LDA 2.0 parameter selection and definition

To guarantee the best modelling outcome and reproducible results when using MS2LDA 2.0 the selected parameter for preprocessing, annotation, and the LDA modelling play a key role. The parameters for all case studies and the MAG benchmarking are given below. Since we used the multi-core option for the LDA modelling (no seed can be set), the

results generated may (slightly) deviate from those presented in the manuscript when run on a different device.

Parameter selection for the MAG benchmarking

```
annotation_parameters = {
    "criterium": "best",
    "cosine_similarity": 0.90,
    "n_mols_retrieved": 5,
    "s2v_model_path": "...",
    "s2v_library_embeddings": “...",
    "s2v_library_db": "...",
}
```

Parameter selection for the MSn library

```
 preprocessing_parameters = {
    "min_mz": 0,
    "max_mz": 2000,
    "max_frags": 1000,
    "min_frags": 5,
    "min_intensity": 0.01,
    "max_intensity": 1
}


convergence_parameters = {
    "step_size": 50,
    "window_size": 10,
    "threshold": 0.005,
    "type": "perplexity_history"
}

annotation_parameters = {
    "criterium": "best",
    "cosine_similarity": 0.90
    "n_mols_retrieved": 10,
}

model_parameters = {
    "rm_top": 0,
    "min_cf": 0,
    "min_df": 3,
    "alpha": 0.6,
    "eta": 0.1,
    "seed": 42,
}
```

```
train_parameters = {
    "parallel": 3,
    "workers": 0,
}

dataset_parameters = {
    "acquisition_type": "DDA",
    "significant_digits": 2,
    "charge": 1,
    "name": "DDA-Suspectlist",
    "output_folder": f"CaseStudy_Corinna_Library_filtered_1000motifs_output_100625",
}

fingerprint_parameters = {
    "fp_type": "maccs",
    "threshold": 0.8,
}
```

Parameter selection for Case Study Pesticides

```
preprocessing_parameters = {
    "min_mz": 0,
    "max_mz": 1000,
    "max_frags": 1000,
    "min_frags": 4,
    "min_intensity": 0.01,
    "max_intensity": 1
}
convergence parameters = {
    "step_size": 50,
    "window_size": 10,
    "threshold": 0.001,
    "type": "perplexity_history"
}
annotation_parameters = {
    "criterium": "biggest",
    "cosine_similarity": 0.75,
    "n_mols_retrieved": 10,
    "s2v_model_path": "...",
    "s2v_library_embeddings": "...”,
    "s2v_library_db": "...",
}
n_motifs = 250
n_iterations = 5000
model_parameters = {
    "rm_top": 0,
    "min_cf": 0,
    "min_df": 3,
```

```
    "alpha": 0.4,
    "eta": 0.01,
    "seed": 42,
}
train_parameters = {
    "parallel": 3,
    "workers": 0,
}
dataset_parameters = {
    "acquisition_type": "DDA",
    "significant_digits": 3,
    "charge": 1,
    "name": "...",
    "output_folder": "...",
}
fingerprint_parameters = {
    "fp_type": "maccs",
    "threshold": 0.8,
}
motif_parameter = 10
```

Parameter selection for Case Study Natural Products

```
preprocessing_parameters = {
    "min_mz": 0,
    "max_mz": 2000,
    "max_frags": 1000,
    "min_frags": 5,
    "min_intensity": 0.01,
    "max_intensity": 1
}

convergence_parameters = {
    "step_size": 50,
    "window_size": 10,
    "threshold": 0.005,
    "type": "perplexity_history"
}

annotation_parameters = {
    "criterium": "biggest",
    "cosine_similarity": 0.7,
    "n_mols_retrieved": 10,
    "s2v_model_path": “...",
    "s2v_library_embeddings": “...",
    "s2v_library_db": “...",
```

```
}

n_motifs = 200
n_iterations = 10000

model_parameters = {
    "rm_top": 0,
    "min_cf": 0,
    "min_df": 3,
    "alpha": 0.6
    "eta": 0.01,
    "seed": 42,
}

train_parameters = {
    "parallel": 3,
    "workers": 0,
}

dataset_parameters = {
    "acquisition_type": "DDA",
    "charge": 1,
    "significant_digits": 2,
    "name": "CaseStudy_mushrooms",
    "output_folder": f"CaseStudy_Mushrooms_{n_motifs}",
}

fingerprint_parameters = {
    "fp_type": "maccs",
    "threshold": 0.8,
}

motif_parameter = 50
```

Parameter selection for the Case Study on Accelerating Biological Discoveries with MAG-empowered MS2LDA

```
preprocessing_parameters = {
    "min_mz": 0,
    "max_mz": 2000,
    "max_frags": 1000,
    "min_frags": 5,
    "min_intensity": 0.03,
    "max_intensity": 1
}

convergence_parameters = {
```

```
    "step_size": 50,
    "window_size": 10,
    "threshold": 0.005,
    "type": "perplexity_history"
}

annotation_parameters = {
    "criterium": "biggest",
    "cosine_similarity": 0.70,
    "n_mols_retrieved": 10,
    "s2v_model_path":
"/Users/rtlortega/Documents/PhD/models/Spec2Vec/model_positive_mode/positive_trai
n_data/150225_Spec2Vec_pos_CleanedLibraries.model",
    "s2v_library_embeddings":
"/Users/rtlortega/Documents/PhD/models/Spec2Vec/model_positive_mode/positive_trai
n_data/150225_CleanedLibraries_Spec2Vec_pos_embeddings.npy",
    "s2v_library_db":
"/Users/rtlortega/Documents/PhD/models/Spec2Vec/model_positive_mode/positive_trai
n_data/150225_CombLibraries_spectra.db",
}

model_parameters = {
    "rm_top": 5,
    "min_cf": 0,
    "min_df": 3,
    "alpha": 0.6, #A higher alpha makes the document preferences "smoother" over
topics
    "eta": 0.01, #and a higher eta makes the topic preferences "smoother" over words.
    "seed": 42,
}

train_parameters = {
    "parallel": 3,
    "workers": 1,
}

fingerprint_parameters = {
    "fp_type": "maccs",
    "threshold": 0.8,
}

motif_parameter = 30
n_motifs = 300
n_iterations = 1000

dataset_parameters = {
```

```
    "acquisition_type": "DDA",
    "significant_digits": 2,
    "charge": 1,
    "name": " CaseStudy_siderophores_{n_motifs}_230226",
    "output_folder": f"CaseStudy_siderophores_{n_motifs}_230226",
}
```

Parameter selection for Case Study Suspect list

```
preprocessing_parameters = {
    "min_mz": 0,
    "max_mz": 1000,
    "max_frags": 1000,
    "min_frags": 3,
    "min_intensity": 0.01,
    "max_intensity": 1
}
convergence_parameters = {
    "step_size": 50,
    "window_size": 10,
    "threshold": 0.001,
    "type": "perplexity_history"
}
annotation_parameters = {
    "criterium": "best",
    "cosine_similarity": 0.70,
    "n_mols_retrieved": 10,
    "s2v_model_path": "...",
    "s2v_library_embeddings": "...",
    "s2v_library_db": "...",
}
n_motifs = 1500
n_iterations = 5000
model_parameters = {
    "rm_top": 0,
    "min_cf": 0,
    "min_df": 3,
    "alpha": 0.6,
    "eta": 0.1,
    "seed": 42,
}
train_parameters = {
    "parallel": 3,
    "workers": 0,
}
dataset_parameters = {
    "acquisition_type": "DDA",
    "significant_digits": 2,
```

```
    "charge": 1,
    "name": "DDA-Suspectlist",
    "output_folder": f"CaseStudy_Suspectlist_{n_motifs}motifs_output",
}
fingerprint_parameters = {
    "fp_type": "maccs",
    "threshold": 0.8,
}
motif_parameter = 15
```

4.1.3 MS2LDA 2.0 convergence curves

To measure how well the LDA models converged, MS2LDA 2.0 outputs for every run a convergence curve showing the log-likelihood, perplexity, topic entropy, and document entropy score. In the Figs S40-S42, the convergence for all case studies are shown. For all case studies the perplexity score was used for early stopping as described in the Method section Convergence Criteria.

4.2.1 MotifDB

To query MotifDB, we developed MassQueryLanguage4Mass2Motifs (https://github.com/j-a-dietrich/MassQueryLanguage4Mass2Motifs), a fork of MassQL. MassQL provides a framework to query spectra but does not meet the requirements for querying Mass2Motifs. The major difference between spectra and Mass2Motifs is that Mass2Motifs do not possess a precursor ion and therefore no losses can be calculated. Since MassQL calculates losses in-time based on precursor ions (precursor ion - fragments), it cannot be used for Mass2Motifs. MassQL4Mass2Motifs stores losses permanently without the need for in-time calculations. Furthermore, Mass2Motifs are often associated with metadata such as annotations (manual or automated), instrument type, MassiveID, and more. We integrated the possibility to query not only for fragments and losses but also for the metadata by using the key word METAFILTER:columnname=value. Metafilters can be combined and should be listed at the end of the query (Fig. S43). Finally, we not only want to find Mass2Motifs of interest but also to use them further for screening purposes or molecular networking by using Mass2Motifs as nodes or edges between spectra. Therefore, we integrated a function to convert queried Mass2Motifs back to Mass2Motif objects. These Mass2Motif objects can then be easily integrated into common metabolomics workflows, for example to be used in MolNetEnhancer.

4.3.1 Visualization Interface

A new and revamped MS2LDAViz 2.0 interface has been developed as part of MS2LDA 2.0, providing users with an intuitive web-based platform for exploring and interpreting Mass2Motifs. Built using the Dash[14] framework, this visualization tool enables interactive browsing of Mass2Motifs, examination of automated annotations provided by Motif Annotation Guidance (MAG), and targeted searches of Mass2Motifs using MassQL-based queries. Compared to its predecessor, the new MS2LDAViz is easier to deploy and maintain, offers improved scalability, and allows users to run analyses locally on their computers or remotely on servers. This interface aims to streamline the annotation workflow, facilitate rapid exploration of complex MS/MS data, and promote community-driven annotation efforts through seamless integration with MotifDB.

The following sections describe different sections of the visualization app.

4.3.2 Run Analysis

Fig. S.44 shows the start screen of the visualization app. This screen allows users to initiate a new MS2LDA 2.0 analysis directly within the visualization application, provided that the application is installed locally with the necessary computational performance. Users can upload their MS/MS data file (supported formats include .mgf, .mzML and .msp) using the provided drag-and-drop interface. Basic MS2LDA 2.0 parameters such as the number of motifs, acquisition type (DDA or DIA), polarity, and iteration count can be adjusted directly. Additionally, Spec2Vec parameters are configurable from this screen; users can select and manage pre-trained Spec2Vec model and embedding files necessary for automatic motif annotation. Advanced settings (accessible by expanding the corresponding section) allow fine-grained control over preprocessing details, convergence criteria, and LDA model hyperparameters.

Once parameters are set, clicking “Run Analysis” initiates the modeling process. A folder containing all the results, including visualization results will be generated. By default, this folder is called “ms2lda_results” and the corresponding viz data file is ms2lda_viz.json.gz in that folder. This data file can be loaded for further analysis into the “Load Results” tab. This ensures that the running of MS2LDA 2.0 and the visualization and exploration of the results are separated.

4.3.3 Motif Ranking and Motif Details

The “Motif Ranking” (Fig. S45) screen provides a ranked list of inferred Mass2Motifs based on their significance across the analyzed dataset. Each motif is characterized by several key metrics:

- Degree: The number of spectra (documents) that are associated with the Mass2Motif.
- Average Doc-Topic Probability: The average probability of this motif appearing in associated spectra.
- Average Overlap Score: An averaged measure indicating how distinctly this motif explains the associated spectra.
- Annotation: Automatic annotations provided by Motif Annotation Guidance (MAG), summarizing potential chemical structures matching the Mass2Motif.

Interactive sliders at the top allow users to set probability and overlap thresholds, dynamically filtering motifs to quickly identify the most relevant and specific ones. Clicking a motif identifier (e.g., motif_148) leads users directly to a detailed view, where more information such as specific spectra associations, MAG results, and detailed annotations can be further explored.

Once a motif is selected from the ranking table, its details are shown in the “Motif Details” (Fig. S.46 and S47) screen. This screen provides comprehensive insights into a selected Mass2Motif, including automated structural annotations, spectral composition, and associated features. At the top, users see chemical structures suggested by Motif Annotation Guidance (MAG) based on Spec2Vec matching. Below this, a dual pseudo-spectrum plot compares optimized motif features with the raw fragments from associated spectra, facilitating interpretation and validation. The interface also lists individual fragment and loss features, highlighting their frequency and probability within the motif. Finally, users can interactively browse individual spectra linked to the motif, visualizing how each spectrum's fragments map onto the motif's characteristic features. This detailed breakdown allows for rapid, informed interpretation of discovered Mass2Motifs.

4.3.4 Spectra Search

The “Spectra Search” screen enables rapid retrieval and detailed exploration of spectra based on specified fragments, losses, or parent masses. Users can input fragment or loss patterns (e.g., frag@150, loss@40) or select a mass range for the precursor mass via the interactive slider (Fig. S48). Matched spectra are displayed in a searchable table, providing direct links to individual spectra. Selecting a specific spectrum reveals an interactive plot underneath the table, highlighting the fragment and loss peaks and their assignments to Mass2Motifs (like the spectrum plot in the Motif Details screen). Peaks color-coded by associated motifs clearly demonstrate how a single spectrum can be explained by multiple co-occurring Mass2Motifs, a distinctive feature of the MS2LDA framework.

4.3.5 View Network

A “View Network” screen shows an interactive network of optimized motifs (Supplementary Notes 3.1). Each Mass2Motif is displayed as a node (blue), and its fragments (green) or losses (yellow) appear as connected nodes (Fig. S49). Only edges above the selected intensity threshold will be shown. This can be customized using the slider. By default, the extra edges from each loss node to its corresponding fragment node are hidden for less clutter, but the user can re-enable them using the checkbox. By selecting any Mass2Motif, the associated MAG recommendations on the right side are shown. The user can select among multiple layouts, such as “Force-Directed”, for clustering the single nodes aside, “CoSE” for a similar layout as used in molecular networking, as “Circle” for checking all the connections between nodes and “Concentric”, for grouping the single nodes with no connections as an outer circle, while the nodes with connections are in a small circle inside.

4.3.6 Motif Search

The "Motif Search" screen facilitates automated annotation by enabling comparison of user-generated Mass2Motifs against selected reference motif sets from MotifDB. Unlike the Motif Annotation Guidance (MAG), which retrieves candidate annotations directly from spectral embeddings of general compound libraries, this approach specifically compares newly inferred motifs against previously annotated motif sets in MotifDB. Users select one or multiple reference MotifSets, run similarity calculations using their Spec2Vec embedding, and visualize matching results. Matches are displayed in a sortable table, showing user motifs, corresponding annotated motifs from reference databases, and similarity scores (Fig. S50). An interactive slider allows dynamic filtering based on similarity thresholds, efficiently narrowing down relevant annotations. This targeted comparison accelerates structural interpretation by linking motifs directly to community-curated annotated substructures in MotifDB MotifSets.

5. Supplementary Discussion

5.1 Parameter selection recommendations

The selection of hyperparameters is one of the most studied problems in topic modeling. The most commonly investigated hyperparameters are the number of Mass2Motifs and the alpha (α) and eta (η) values. The α value controls how many Mass2Motifs are present in each spectrum. A low α value causes each spectrum to be dominated by only a few Mass2Motifs, while a high α value allows each spectrum to contain many Mass2Motifs. The η value controls how many fragments are present in each Mass2Motif. A low η value produces sparse Mass2Motifs, containing only a few fragments, whereas a high η value produces dense Mass2Motifs, with many fragments shared across Mass2Motifs. We recommend starting with an α value of 0.6 for balancing spectra – Mass2Motif distributions and an η value of 0.01, which will increase the possibility that only certain fragments representing a single or few substructures. In general, α and η may need to be adjusted depending on the expected sparsity of the Mass2Motifs. Datasets where spectra contain many overlapping fragment patterns may benefit from slightly higher values, whereas datasets with more distinct fragmentation patterns often work better with lower values. Setting the number of Mass2Motifs to 100-300 generally works well for a wide variety of datasets and can be used as a starting point. In general, we recommend increasing the number of Mass2Motifs as the number of input spectra increases, since we would expect the number of detectable patterns to increase as well. In our experience, these standard settings perform well for typical datasets, with only small adjustments needed based on specific dataset characteristics.

Supplementary References

1. Huber, F. et al. Spec2Vec: Improved mass spectral similarity scoring through learning of structural relationships. PLoS Comput. Biol. **17**, (2021).
2. van der Hooft, J. J. J. et al. Unsupervised Discovery and Comparison of Structural Families Across Multiple Samples in Untargeted Metabolomics. Anal. Chem. **89**, 7569–7577 (2017).
3. Rogers, S. et al. Deciphering complex metabolite mixtures by unsupervised and supervised substructure discovery and semi-automated annotation from MS/MS spectra. Faraday Discuss. **218**, 284–302 (2019).
4. Kim, S. et al. PubChem 2023 update. Nucleic Acids Res. **51**, D1373–D1380 (2023).
5. Bento, A. P. et al. An open source chemical structure curation pipeline using RDKit. J. Cheminform. **12**, (2020).
6. Brungs, C. et al. MSnLib: efficient generation of open multi-stage fragmentation mass spectral libraries. Nat. Methods https://doi.org/10.1038/s41592-025-02813-0 (2025) doi:10.1038/s41592-025-02813-0.
7. Khatib, S. et al. Olive mill solid waste induces beneficial mushroom-specialized metabolite diversity revealed by computational metabolomics strategies. Metabolomics **21**, 58 (2025).
8. Damiani, T. et al. A universal language for finding mass spectrometry data patterns. Nat. Methods https://doi.org/10.1038/s41592-025-02660-z (2025) doi:10.1038/s41592-025-02660-z.
9. Monge-Loría, M., Zhong, W., Abrahamse, N. H., Hartter, S. & Garg, N. Discovery of Peptidic Siderophore Degradation by Screening Natural Product Profiles in Marine-Derived Bacterial Mono- and Cocultures. Biochemistry **64**, 634–654 (2025).
10. Wang, F. et al. CFM-ID 4.0: More Accurate ESI-MS/MS Spectral Prediction and Compound Identification. Anal. Chem. **93**, 11692–11700 (2021).
11. Mlynek, F., Himmelsbach, M., Buchberger, W. & Klampfl, C. W. A fast-screening approach for the tentative identification of drug-related metabolites from three non-steroidal anti-inflammatory drugs in hydroponically grown edible plants by HPLC-drift-tube-ion-mobility quadrupole time-of-flight mass spectrometry. Electrophoresis **42**, 482–489 (2021).
12. Lee, M., Fenstermacher, D., Schneider, J. & Almodóvar, J. C. bab2min/tomotopy: Preprint at https://doi.org/10.5281/zenodo.6869125 (2022).
13. de Jonge, N. F. et al. Reproducible MS/MS library cleaning pipeline in matchms. J. Cheminform. **16**, (2024).
14. Hossain, S. Visualization of Bioinformatics Data with Dash Bio. PROC. OF THE 18th PYTHON IN SCIENCE CONF https://dash.plot.ly/dash-bio. (2019).
