# Supplementary material for: Large-scale discovery and annotation of substructure patterns in mass spectrometry profiles
Source: Nat Commun. 2026 Jul 6;17:8350. doi: 10.1038/s41467-026-75038-0 (PMC13473578; doi:10.1038/s41467-026-75038-0)
Supplement: Supplementary file 2 [file 41467_2026_75038_MOESM2_ESM.pdf]

nature portfolio

Corresponding author(s): Rosina L. Torres Ortega
Jonas Dietrich
Justin J.J. van der Hooft

Last updated by author(s): 29 May 2026

Reporting Summary

Nature Portfolio wishes to improve the reproducibility of the work that we publish. This form provides structure for consistency and transparency in reporting. For further information on Nature Portfolio policies, see our Editorial Policies and the Editorial Policy Checklist.

Statistics

For all statistical analyses, confirm that the following items are present in the figure legend, table legend, main text, or Methods section.

n/a	Confirmed	
☐	☒	The exact sample size (*n*) for each experimental group/condition, given as a discrete number and unit of measurement
☒	☐	A statement on whether measurements were taken from distinct samples or whether the same sample was measured repeatedly
☒	☐	The statistical test(s) used AND whether they are one- or two-sided<br>*Only common tests should be described solely by name; describe more complex techniques in the Methods section.*
☒	☐	A description of all covariates tested
☒	☐	A description of any assumptions or corrections, such as tests of normality and adjustment for multiple comparisons
☐	☒	A full description of the statistical parameters including central tendency (e.g. means) or other basic estimates (e.g. regression coefficient) AND variation (e.g. standard deviation) or associated estimates of uncertainty (e.g. confidence intervals)
☒	☐	For null hypothesis testing, the test statistic (e.g. *F*, *t*, *r*) with confidence intervals, effect sizes, degrees of freedom and *P* value noted<br>*Give P values as exact values whenever suitable.*
☒	☐	For Bayesian analysis, information on the choice of priors and Markov chain Monte Carlo settings
☒	☐	For hierarchical and complex designs, identification of the appropriate level for tests and full reporting of outcomes
☒	☐	Estimates of effect sizes (e.g. Cohen's *d*, Pearson's *r*), indicating how they were calculated

Our web collection on statistics for biologists contains articles on many of the points above.

Software and code

Policy information about availability of computer code

Data collection	The public data used was available in .mgf format and could be directly downloaded from public repositories (see Data Availability). No special software tool was used.
Data analysis	Raw data was analyzed with mzmine 4.3.3. Figures were made with custom made Python scripts and Cytoscape version 3.11. The analysis of the data was done by MS2LDA 2.0 (https://github.com/vdhooftcompmet/MS2LDA) and all jupyter notebooks to replicate the results can be found in the GitHub repository as well. Below are the dependencies for MS2LDA 2.0 which were also used for other visualization such as with rdkit:<br>- python>=3.10,<3.13<br>- matplotlib>=3.9.1<br>- seaborn>=0.13.2<br>- pandas>=2.2.2<br>- scipy>=1.10.1<br>- networkx>=3.3<br>- gensim>=4.3.3<br>- psims>=1.3.3<br>- pubchempy>=1.0.4<br>- pydantic=2.10.3<br>- rdkit>=2023.9.6<br>- requests>=2.32.3<br>- matchms>=0.27.0

- plotly>=5.23.0
- pyarrow>=17.0.0
- pymzml>=2.5.10
- scikit-learn>=1.5.1
- openpyxl=3.1.5
- diskcache>=5.6.3
- dash-extensions==1.0.20
- ipython>=8.26.0
- jupyterlab>=4.2.4
- black=24.3.0
- flake8=7.0.0
- mypy=1.10.0
- pre-commit=3.7.0
- pytest>=8.3.2
- ruff>=0.11.0
- dash>=2.18.1
- dash_cytoscape>=1.0.2
- dash_bootstrap_components>=1.6.0
- tomotopy>=0.12.7
- spec2vec>=0.8.0
- PyQt6>=6.7.1
- faiss-cpu==1.10.0
- git+https://github.com/j-a-dietrich/MassQueryLanguage4Mass2Motifs.git

For manuscripts utilizing custom algorithms or software that are central to the research but not yet described in published literature, software must be made available to editors and reviewers. We strongly encourage code deposition in a community repository (e.g. GitHub). See the Nature Portfolio guidelines for submitting code & software for further information.

Data

Policy information about availability of data

All manuscripts must include a data availability statement. This statement should provide the following information, where applicable:

- Accession codes, unique identifiers, or web links for publicly available datasets
- A description of any restrictions on data availability
- For clinical datasets or third party data, please ensure that the statement adheres to our policy

For retraining the positive mode Spec2Vec model we used a dataset combining GNPS, MassBank and the MSnLibrary and the dataset can be downloaded from Zenodo [https://doi.org/10.5281/zenodo.12543129]. The used MSnLibrary in positive mode is openly available and was downloaded and subtracted to contain only MSn-derived library and positive ionization mode [https://doi.org/10.5281/zenodo.16882111].The pesticide dataset is available from GNPS-MassIVE (MSV000097937) [doi:10.25345/C5RJ49707]. The experimental fungal dataset used in this study is publicly available and was downloaded from MassIVE (MSV000097937) [DOI: 10.25345/C5W669D5F]. . The siderophore case study is available from GNPS-MassIVE (MSV000096127) [doi:10.25345/C5CF9JJ8G]. The data for the case study analyzing the suspect library can be found and downloaded from Zenodo [https://doi.org/10.5281/zenodo.8282733]. Results including all Mass2Motifs and their annotation (including Mass2Motifs for the benchmarking of MAG), which were generated for these datasets are available from Zenodo [https://doi.org/10.5281/zenodo.15857387], and as notebooks in the GitHub repository (https://github.com/vdhooftcompmet/MS2LDA/notebooks/Paper_results). Source data are provided with this paper.

Research involving human participants, their data, or biological material

Policy information about studies with human participants or human data. See also policy information about sex, gender (identity/presentation), and sexual orientation and race, ethnicity and racism.

Reporting on sex and gender	n/a
Reporting on race, ethnicity, or other socially relevant groupings	n/a
Population characteristics	n/a
Recruitment	n/a
Ethics oversight	n/a

Note that full information on the approval of the study protocol must also be provided in the manuscript.

Field-specific reporting

Please select the one below that is the best fit for your research. If you are not sure, read the appropriate sections before making your selection.

☒ Life sciences ☐ Behavioural & social sciences ☐ Ecological, evolutionary & environmental sciences

For a reference copy of the document with all sections, see nature.com/documents/nr-reporting-summary-flat.pdf

Life sciences study design

All studies must disclose on these points even when the disclosure is negative.

Sample size	The sample size was dependent on the study and included after filtering 2714 mass spectra for the mushroom dataset, 5611 spectra for the pesticide case study, and 77218 mass spectra for the suspect list case study. The benchmarking included three MotifsSets with 100 (Urine MotifSet), 74 (GNPS MotifSet), and 42 (Massbank MotifSet) Mass2Motifs. To retrain Spec2Vec we used a public library containing 502.685 mass spectra [https://zenodo.org/records/12605771].<br>For the experimental data we analyzed one spiked sample. As we were only interested in the recovery of the spiked compounds, we did not see a reason to have a larger sample size. Additionally, we measured the blank tomato sample and a chemical blank. All data used in the study can be found on MassIVE [doi:10.25345/C5RJ49707].
Data exclusions	During the modelling process, we excluded spectra based on several criteria, including the number of fragments, fragment intensities, m/z values, and the presence of fragments/losses across all input spectra. Spectra or fragments/losses that did not meet these criteria were excluded. The specific parameters used for each case study are detailed in Supplementary Information, Section 8: MS2LDA 2.0 Parameter Selection. This section also includes the parameter specifying how many of the highest-ranked associated fragments and losses were retained for downstream tasks (see motif parameters).
Replication	To support reproducibility, we have made all code used to generate the results publicly available at: https://github.com/vdhooftcompmet/MS2LDA/tree/main/notebooks/Paper_results. While exact replication may not be possible due to the stochastic nature of the modelling process and the fact that the library used does not guarantee identical outputs when run on multiple cores, as was done in our experiments, the code allows users to reproduce results that support the conclusions drawn in the study.
Randomization	Randomization was not applicable since the model takes all data from one sample as an input for find patterns in the entire dataset.
Blinding	Blinding was not applicable in this study as it did not involve experiments that could be influenced by subjective bias

Reporting for specific materials, systems and methods

We require information from authors about some types of materials, experimental systems and methods used in many studies. Here, indicate whether each material, system or method listed is relevant to your study. If you are not sure if a list item applies to your research, read the appropriate section before selecting a response.

Materials & experimental systems

n/a	Involved in the study	
☒	☐	Antibodies
☒	☐	Eukaryotic cell lines
☒	☐	Palaeontology and archaeology
☒	☐	Animals and other organisms
☒	☐	Clinical data
☒	☐	Dual use research of concern
☐	☒	Plants

Methods

n/a	Involved in the study	
☒	☐	ChIP-seq
☒	☐	Flow cytometry
☒	☐	MRI-based neuroimaging

Dual use research of concern

Policy information about dual use research of concern

Hazards

Could the accidental, deliberate or reckless misuse of agents or technologies generated in the work, or the application of information presented in the manuscript, pose a threat to:

No	Yes	
☒	☐	Public health
☒	☐	National security
☒	☐	Crops and/or livestock
☒	☐	Ecosystems
☒	☐	Any other significant area

Experiments of concern

Does the work involve any of these experiments of concern:

No	Yes	
☒	☐	Demonstrate how to render a vaccine ineffective
☒	☐	Confer resistance to therapeutically useful antibiotics or antiviral agents
☒	☐	Enhance the virulence of a pathogen or render a nonpathogen virulent
☒	☐	Increase transmissibility of a pathogen
☒	☐	Alter the host range of a pathogen
☒	☐	Enable evasion of diagnostic/detection modalities
☒	☐	Enable the weaponization of a biological agent or toxin
☒	☐	Any other potentially harmful combination of experiments and agents

Plants

Seed stocks	A blank tomato pulp sample was used as a matrix for spiking it with pesticides. No plants were harvest for this study.
Novel plant genotypes	n/a
Authentication	n/a
