# Supplementary material for: Large-scale discovery and annotation of substructure patterns in mass spectrometry profiles
Source: Nat Commun. 2026 Jul 6;17:8350. doi: 10.1038/s41467-026-75038-0 (PMC13473578; doi:10.1038/s41467-026-75038-0)
Supplement: Supplementary file 3 [file 41467_2026_75038_MOESM3_ESM.pdf]

nature portfolio

Peer Review File

Large-scale discovery and annotation of substructure patterns in mass spectrometry profiles

Corresponding Author: Dr Justin van der Hooft

This file contains all reviewer reports in order by version, followed by all author rebuttals in order by version.

Version 0:

Reviewer comments:

Reviewer #1

(Remarks to the Author)
The authors present an updated version of their previous work. According to the authors, the current MS2LDA 2.0 software is presented as framework designed to identify substructures in MS/MS datasets that is computationally more efficient than the previous version, easier to handle for users, and better to implement into current python workflows. Data are given that support the increase in computational speed between 3x to 14x, but this fact alone is insufficient to warrant publication. Judging user-friendliness is very hard to prove or disprove, and no user surveys or experiences are given. Last, the reviewer commends the authors to improve the adaptability of MS2LDA for other python-based workflows, but again, this feature is only a technical aspect.

In computer science, benchmarks are required to enable comparisons of software, using standardized tests. Key characteristics include relevance, representativeness, equity, repeatability, and verifiability. The authors fail to present adequate benchmark tests, for example, in comparison to similar or related tools. They also fail to use benchmark tests rigorously on large and well documented datasets such as the NIST23 library. Instead, they use user-based annotations (in MassBank and GNPS) which in themselves may be erroneous. Even after reading the text three times, the reviewer failed to understand how many motifs in the set of 211 spiked pesticides were positively classified, and more importantly, how many motifs were misclassified? For example, 9/17 dimethylated (mono/di-thio)-phosphate substructures had an association >0.7, but was this motif specifically targeted or found fully automated against the whole database? More importantly, when analyzing 77,218 MS/MS spectra from the 'suspect' list, how many would be (likely) misclassified as dimethylated (mono/di-thio)-phosphates? The reviewer finds the cases presented by the authors neither exhaustive nor specifically convincing. The reviewer believes the tool has merits to guide researchers towards contemplating that specific substructures may (or may not) be present in specific MSMS spectra. But neither parameters, nor benchmark results, are given to constrain the success rate or likelihood of correct annotations.

One could argue, for example, that the task of finding substructures in MSMS datasets might be to allocate "classes" and "subclasses" for unidentified metabolites, and use such classes and subclasses for statistical tests such as chemical enrichments. It is unclear if the true positive/true negative ROC curves for this software would enable such classifications. It is also unclear which characteristics of MSMS spectra would favor the software to retrieve true positive substructures, and which MSMS spectra would unlikely to get any true hit. It is known that MSMS spectra in metabolomics have far fewer fragment ions that peptide spectra (in proteomics). Hence, are there thresholds below which the software is rather unlikely to provide good results? Conversely, algorithms like CANOPUS already exist to classify spectra into compound classes. Such compound classes could easily be used to construct substructures using maximum common subgraph tools. How would the author's software compare to such a strategy? Similarly, it appears the author's software mainly refers to direct hits of fragment ion similarities (assisted by MSMS embeddings). But other algorithms, such as Steve Stein's Hybrid Search approach, already combine more elegantly the hits for open search (direct hits to fragments), neutral loss searches (specific spacing of fragments and precursor ions) and the combination of open- and neutral loss searches (aka hybrid search). The stated goal of these algorithms is to classify structures and substructures. How would the author's software compare? The Stein algorithm has been marketed as 'molecular networking' in GNPS web tools, again with the stated goal to bring together unknown MSMS spectra and their most related cousins, for manual interrogation due to structure (via MSMS)-relatedness.

Hence, the authors miss to thoroughly compare and contrast their software to existing solutions, let alone calculate the

likelihood that correct substructures would be found. Instead, the reviewer
gains the notion that, overall, the improvement of the current version over the previous implementation appears rather marginal and not a great leap forward. And while downloads and installations may now be easier, there is still no direct web application (unlike for other tools like GNPS).

(Remarks on code availability)
I did not test the code, sorry

Reviewer #2

(Remarks to the Author)
The manuscript presents the new version of MS2LDA, a method for finding recurring fragmentation patterns suggesting shared substructures ("Mass2Motifs") in MS/MS spectra. The new version contains several improvements, most notably an automated workflow (MAG) for annotating detected Mass2Motifs, which also most of our comments focus on. MAG uses Spec2Vec to search for potential annotations/annotation "guidances" in reference spectral libraries.

The other improvements include better multicore performance owed to utilizing the Tomotopy library for LDA computations, a better web app, and integration with the matchms framework and the mass spectrometry query language MassQL. We do not have major comments on those aspects. The runtime improvements are very valuable and nicely demonstrated with example datasets in the Supplement. Integrations with other methods are clearly useful; the only minor concern with the MassQL integration is that it requires a fork of MassQL, but this is due to limitations of MassQL, not the methods presented in this manuscript. We did not extensively test the web app but it seems well-made and intuitive to use.

We have several comments on the main method presented in the manuscript, the automated annotation workflow MAG.

- The major improvement of this workflow is that it leads to less necessary manual work although the authors make clear that it can only complement, not replace expert annotation. While the presented case studies convincingly demonstrate the benefits of MAG, manual curation seems to always be required. We therefore wonder how much time is really saved due to MAG, considering the manuscript quantifies the (purely/much more) manual annotation in the first MS2LDA version as multiple days for 30 Mass2Motifs. The general improvement in this aspect should be presented more clearly.

- The discussion mentions the sensitivity to (MS2LDA?) hyperparameters but different choices of hyperparameters are nowhere discussed or evaluated (excluding the number of iterations for convergence). How should users choose these parameters?

- The way the overlap score (SOS) is presented, suggesting large structures with lots of substructures through MAG seems to be advantageous for this metric as only the size of the smaller fingerprint is considered. With large structures containing many substructures which could potentially be (Mass2)motifs the task of annotation Mass2Motifs clearly becomes more laborious. Please discuss how such uninformative MAG structures can be (are?) prevented. Mainly through the masking/clustering approach?

- Supplementary Note 1.2 describes the "optimization" of Mass2Motifs. In the case studies it is mentioned that a particular issue can be circumvented by using this optimized version (the example of Mass2Motif 90). Is this done by default? Are there reasons to not use the optimizations?

- "Selected MotifDB-MotifSets generated with the initial MS2LDA version have been transferred into the new format": Is this a very work-intensive task or are there other reasons that "old" motifs are not available for MS2LDA 2.0? Perhaps we misunderstood this point in the manuscript.

Overall the manuscript presents very useful improvements to motif discovery with MS2LDA and extensively showcases how these improvements can help in practice with various case studies. While the methodological improvements mostly consist in utilizing existing tools (e.g., Spec2Vec for MAG, Tomotopy for better performance), we do not question the relevance and benefits of the approach; some aspects, detailed above, should be better presented, though.

Some minor comments:

- Parameters for the clustering after Spec2Vec search are not described (distance threshold, number of clusters).
- MAG currently only works for positive ionization mode, this should be earlier stated in the manuscript.
- "By default, the top five database hits are retrieved as candidate matches": Should be ten, right?
- "Mass2Motif 90 associated to the hericenone family, provided a poor annotation (Fig. S15)." Should be S14.

(Remarks on code availability)
We just took a short glimpse at the git repository, it is looking fine.

Version 1:

Reviewer comments:

Reviewer #1

(Remarks to the Author)
The authors expanded the use cases and benchmarking efforts. The authors argue that existing benchmarking datasets do not easily apply for unsupervised substructure discoveries, and that making benchmark datasets was out of scope of their work.
The reviewer disagrees. For computer science, solid benchmarking (and not just evaluation, or just showing test cases) is critical. Benchmarking consists of the following items:

(a) relevance (meaningful, important to users). While the authors have shown relevance for true positive spike-in of pesticides, it is still unclear how false-positives could be measured.
(b) representativeness (are the performance metrics broadly accepted and understood by the community?). Here, the authors write "determining whether a MAG annotation captures a distinct substructure remains challenging". From that sentence, it appears that understanding when and if MAG would work is not clear to the authors, let alone benchmarked.
(c) equity (comparing different systems). The authors still argue that there are no other systems to compare to. The authors argue that compound classification was different than substructure analyses. Yet, the examples they showed (from alpha-amino acids to sulfonureas or indoles) are very much like definitions of classifications. Hence, the authors missed this important criterion, comparison to other systems.
(d) repeatability. (verification and comparison of results across different runs). It is unclear if authors have even tried that test for their system, let alone on different implementations (hardware or software environments)
(e) Verifiability. (users should be able to verify the accuracy of benchmarking results). Here the authors argue that "no ground truth exists" and that "...the number of fragments, the size of
the shared substructure, and the number of recommended structures, provide helpful
tendencies but do not yield strict thresholds." Hence, users cannot verify the usefulness of the tool, because MAG is just a guidance, a tool for ...estimating or guessing substructures, without confidence thresholds. That is not instilling confidence in the community, the reviewer would like to argue.
(f) Transparency (details of benchmark designs, implementations, configurations used). The authors argue that for this reason, NIST23 could not be used. Instead, other datasets were used that were also not easily accessible (e.g. hosted on a benchmarking cluster site). The reviewer understands that community users could go at lengths to retrieve the data themselves, but without classic benchmarking results , (true/false positives, true/false negatives and corresponding ROC results). The authors argue that they did not try to find true/false positives etc because it is a discovery engine. Yet, the examples were based on predefined classes (i.e. pesticides, indoles etc). The reviewer fails to acknowledge that the authors showed any clear case of discoveries or benchmarking for the ability for discoveries.
(g) Scalability (adaptable for systems with different resource levels). While the authors argue that their system is now more scalable than their version before, 14-times faster, it is unclear how the tool would scale on different computer systems (i.e. how users would use it).
(h) end-to-end performance of the overall system, rather than individual components: The authors fail to address this point, as they argue that their discovery system cannot be benchmarked in this way, and scales would depend on the different types of substructures to be found.

Overall, the reviewer is not convinced that this tool would be highly useful for the community.

(Remarks on code availability)
na

Reviewer #2

(Remarks to the Author)
The manuscript was substantially refined and almost all of our comments adequately addressed. We only have a few minor suggestions that do not require another round of answers.

In the answers and also the revised manuscript the benefits of the new version, especially MAG, are now clearer presented. We think that the last point from the first answer, reducing the time for manual inspection of motifs from hours/days (?) to minutes, could also be included explicitely into the manuscript, perhaps at the end of the Introduction or beginning of the Results. This is indeed very impressive.
We see now that optimization of motifs is always performed. This does however sound different in the description of one case study; this might need to be adapted to avoid confusion: "This issue can be circumvented by using the optimized fragments of the Mass2Motif (Supplementary Notes 1.2)."
The new supplementary table on parameters is much appreciated, we could however not find the parameters for agglomerative clustering. We specifically refer to the following sentence in the manuscript: "This behavior is identified by applying agglomerative clustering (scikit-learn version 1.5.1)"

(Remarks on code availability)
Code is looking ok, well-documented, tests etc

Reviewer #3

(Remarks to the Author)
I co-reviewed this manuscript with one of the reviewers who provided the listed reports. This is part of the Nature Communications initiative to facilitate training in peer review and to provide appropriate recognition for Early Career

Researchers who co-review manuscripts.

(Remarks on code availability)
I co-reviewed this manuscript with one of the reviewers who provided the listed reports. This is part of the Nature Communications initiative to facilitate training in peer review and to provide appropriate recognition for Early Career Researchers who co-review manuscripts.

Version 2:

Reviewer comments:

Reviewer #2

(Remarks to the Author)
I assessed the authors' responses to Reviewer 1. The authors have done a satisfactory job to answer the reviewer's concerns, and for (e) they have added another study and a lot of material, demonstrating what MAG can do. For (ab) the authors slightly changed the text, and I think that this is good enough. For (fgh) I agree with the authors and find their responses sufficient, and further efforts to address these concerns are out of scope of the current work. MAG is not a tool that a user applies every day, in particular not if collecting the data for that is already taking you days or weeks. For data availability, GNPS is THE repository if you want to make it simple to get the data.

The only point where it makes more sense to answer in the manuscript than to the reviewer only, is (c). The answer to the reviewer says, "we are doing this, and here are the differences to CANOPUS for predicting compound classes" -- but that should be explained *in the manuscript instead*. In particular, since a very similar issue is coming up again in (f) for predefined classes.

Beyond this minor change, I think the manuscript is acceptable.

(Remarks on code availability)

Open Access This Peer Review File is licensed under a Creative Commons Attribution 4.0 International License, which permits use, sharing, adaptation, distribution and reproduction in any medium or format, as long as you give appropriate credit to the original author(s) and the source, provide a link to the Creative Commons license, and indicate if changes were made.
In cases where reviewers are anonymous, credit should be given to 'Anonymous Referee' and the source.
The images or other third party material in this Peer Review File are included in the article’s Creative Commons license, unless indicated otherwise in a credit line to the material. If material is not included in the article’s Creative Commons license and your intended use is not permitted by statutory regulation or exceeds the permitted use, you will need to obtain permission directly from the copyright holder.
To view a copy of this license, visit https://creativecommons.org/licenses/by/4.0/

Response to Reviewers

Manuscript ID: NCOMMS-25-56219-T

Title: "Large-scale discovery and annotation of hidden substructure patterns in mass spectrometry profiles"

Authors: Laura Rosina Torres Ortega, Jonas Dietrich, Joe Wandy, Hans Mol & Justin J. J. van der Hooft

We thank the editor and reviewers for their thorough evaluation of our manuscript and for the overall constructive comments, which have helped us improve the clarity, rigor and impact of our work. The main issue that we identified as mainly raised by reviewer #1 was the need for more explicit benchmarking and clearer ways of evaluating the MS2LDA 2.0 framework.

We acknowledge these points and have addressed them by incorporating new analyses and clarifications. We expanded our evaluation of the Motif Annotation Guidance (MAG) component, provided additional demonstrations on annotation behaviour and more explicitly contextualised the benchmarking strategy based on unique challenges of substructure discovery in metabolomics.

These new results highlight that:

- **Re Main Point 1: We have clarified and strengthened the benchmarking rationale for MS2LDA 2.0,** explicitly distinguishing the unchanged LDA motif-mining component from the newly introduced Motif Annotation Guidance**.** We now explain why existing standardised benchmarks do not easily apply for unsupervised substructure discovery, and why the verified MotifDB annotations are appropriate for evaluating MAG. Moreover, we have incorporated a new section on benchmarking of a large library of standards MS^n, in order to evaluate the MAG based on optimised fragments present and length of the maximum common substructure. These clarifications directly address the reviewer's concerns about benchmarking.
- **Re Main Point 2: We have expanded and clarified the description of the pesticide case study and behaviour of MAG**, including how motifs emerge in a fully unsupervised manner, why misclassifications cannot be defined in a classical sense, and what characteristics of spectra influence motif recovery. We also more clearly describe the challenges in defining universal confidence scores for motif annotations and explain how user's interpretation is needed in practice.

We are thankful for the reviewers' time and their comments and think that these additional analyses make the manuscript of a higher quality. We believe the additional analysis, clarifications, and revisions directly address the concerns raised and strengthen both technical and scientific contribution of MS2LDA 2.0. We think that the revisions, particularly the expanded methodological explanations and further analysis of the Mass2Motif properties, together with the additional clarifications in the case studies, an improved discussion of benchmarking, a new section exploring the MAG evaluation in library spectra and the current challenges of unsupervised substructure evaluation, significantly strengthen the manuscript. We think these changes more clearly demonstrate the relevance and value of MS2LDA 2.0 for large-scale substructure discovery in metabolomics studies.

Additional reviewer specific comments are addressed here below. Furthermore, a document that lists all changes made is available as a Manuscript Related File.

Reviewer #1

Comment 1:

"Data are given that support the increase in computational speed between 3x to 14x, but this fact alone is insufficient to warrant publication. Judging user-friendliness is very hard to prove or disprove, and no user surveys or experiences are given. Last, the reviewer commends the authors to improve the adaptability of MS2LDA for other python-based workflows, but again, this feature is only a technical aspect"

Response 1:

We appreciate that the reviewer acknowledged the improvements in computational speed and adaptability of MS2LDA 2.0. We agree that user-friendliness is difficult to quantify, and improved technical aspects alone may not guarantee high impact. However, the main advance of MS2LDA 2.0 is the introduction of guided annotation for motifs, which directly targets one of the key bottlenecks in computational metabolomics: the rate of structural annotation. By providing an automated guidance for motif interpretation, MS2LDA 2.0 significantly accelerates this process compared to the primarily manual workflow of the original MS2LDA framework. We believe this contribution is central to the scientific value of the updated software. In addition, the improvement in computational speed is crucial in modern metabolomics experiments, where the large amount of data can otherwise restrict the applicability of software tools to large datasets. Together, these advances move MS2LDA 2.0 beyond a purely technical update and enable analyses that were previously impractical.

Comment 2:
"In computer science, benchmarks are required to enable comparisons of software, using standardized tests. Key characteristics include relevance, representativeness, equity, repeatability, and verifiability. The authors fail to present adequate benchmark tests, for example, in comparison to similar or related tools. They also fail to use benchmark tests rigorously on large and well documented datasets such as the NIST23 library. Instead, they use user-based annotations (in MassBank and GNPS) which in themselves may be erroneous"

Response 2:
We agree that benchmarking is important for evaluating machine learning tools. In the original MS2LDA paper, the LDA algorithm for mining fragmentation patterns was already benchmarked using injected standards (van der Hooft *et al.*, 2016, PNAS). As this core algorithm remains unchanged in MS2LDA 2.0, the validation of our work focused on the novel feature, the Motif Annotation Guidance (MAG) component, which we benchmarked using manually verified and validated motifs from the MotifDB database. While any user-generated resource may contain occasional errors, these MotifSets were curated and cross-validated in the original MS2LDA and MotifDB publications and represent, to our knowledge, the best available motif-level reference sets for this task. The validation of the MotifDB MotifSets is described in the cited references, and we now emphasise this more clearly in the revised manuscript.

Designing and validating a new standardised benchmark suite (for example, analogous to MassSpecGym (Bushuiev, Bushuiev, *et al.*, 2025, Advances in Neural Information Processing Systems) for substructure discovery is beyond the the scope of this contribution. At present, we are not aware of any open benchmark for substructure discovery or substructure annotation that do not rely on extensive manual annotations. We agree that such benchmarks would be valuable for the community and would welcome future efforts in this direction.

To address the reviewer's point about large, well-documented datasets, we have additionally included a new benchmark in the revised manuscript in which MS2LDA 2.0 is applied to the recently published open MS^{n}Lib spectral library that operates under an open licence (Brungs *et al.*, 2025, Nature Methods). In this analysis, we assess how well MAG annotations recover substructures present in reference standards and relate annotation quality to motif properties (see Results, "Modeling and Annotation Performance on Library Spectra and Their Interpretation"). This complements the MotifDB-based evaluation and provides a large-scale, library-based assessment using fully documented spectra and structures.

We deem comparisons to other tools as challenging because, to our knowledge, there are no directly comparable software solutions for unsupervised motif discovery and guided annotation (see responses 4 and 5 for a discussion of related methods).

Regarding dataset choice, we would like to add that, while NIST23 is indeed of high-quality, it is proprietary and not openly accessible, and models trained on it cannot be publicly shared according to NIST regulations. This conflicts with our goal of reproducible and open science, which is why we chose publicly available libraries and the MS^n dataset instead.

In summary, MS2LDA 2.0 was benchmarked on verified motif annotations and on a large library of reference standards, which we consider appropriate and realistic test beds for its intended purpose.

Comment 3:
"Even after reading the text three times, the reviewer failed to understand how many motifs in the set of 211 spiked pesticides were positively classified, and more importantly, how many motifs were misclassified? For example, 9/17 dimethylated (mono/di-thio)-phosphate substructures had an association >0.7, but was this motif specifically targeted or found fully automated against the whole database? More importantly, when analyzing 77,218 MS/MS spectra from the 'suspect' list, how many would be (likely) misclassified as dimethylated (mono/di-thio)-phosphates? The reviewer finds the cases presented by the authors neither exhaustive nor specifically convincing. The reviewer believes the tool has merits to guide researchers towards contemplating that specific substructures may (or may not) be present in specific MSMS spectra. But neither parameters, nor benchmark results, are given to constrain the success rate or likelihood of correct annotations"

Response 3:
We appreciate the reviewer's careful reading and the opportunity to clarify the pesticide case study and how MS2LDA 2.0 works. In this study, a tomato blank was spiked with 211 pesticides, generating nearly 10,000 MS/MS features. Not all spiked compounds were triggered or were measurable (some only in negative mode); thus, roughly 200 MS/MS features correspond to pesticides.

MS2LDA 2.0 is fully unsupervised, which means no motifs were targeted. The main aim of this analysis was to assess if MS2LDA 2.0 can automatically recover motifs that correspond to known substructures in complex

matrices. Hence, the algorithm was run on all features, and motifs were automatically extracted based on recurring fragmentation patterns. The 9/17 dimethylated (mono/di-thio)-phosphate motifs cited by the reviewer emerged naturally because multiple spiked compounds contained this substructure. We then examined all molecules associated with this motif and found that there were no molecules with a high association that did not match substructure for the shown examples.

Regarding “misclassification”, MS2LDA is an unsupervised substructure discovery tool and as such, it does not assign hard labels as classification tools would do. It computes correlations between motifs and spectra, with an associated probability score indicating the strength of the relationship. We acknowledge that the interpretation of the extracted motifs in terms of correctly annotated or not can be difficult to judge and should be more clearly emphasized and now have clarified this more explicitly in the revised manuscript.

In the past months, we have actively worked and thought about how to provide better guidance for users in assessing whether an annotation is describing a substructure accurately or not. However, our progress has shown that this remains a highly challenging task. We therefore added a new section to the manuscript (see **Results - Modelling and Annotation Performance on Library Spectra and Their Interpretation** section), where we ran MS2LDA 2.0 on the recently published MS^n library and investigated whether the MAG annotation corresponds to the associated molecules, also adding a new Figure 2. In this analysis, we investigated whether three properties of the optimized Mass2Motifs correlate with annotation quality. The results show clear tendencies that Mass2Motifs with a higher number of MAG-recommended structures (more than five) are more likely to be well annotated and can be distinguished based on the number of shared fragments, although manual confirmation remains necessary overall.

We also appreciate the reviewer’s question about how many high-quality Mass2Motif annotations are typically generated. For the pesticide dataset this cannot be answered quantitatively as there is for the substructural information no ground truth. However, based on the new section added on the new MSn-library analysis, between 7% and 15% of the generated motifs show a high correlation with the underlying substructures, providing an estimate of the fraction of high-confidence annotations that can be expected in large, heterogeneous datasets. Previous analyses by MS2LDA v1, also those that served as input for MotifDB MotifSets, showed similar percentages of such high-quality Mass2Motifs; however, it is important to add that other Mass2Motifs can be very useful as well as they could describe particular compound groups or they could fom the basis to refine the pattern to link to a specific scaffold, i.e., by using MassQL (Damiani *et al.*, 2025, Nature Methods).

As described in the manuscript (and reiterated in Response 2), MAG is benchmarked conceptually and methodologically. The case study on pesticides already included in the manuscript demonstrates that MS2LDA 2.0 can automatically recover motifs that correspond to known substructures in complex matrices, without prior compound-level targeting, highlighting its ability to guide researchers toward meaningful and interpretable substructural patterns.

As a consequence of the insertion of the large-scale appiication of MS2LDA on the MS^nLib dataset, we decided to shorten the suspect library section in the main manuscript, and move the Figure to the Supplementary Information, also to make space for the newly introduced Figure 2.

Comment 4:
“One could argue, for example, that the task of finding substructures in MSMS datasets might be to allocate "classes" and "subclasses" for unidentified metabolites, and use such classes and subclasses for statistical tests such as chemical enrichments. “It is unclear if the true positive/true negative ROC curves for this software would enable such classifications. It is also unclear which characteristics of MSMS spectra would favor the software to retrieve true positive substructures, and which MSMS spectra would unlikely to get any true hit. It is known that MSMS spectra in metabolomics have far fewer fragment ions that peptide spectra (in proteomics). Hence, are there thresholds below which the software is rather unlikely to provide good results?”

Response 4:
We would like to clarify that the goal of MS2LDA is not to classify metabolites into predefined chemical classes nor to perform supervised prediction tasks. Instead, the method discovers recurring substructural patterns in an unsupervised manner to aid interpretation and hypothesis generation. The resulting Mass2Motifs can be used for downstream analysis such as chemical enrichment analyses if users choose to map Mass2Motifs to known substructures or chemical families. Because the method itself does not operate on pre-defined binary labels, ROC-style true-positive evaluations are not directly applicable, as no ground-truth substructure annotations exist for most metabolites. For this reason, we instead assess performance at the level of motif–substructure agreement (e.g. through the SOS score in the MSn-library benchmark), rather than through classical ROC curves.

With respect to the question of when the method retrieves “true” substructures, our experience and benchmarking indicate that several spectral characteristics influence the quality of detected motifs. MS2LDA perform best when there are multiple molecules sharing a common substructure and can produce consistent fragment ions. Thus, spectra that contain very few fragment ions, spectra with high noise (not pre-processed), or unique metabolites that appear in few spectra will provide less statistical support and therefore less likely to produce well-defined motifs. These properties are present in untargeted metabolomics data, and they vary by experiment, instrument, dataset and acquisition settings. For this reason, it is difficult to define general thresholds below which the software “fails” or above which it “succeeds”. In practice, we typically apply an intensity threshold after normalisation of >0.01, as stated in the case studies; and, as shown in the MSn-library analysis, Mass2Motifs with more fragments and multiple consistent MAG-recommended structures tend to yield higher-quality annotations. However, in some instances, one specific mass fragment or neutral loss may well be very specific. For example, the chlorobenzene substructure can be recognized by one unique fragment. We also note here that MS2LDA 2.0 still inherits the challenges that mass spectrometry-based analyses all have: there normally is ambiguity when it comes to stereoisomeric substructures, and there can be ambiguity about positional isomers as well. However, in these cases, the structural information provided by MS2LDA is often still very valuable.

Overall, MS2LDA aims to extract statistically supported substructures that enhance structure insight in discovery-driven workflows, rather than produce hard class assignments or operate within a classical supervised classification framework. We did our best to clarify both the scope of the approach and some properties of the spectra in which the method performs most effectively.

Comment 5:
“Conversely, algorithms like CANOPUS already exist to classify spectra into compound classes. Such compound classes could easily be used to construct substructures using maximum common subgraph tools. How would the author's software compare to such a strategy?”

Response 5:
We appreciate the reviewer’s suggestion that CANOPUS-based compound classes could be combined with maximum common subgraph (MCS) to derive substructures. However, this strategy addresses a fundamentally different problem than the one MS2LDA 2.0 is designed to solve.

CANOPUS is a deep learning model that predicts compound classes from fragmentation spectra, within a predefined ontology. This is highly effective for class assignment across a total of 2,497 chemical classes; some classes contain structurally diverse molecules for which MCS analysis will return only trivial scaffolds (e.g., “benzene ring”).

In contrast, MS2LDA 2.0 discovers fragmentation-driven substructures in an unsupervised manner, without depending on predefined class labels or external ontologies. Mass2Motifs are learned directly from reoccurring MS/MS patterns across spectra, allowing the detection of functional groups or moieties that may occur across multiple chemical classes (e.g. glycosylation, indoles, phosphates), that will not be recovered by a class-based MCS strategy. This ability to capture shared substructures between different classes is particularly important in metabolomics, where many biologically meaningful modifications are not class unique, and where many substructures are still not described (by current chemical ontologies).

We acknowledge that fragmentation similarity does not necessarily correlate with structural similarity: structurally similar compounds can fragment differently, while different structures can produce similar spectra. Therefore, relying solely on class membership and MCS may overlook fragmentation-relevant substructures present in the patterns driving spectra similarity.

Finally, while MAG relies on matching Mass2Motifs to known structures for annotation and therefore cannot fully annotate completely novel chemistry, MS2LDA 2.0 remains able to extract the recurrent fragmentation motifs from such compounds, something class-based MCS methods cannot produce. Together, these points highlight that CANOPUS-based MCS and MS2LDA 2.0 address complementary but distinct tasks, with MS2LDA 2.0 offering a direct route to fragmentation-evidenced substructures that are not limited by pre-defined chemical ontologies.

Comment 6:
“Similarly, it appears the author's software mainly refers to direct hits of fragment ion similarities (assisted by MSMS embeddings). But other algorithms, such as Steve Stein's Hybrid Search approach, already combine more elegantly the hits for open search (direct hits to fragments), neutral loss searches (specific spacing of fragments and precursor ions) and the combination of open- and neutral loss searches (aka hybrid search). The stated goal of these algorithms is to classify structures and substructures. How would the author's software compare? The Stein algorithm has been marketed as 'molecular networking' in GNPS web tools, again with the stated goal to bring together unknown MSMS spectra and their most related cousins, for manual interrogation

due to structure (via MSMS)-relatedness"

Response 6:
Hybrid Search and GNPS molecular networking are powerful tools for measuring pairwise spectral similarity based on neutral losses and direct fragment matches. These approaches perform well at linking a query spectrum to its closest analogue present in a mass spectral library or dataset and can successfully annotate unknown compounds when structurally related reference spectra are available.

MS2LDA, in contrast, addresses a different problem. Rather than comparing spectra pairwise, MS2LDA performs dataset-level pattern mining to extract recurring substructural motifs across a dataset. These motifs represent latent fragmentation patterns supported by many spectra, even when those spectra are not similar enough to obtain a high score by cosine or hybrid search. In this way, MS2LDA reveals substructures shared across large chemical families, or shared across multiple classes that pairwise similarity methods would never connect. This dataset-level abstraction is also why MS2LDA has been integrated into GNPS: it complements molecular networking by connecting spectra that will remain unlinked through cosine or hybrid approaches. This has been shown in several publications, including the MolNetEnhancer study that described how GNPS Molecular Networking and MS2LDA results can be combined (Ernst *et al.*, 2019, Metabolites).

We note that hybrid search does not identify substructures, it identifies related spectra. Even when incorporating neutral losses, it returns the nearest neighbours of a given spectrum rather than underlying chemical moieties responsible for recurring fragmentation patterns. MS2LDA, however discovers motifs that can be annotated or mapped to chemical substructures, independently of whether a representative reference spectrum exists. MS2LDA is particularly valuable for datasets containing unknown or poorly represented chemistry, where no suitable direct analogue is present in mass spectral libraries, but molecules with similar or the same scaffolds are (for MAG that is).

If the reviewer refers specifically to the use of Spec2Vec within MAG, we agree that hybrid search and embedding-based similarity represent two different strategies for retrieving structurally related spectra. We chose Spec2Vec because it captures both fragments and neutral losses within an embedding framework, which allows precomputed vector representations and efficient large-scale nearest-neighbour search via FAISS. In MAG, this serves as a feature of the annotation workflow, not as a replacement for hybrid search.

Overall, hybrid search and MS2LDA 2.0 are complementary, hybrid search finds the nearest spectral neighbours of a query spectrum, while MS2LDA uncovers recurrent substructural patterns that may not be detectable through pairwise similarity.

Comment 7:
"The reviewer gains the notion that, overall, the improvement of the current version over the previous implementation appears rather marginal and not a great leap forward. And while downloads and installations may now be easier, there is still no direct web application (unlike for other tools like GNPS)"

Response 7:
The direct web application for MS2LDA has been made available since the initial submission and can be accessed at https://ms2lda.org/, where the focus is on intuitive result visualization and interactive exploration of Mass2Motifs. In addition, the installation and execution of MS2LDA have been substantially improved and now fully documented in the updated repository: https://github.com/vdhooftcompmet/MS2LDA/tree/main. Over the last months, we have also created a user and developer manual to ease use and future contributions: https://vdhooftcompmet.github.io/MS2LDA/. Beyond these usability aspects, MS2LDA 2.0 also introduces several conceptual and practical advances over the original implementation: the Motif Annotation Guidance (MAG) for semi-automated motif annotation, a more scalable LDA implementation enabling analysis of much larger datasets, and integration with widely used frameworks such as matchms, MassQL and an updated MotifDB. Together, these developments go beyond a marginal technical update and provide both new functionality and broader applicability compared to the original MS2LDA.

Reviewer #1 (Remarks on code availability):

I did not test the code, sorry

Reviewer #2:

Comment 1:
"The major improvement of this workflow is that it leads to less necessary manual work although the authors make clear that it can only complement, not replace expert annotation. While the presented case studies convincingly demonstrate the benefits of MAG, manual curation seems to always be required. We therefore wonder how much time is really saved due to MAG, considering the manuscript quantifies the (purely/much more) manual annotation in the first MS2LDA version as multiple days for 30 Mass2Motifs. The general improvement in this aspect should be presented more clearly"

Response 1:
We acknowledge that MAG does not fully replace expert annotation, but it substantially reduces the amount of expert inspection required. In practice, MAG helps to the point that non-experts in the field can often evaluate a Mass2Motif within a few minutes. MAG presents a list of recommended structures and when these share a common substructure, the user can immediately recognize that the Mass2Motif annotation is likely meaningful. Subsequently, users can verify consistency by inspecting fragments shared across the recommended structures. A brief search in public spectra libraries (e.g., Massbank) can be sufficient to increase confidence in the match.

Without MAG, users must first hypothesize the expected structural motifs and then manually inspect fragment by fragment whether the data supports this hypothesis, which requires substantially more time and expert knowledge.

In cases where MAG's recommendations do not share a substructural core, for instance, when the underlying substructure is rare or not present in libraries, manual curation may be required. Even then, MAG provides a valuable starting point by suggesting candidate structures that can guide expert reasoning. As is inherent to the LDA algorithm, not all Mass2Motifs will represent a meaningful substructure, and MAG allows also to easily discharge such motifs, preventing unnecessary manual effort. This can be based on the amount and type of common fragments or on missing structural overlaps among the recommended compounds. Overall, MAG clarifies and accelerates annotation of Mass2Motifs by enabling rapid initial evaluation and reducing the need for a priori knowledge of the substructure and helping users quickly identifying both promising and uninformative Mass2Motifs. We now have emphasized in the revised manuscript that compared to the primarily manual workflow of the first MS2LDA version (which required multiple days for 30 Mass2Motifs), MAG reduces the per-motif inspection time to minutes and enables substantially larger sets of motifs to be annotated within practical timeframes.

Comment 2:
"The discussion mentions the sensitivity to (MS2LDA?) hyperparameters but different choices of hyperparameters are nowhere discussed or evaluated (excluding the number of iterations for convergence). How should users choose these parameters?"

Response 2:
We appreciate the reviewer for this comment on how to select hyperparameters, in response we have added a section in the Supplementary Information (MS2LDA 2.0 parameter selection and definition) that provides practical guidance on selecting MS2LDA hyperparameters. While some hyperparameters can have a significant impact on the results, appropriate choices depend on assumptions about the chemical diversity and structural complexity of the investigated sample. In practice, there is no single universally optimal setting. We therefore recommend exploring a small range of hyperparameters, for example, running models with different numbers of Mass2Motifs and different numbers of iterations. From our experience, users can already achieve robust results by testing a few settings rather than performing exhaustive optimization.

Comment 3:
"The way the overlap score (SOS) is presented, suggesting large structures with lots of substructures through MAG seems to be advantageous for this metric as only the size of the smaller fingerprint is considered. With large structures containing many substructures which could potentially be (Mass2)motifs the task of annotation Mass2Motifs clearly becomes more laborious. Please discuss how such uninformative MAG structures can be (are?) prevented. Mainly through the masking/clustering approach"

Response 3:
We agree that large structures, particularly when compared to small substructures, can lead to an overestimation of the overlap score (SOS). In our own experiments, we observed that MACCS fingerprints tend to overestimate structural overlap, whereas Daylight fingerprints can underestimate it, as constitutional isomers that fragment identically are not considered equivalent. To account for this bias, we compared our MAG annotations to randomly selected compounds and observed an improvement of approximately 0.3 (depending on the dataset), confirming that meaningful signals remain despite these limitations.

Regarding the reviewer's concern about avoiding uninformative large structures: the masking and clustering steps used within the MAG workflow play an important role here. These steps help ensure that the Mass2Motifs themselves are coherent and only contain fragments and losses present across all recommended structures. On the other hand, for MAG benchmarking, we use pre-defined Mass2Motifs, meaning that the benchmarking evaluates only the motif-to-structure assignment; the spectra-to-motif assignment is not benchmarked, as it was validated in the original MS2LDA paper.

The masking and clustering applied to mined Mass2Motifs can indicate if an MAG annotation is helpful or not by comparing the structure size of the annotation with the number of fragments and losses of the optimized Mass2Motif. When a large structure contains only one fragment, it is likely that the annotation or the Mass2Motif is less informative. Completely uninformative MAG annotations are filtered out since they do not share any fragments. We added a section discussing trends that indicate when MAG annotations are more meaningful and when they are less so (see the new Results section on Modelling and Annotation Performance on Library Spectra and Their Interpretation).

Comment 4:
"Supplementary Note 1.2 describes the "optimization" of Mass2Motifs. In the case studies it is mentioned that a particular issue can be circumvented by using this optimized version (the example of Mass2Motif 90). Is this done by default? Are there reasons to not use the optimizations?"

Response 4:
Yes, the optimization is applied by default. Both the optimized and the original ("unoptimized") Mass2Motifs are stored in the output folder, so users can inspect or compare them if needed. While we generally recommend using the optimized versions, there are specific situations where users may prefer to use the original "raw" motifs. The optimization depends on MAG, which in turn relies on spectral embeddings via Spec2Vec. If the relevant substructures are poorly represented or entirely missing from the Spec2Vec training data, the resulting similarities, and therefore MAG may be unreliable. In such cases, automated optimization may discard meaningful patterns, and manual inspection remains a favourable path. We therefore included several plots in the ms2ldaviz app to allow users to perform manual inspection of the quality of the Mass2Motif and to study which features contribute mostly to the Mass2Motif pattern.

Comment 5:
"Selected MotifDB-MotifSets generated with the initial MS2LDA version have been transferred into the new format: Is this a very work-intensive task or are there other reasons that "old" motifs are not available for MS2LDA 2.0? Perhaps we misunderstood this point in the manuscript"

Response 5:
We changed the format of MotifDB to make it compatible with MassQL queries. This required a one-time scripted conversion step, and all existing MotifSets from the initial MS2LDA version have been transferred into the new MotifDB format. The user can directly perform Mass2Motifs – MotifSet matching of all the MotifSets available in MS2LDA 2.0 (Figure 1). We have clarified this point in the revised manuscript to avoid the impression that only a subset of "old" motifs is supported.

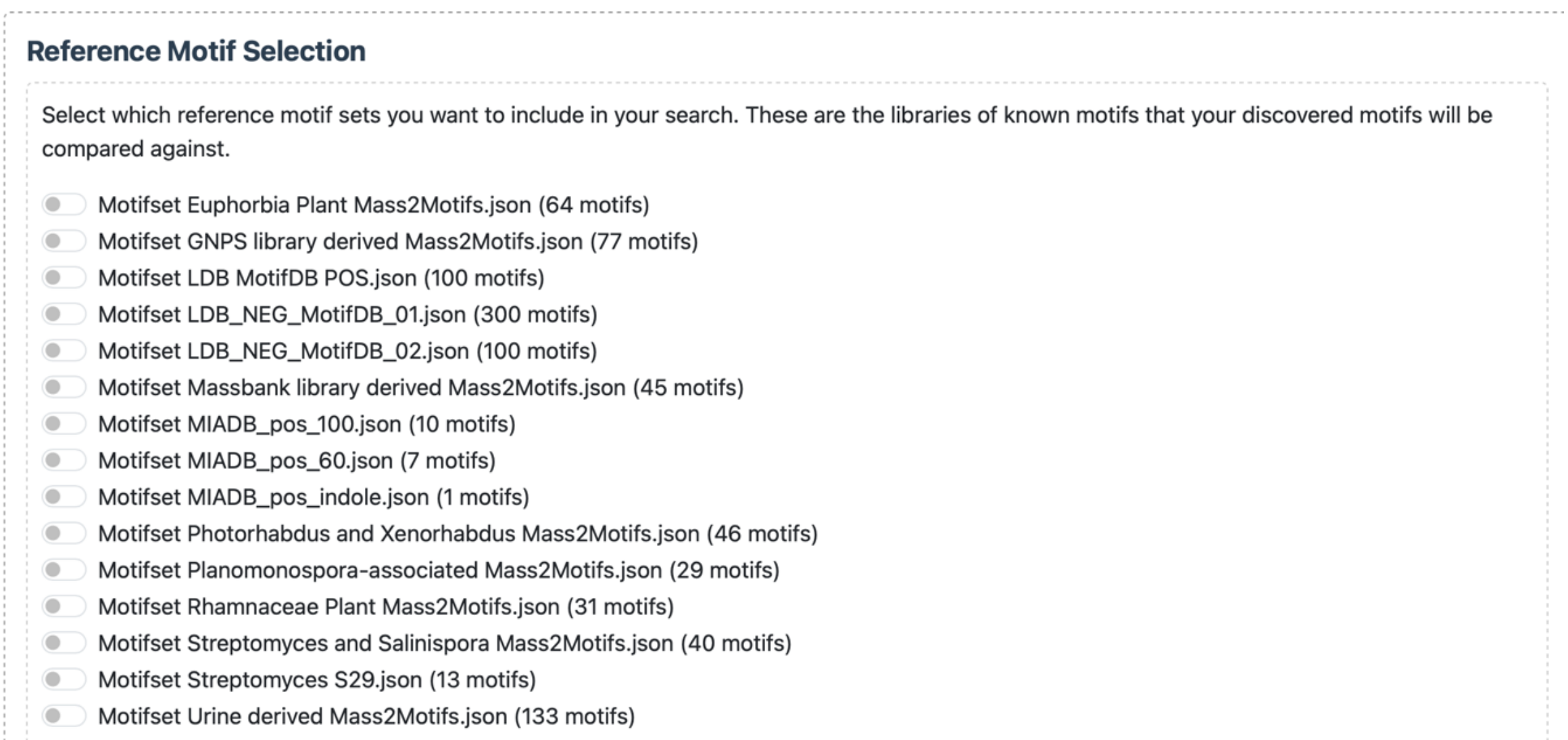

Fig 1. Screenshot from the current app showing all MotifSets available from the earlier version.

Comment 6:
"Parameters for the clustering after Spec2Vec search are not described (distance threshold, number of clusters)"

Response 6:
We apologize for this oversight, and we have now added a table in the Supplementary Information that lists and explains all parameters used for the clustering step after the Spec2Vec search, including the distance threshold and the number of clusters.

Comment 7:
"MAG currently only works for positive ionization mode, this should be earlier stated in the manuscript"

Response 7:
We acknowledge the reviewer's point on that MAG currently only works for positive ionization mode. We now state this limitation explicitly earlier in the manuscript, so that readers are aware of it from the outset.

Comment 8:
"By default, the top five database hits are retrieved as candidate matches": Should be ten, right?"

Response 8:
We had different default values in the app and in the notebooks. As we always used 10 in the notebooks, we have now also changed the default number in the app to 10 for consistency.

Comment 9:
"Mass2Motif 90 associated to the hericenone family, provided a poor annotation (Fig. S15)." Should be S14."

Response 9:
We have corrected the mistake and now refer to Fig. S14.

We thank the reviewers again for their detailed and constructive comments. Their feedback has helped us substantially improve the clarity, contextualisation and introduction of MS2LDA 2.0.

Manuscript ID: NCOMMS-25-56219-T
Title: "Large-scale discovery and annotation of hidden substructure patterns in mass spectrometry profiles"
Authors: Laura Rosina Torres Ortega, Jonas Dietrich, Joe Wandy, Hans Mol & Justin J. J. van der Hooft

We thank the editor for the positive encouragement and acknowledgement that our manuscript has improved upon the revisions. Here below, we will respond to the reviewers' points. Furthermore, we have added another case study to showcase how our current contribution to MS2LDA contribute to accelerating the discovery of novel biological findings. You will find our responses to the reviewers' points here below in green font, alongside key additions we made to the manuscript. Following the changes, we have also updated any references to new sections and Supplementary Notes and Figs. Highlighted sections from the manuscript are in blue font.

Reviewer 1

(a) relevance (meaningful, important to users). While the authors have shown relevance for true positive spike-in of pesticides, it is still unclear how false-positives could be measured.

The MS2LDA concept as introduced and benchmarked in the original study (Van der Hooft et al., 2016) is unsupervised by design. Hence, in the pesticide case study, we can assess true positives (as we know the structures of the spiked-in pesticides), but we cannot assign any false positives, as we cannot readily annotate/identify mass features from the complex matrix. Only with known standards (as was done in Van der Hooft et al., 2016), one can assess statistics like true and false positives; however, how these statistics would translate to experimental (unannotated) data, depends on the biochemical contents of the samples. Besides, the benchmarking of the MS2LDA concept has been done in the original study, in our current study we benchmark Mass2Motif Annotation Guidance (MAG) that is introduced also assess the speed/scalability of our new implementation with various datasets.

(b) representativeness (are the performance metrics broadly accepted and understood by the community?). Here, the authors write "determining whether a MAG annotation captures a distinct substructure remains challenging". From that sentence, it appears that understanding when and if MAG would work is not clear to the authors, let alone benchmarked.

It is unclear to us how the MAG benchmarking connects to representativeness. We would like to stress that we have benchmarked MAG (see section "*Benchmarking of Mass2Motif Annotation Guidance using MotifDB*") and showcase how it facilitates Mass2Motif annotation with help of mass spectral libraries. The quoted text ("determining whether a MAG annotation captures a distinct substructure remains challenging") relates to the section *Modeling and Annotation Performance on Library Spectra and Their Interpretation*, where we continue to write that "In general, higher values for these parameters increase the probability that a MAG annotation reflects a meaningful substructure, with the number of consistent MAG annotations emerging as the most informative feature." As such, the quoted sentence does not relate to the MAG benchmarking. To clarify ourselves, we have changed the quoted text sentence by adding "based on Mass2Motif properties, i.e.:

In summary, determining whether a MAG annotation captures a distinct substructure based on Mass2Motif properties remains challenging.

(c) equity (comparing different systems). The authors still argue that there are no other systems to compare to. The authors argue that compound classification was different than substructure analyses.

Yet, the examples they showed (from alpha-amino acids to sulfonureas or indoles) are very much like definitions of classifications. Hence, the authors missed this important criterion, comparison to other systems.

We understand that this point may be confusing as indeed, in some cases, substructures may be representative for specific compound classes. However, MS2LDA does not predict chemical compound classes such as CANOPUS, as many substructures do not relate to specific classes. Furthermore, MS2LDA can also capture yet unknown scaffolds (and thus chemical compound classes), as it is an exploratory data analysis tool, unlike chemical compound class prediction that requires the training of a supervised approach, as specific endpoints are known (i.e. the classes). We would like to point out to the reviewer that the MS2LDA concept has been benchmarked/validated as a part of the original publication (van der Hooft et al., 2016), using the back then available GNPS and MassBank library spectra, and we do not think doing another benchmark will add substantial value to our current study. Instead, we have put much effort in benchmarking/validating MAG by creating the necessary MotifDB MotifSets see section "*Benchmarking of Mass2Motif Annotation Guidance using MotifDB*"). Yet, we had included several case studies to showcase the strengths of MS2LDA and in particular MAG, where we use some examples that correspond to known chemical compound classes or scaffolds.

We have added the term "exploratory data analysis tool" to the introduction to further clarify this aspect of MS2LDA.

We would like to add that wide-spread used exploratory data analysis tools like Molecular Networking share the same challenge with MS2LDA: there is no ground truth available, and it makes it challenging to assess "success"; at the same time, these unsupervised computational metabolomics tools have been applied in many studies to facilitate biological discoveries. Also, molecular networking groups mass spectra in molecular families that often correspond to chemical compound classes or associated scaffolds; however, as MS2LDA, it is also able to organize and group yet unknown chemistry as it is not supervised/trained to recognize specific classes. Yet, molecular networking and MS2LDA provide complementary insights, as was also highlighted in the original publication and in the current manuscript (Figure 4).

(d) repeatability. (verification and comparison of results across different runs). It is unclear if authors have even tried that test for their system, let alone on different implementations (hardware or software environments)

We would like to invite the reviewer to check our GitHub repository (https://github.com/vdhooftcompmet/MS2LDA). Our codebase is available with a substantial number of tests, as Reviewer 2 also points out. Also, as can be seen in the documentation (https://vdhooftcompmet.github.io/MS2LDA/), and MS2LDA is tested and can be run on the main OS (Linux, MacOS, Windows). To also clarify this in the main text, we have added the above details on the operating systems and documentation to the "Code availability" section.

(e) Verifiability. (users should be able to verify the accuracy of benchmarking results). Here the authors argue that "no ground truth exists" and that "...the number of fragments, the size of the shared substructure, and the number of recommended structures, provide helpful tendencies but do not yield strict thresholds." Hence, users cannot verify the usefulness of the tool, because MAG is just a guidance, a tool for ...estimating or guessing substructures, without confidence thresholds. That is not instilling confidence in the community, the reviewer would like to argue.

We would like to emphasize that the original publication has been cited >400 times by now, and the MS2LDA concept has been proven itself to facilitate new biological discoveries. We have added a paragraph in the introduction with several examples of such discoveries (see also here below).

To answer the question about how both MS2LDA and MAG provides guidance, MS2LDA comes with probability values from the LDA model that facilitate confidence assessment, together with the visualization plots that are available within the MS2LDAviz app. We have also added the overlap score as a means to filter the Mass2Motif-associated spectra to more confident links. With MAG, the number of matched structures and their consistency in overlapping substructures guide the user in confidence of the suggested annotations, i.e., the more structures and consistency, the better. Hence, we think we do empower users with the necessary means to assess the quality of the exploratory data analysis results.

Regarding "users cannot verify usefulness of the tool", we have shown the added value of MAG by the mutiple case studies already included in the manuscript. However, to further validate MAG, we have added another case study, by reanalyzing the recent amphibactin siderophore study to assess whether the manual identified Mass2Motifs can be automatically annotated by MAG. Here, we highlight how MS2LDA 2 with its MAG capabilities can accelerate biological discoveries.

Added paragraph with impact of MS2LDA on untargeted metabolomics studies:

Due to its unsupervised nature, MS2LDA has already facilitated substantial novel biological discoveries. For instance, its application revealed the key role of homogentisic acid as a precursor for biosynthesis and biotransformation of the antibiotic trimethoprim in pigmented bacteria, providing new opportunities for treatment of infections[16]. In another study, MS2LDA identified distinct Mass2Motis in polyketides with cytotoxic and antitumor properties, discriminating key properties that may influence their antitumor properties, and facilitating focusing on novel structural analogs amongst the currently 500 known acetogenins[17]. Finally, MS2LDA has been an integral part of the unsupervised workflow that resolved the first siderophore hydrolysis degradation mechanism of amphibactin in co-cultured marine bacteria[18].

Added manuscript section on the new case study:

Case Study on Accelerating Biological Discoveries with MAG-empowered MS2LDA

To demonstrate end-to-end utility and verifiability, we reanalyzed the publicly available dataset from Monge-Loría et al. (2025)[18], who reported the first degradation of amphibactin, a widely abundant siderophore in the ocean, by *Microbulbifer* spp. The original study identified a shared (motif 531) between amphibactin F (m/z 876.529 [M+H]+) and a degradation product (m/z 617.412 [M+H]+), supported by diagnostic fragments: 86, 114, 131, 191, 278 and 450, containing a N-acetyl-N-hydroxy-ornithine substructure. Due to its chelating properties, this annotation was biologically- plausible and supported the interpretation of *Microbulbifer* spp. mediated amphibactin degradation.

Using MS2LDA 2.0 with the preprocessing and parameters reported in the original study, we recovered a corresponding Mass2Motif containing both features. MAG allows to directly link the Mass2Motif to the N-acetyl-N-hydroxy-ornithine substructure as it is present in all its recommended structures. Furthermore, the Mass2Motif optimization resulted in two remaining fragments: 131.08 and 86.06, the same key diagnostic fragments recognized earlier (see S.I. section 1.2 for details on Mass2Motif optimization). At the feature level, the original study reported 68 features associated with motif 531 at a motif–spectra probability threshold of >0.1. Our independent reanalysis recovered 32 of these 68 features, indicating substantial concordance despite independent inference and the probabilistic nature of topic modelling (S.I. section 5).

We further examined bromotryptamine-related metabolites reported in the same study (bromotryptamine, N-formyl bromotryptamine and N-acetyl bromotryptamine). These features co-occurred in motif_34 and motif_129; however, after MAG optimization, each motif was supported by only a single retained fragment, leading to weak and nonspecific substructure recommendations. This potentially reflects limited diagnostic fragmentation information, and possibly incomplete reference coverage in current public libraries. Importantly, both motifs still consistently grouped the brominated features, a relationship not explored further in the original work, and they could be used to mine other datasets for their presence as well.

Together, this case study demonstrates the added value of MAG and its Mass2Motif optimization. At the same time, it highlights cases where MAG is insufficient for confident automated annotation of motifs; however, we do observe how structurally related molecules are still associated to such motifs. Moreover, MS2LDA 2.0 recovers previously reported biological insights on an external dataset, with MAG substantially accelerating substructure hypothesis generation when diagnostic signals are present.

Added Supplementary Note on new case study:

Case Study on Accelerating Biological Discoveries with MAG-empowered MS2LDA

To investigate the impact of MAG on accelerating biological discoveries, we re-analyzed the previously reported siderophore degradation case study[9], and we investigated if Mass2Motifs related to Amphibactin F-like and bromotryptamine-like structures would be produced and annotated by MS2LDA 2.0. Besides reproducing previously found Mass2Motifs, we were especially interested in their MAG annotation as this is one of the key enhancements of the new version. For the first example, Amphibactin F and its degradation product shared a manually annotated motif in the original paper. From our results, these two metabolic features were found to be connected to motif_66. (see Fig. S29). The Mass2Motif optimization highlights the common fragments of *m/z* 86.06 and *m/z* 131.08 (in orange in Fig. S29. Interestingly, the three recommended structures also contain the manually annotated N-acetyl-N-hydroxy-ornithine substructure, showcasing the acceleration in substructure discovery through MAG by saving time on manual Mass2Motif optimization and annotation through human expert knowledge.

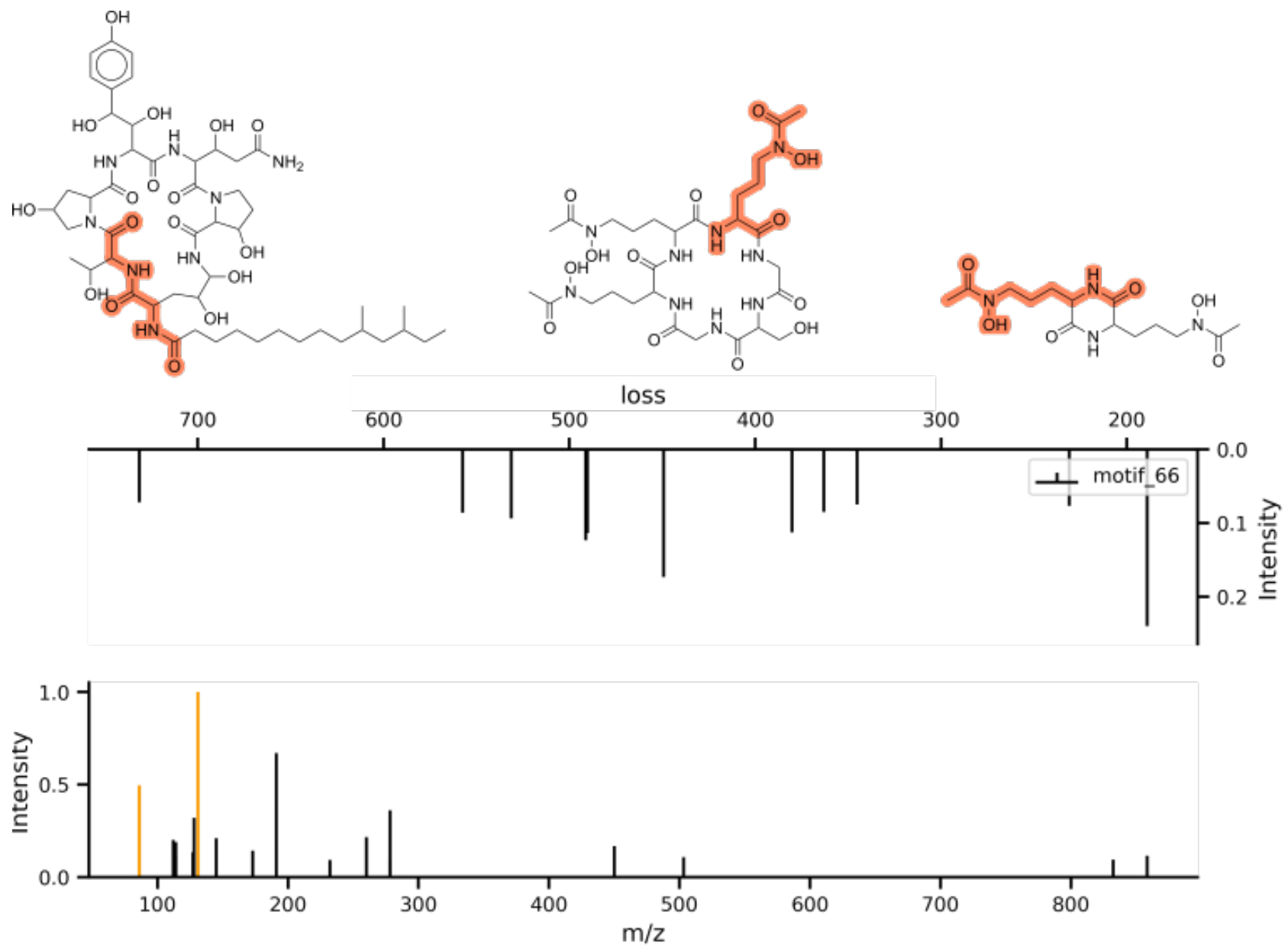

Fig. S29. *Mass2Motif 66 is associated with a N-acetyl-N-hydroxy-ornithine substructure. The top panel shows the three recommended structures MAG returned. The bottom panel displays the fragments associated with this Mass2Motif as a standard MS/MS spectrum. The orange highlighted fragments are consistently observed in the recommended structure. Mirrored above the fragments are the corresponding neutral losses, arranged from right to left in increasing mass difference from the precursor ion.*

In case of the other example, bromotryptamine-molecules (bromotryptamine, N-formyl bromotryptamine and N-acetyl bromotryptamine) motifs were not further explored in the original study. In our MS2LDA run, we found motif_34 and motif_129 were associated with these three features, showing how this motif can represent a unique substructure. However, the MAG annotation for both motifs returned unrelated (sub)structures. There is only one optimized fragment in both cases that represents all the recommended structures. In mass spectra data, many kinds of structures can yield a single similar fragment, so here MAG cannot give a lead for substructure annotation. This highlights the need of multiple optimized fragments for a higher chance of obtaining a good annotation (as in the previous example). Other potential reasons for an inconsistent annotation given by MAG are the poor coverage of bromotryptamine like compounds covered in the database or a missing and dominant fragmentation pattern for a substructure. Nevertheless, the MS2LDA 2.0 Mass2Motifs can connect structurally related molecules. Using tools like MassQL, the initial MS2LDA-inferred mass spectral patterns could be further refined, also using human expert knowledge.

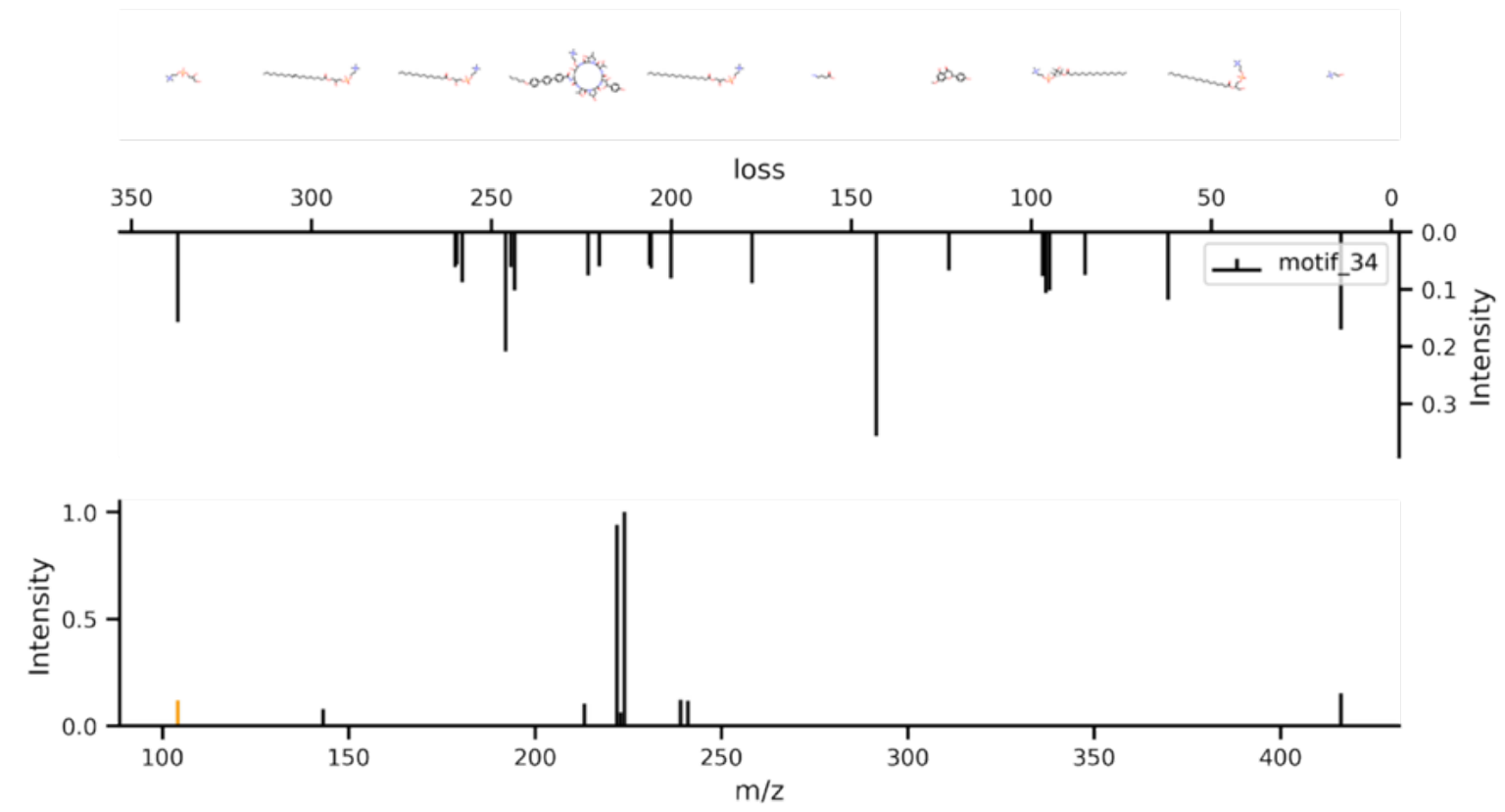

Fig. S30. *Mass2Motif 34 is associated with bromotryptamine-like spectral features. The top panel shows the only structure from MAG. The bottom panel displays the fragments associated with this Mass2Motif as a standard MS/MS spectrum. The orange highlighted fragment is consistently observed in the recommended structure. Mirrored above the fragments are the corresponding neutral losses, arranged from right to left in increasing mass difference from the precursor ion.*

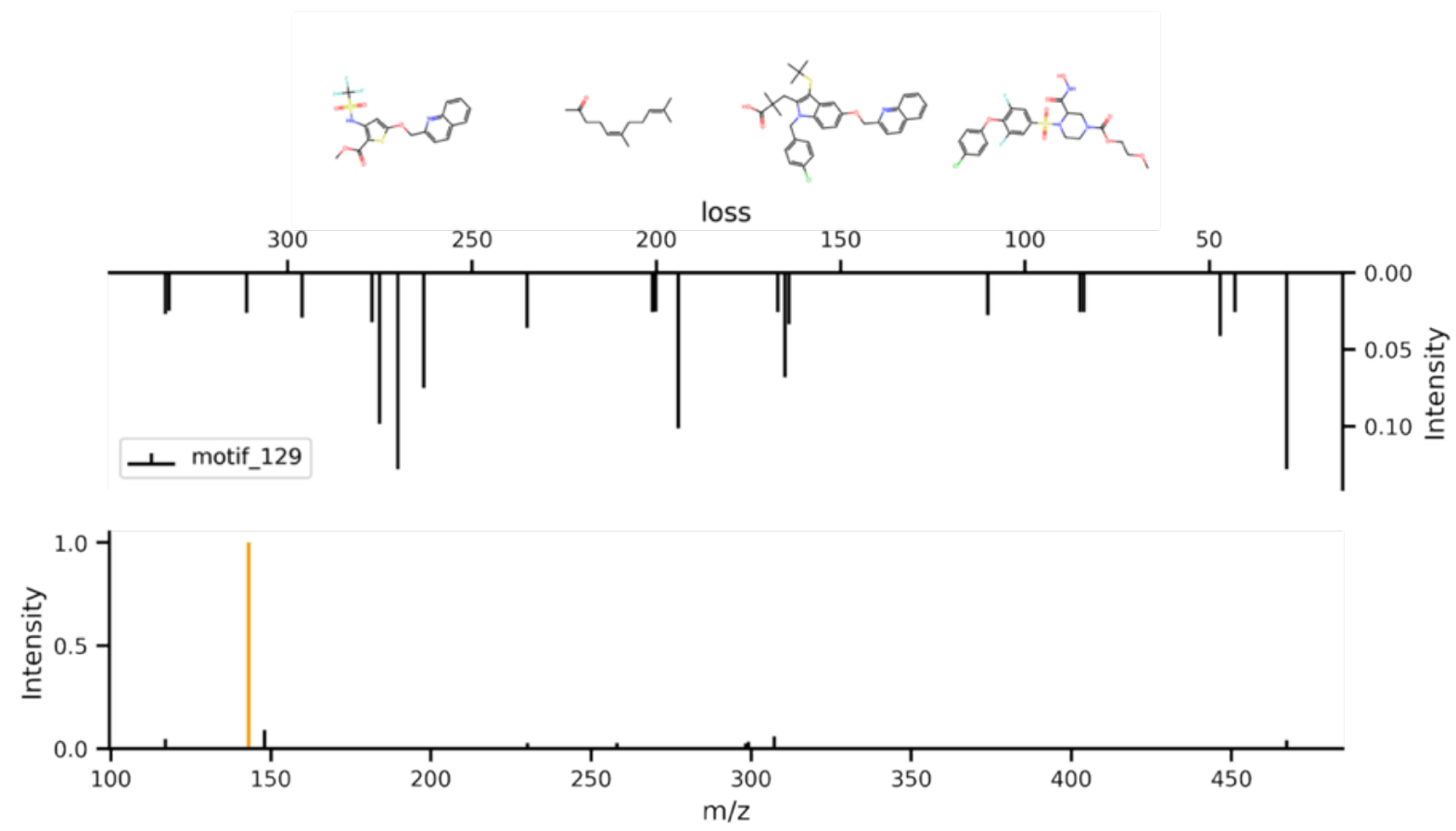

Fig. S31. *Mass2Motif 129 is associated with bromotryptamine-like spectral features. The top panel shows the only structure from MAG. The bottom panel displays the fragments associated with this Mass2Motif as a standard MS/MS spectrum. The orange highlighted fragment is consistently observed in the recommended structure. Mirrored above the fragments are the corresponding neutral losses, arranged from right to left in increasing mass difference from the precursor ion.*

(f) Transparency (details of benchmark designs, implementations, configurations used). The authors argue that for this reason, NIST23 could not be used. Instead, other datasets were used that were also not easily accessible (e.g. hosted on a benchmarking cluster site). The reviewer understands that community users could go at lengths to retrieve the data themselves, but without classic benchmarking results, (true/false positives, true/false negatives and corresponding ROC results). The authors argue that they did not try to find true/false positives etc because it is a discovery engine. Yet, the examples were based on predefined classes (i.e. pesticides, indoles etc). The reviewer fails to acknowledge that the authors showed any clear case of discoveries or benchmarking for the ability for discoveries.

We agree that transparency is key for open science and reproducible science, but we disagree with the statement that datasets used in our study were not easily accessible. If we understand correctly then "hosted on a benchmarking cluster site" refers to the GNPS platform. As the reviewer may not be familiar with common procedures and resources in our field, we attached some Data availability sections from other publications in Nature Methods and Nature Communications (not by ourselves) that are similar in style as our statement:

"The four reference MS/MS libraries used for evaluation can be freely downloaded at https://mona.fiehnlab.ucdavis.edu/downloads. The MS/MS spectra of five newly discovered compounds can be accessed on GNPS (CCMSLIB00005467952 and CCMSLIB00005716808) and https://www.nature.com/articles/s41592-021-01303-3#MOESM10."

From https://www.nature.com/articles/s41592-023-01850-x

"The NIST20 and NIST23 data used in this study are available under restricted access as paid standard reference databases, access can be obtained by purchasing from NIST Mass Spectral Library. The GNPS data used in this study are available at GNPS Mass Spectral Libraries. The MoNA, CASMI 2016, CASMI 2017, and EMBL-MCF 2.0 data used in this study are available at Mass Bank of North America."

From https://www.nature.com/articles/s41467-025-66060-9

The second comment made by the reviewer, is about not producing classic benchmarking results (including a ROC). The reviewer is correct that we cannot produce true/false positives, true/false negatives and corresponding ROC results for unsupervised substructure discovery. Instead, we have looked for analyses that demonstrate MS2LDA 2's added capabilities and we have included the pesticide case study using spiked-in molecules, allowing us to assign true positives (a spectrum-molecule combination that got a Mass2Motif associated that correctly describes part of its structure), and false positives (a spectrum-molecule combination that got a Mass2Motif associated that does not describe part of its structure). However, true and especially false negatives are ill-defined in unsupervised discovery, as we cannot know [from the unknowns in a complex mixture] what should have been matched.

Finally, regarding the use and influence of the so-called "predefined classes", we would like to indicate that we see the role of the "predefined classes" as demonstration in our case studies, i.e., to highlight current capabilities of our framework, for which we required known molecules or annotated spectra. In terms of performance for various classes (i.e., the "influence they have on the algorithm"), we would like to clarify that this is mostly determined by the fragmentation behaviour of the molecules – something we only start to understand and model for a limited number of compound classes.

(g) Scalability (adaptable for systems with different resource levels). While the authors argue that their system is now more scalable than their version before, 14-times faster, it is unclear how the tool would scale on different computer systems (i.e. how users would use it).

We have worked on our documentation (https://vdhooftcompmet.github.io/MS2LDA/), where different operating systems are specified.

In the main, we have indicated how our scalability tests were performed in the "*Integration with other computational mass spectrometry frameworks and computationally efficient topic modelling*" session.

The scalability study was performed on a server, but with a rather low-spec setup. We tested the software with one and four CPU cores, while most standard laptops often have eight or more CPU cores, which should further improve performance. For users analyzing their samples, commonly containing ~5000-10000 tandem mass spectra, the running time of the algorithm should not pose any issue. We conducted the scalability study on a server as scalability becomes even more important when analyzing large(r) datasets and servers are commonly the user's choice. Furthermore, the usage of servers for such larger datasets is generally recommended for mass spectrometry pipelines (and having or getting access to servers is not that difficult) [https://link.springer.com/article/10.1186/s13321-023-00724-w]. Also, we note that workflow management tools, such as Snakemake, are ideal for scalability, reproducibility, and easy deployment to different cluster, cloud, or server environments. Finally, we acknowledge that there still may be room to further enhance MS2LDA 2's performance on server CPUs to run MS2LDA on very large datasets. We add that the analysis and visualization of such big data may pose novel challenges, in particular, we notice that the current MS2LDAviz app (as well as the human capacity to take in information) is not designed for very large datasets (100,000 metabolite features or more).

(h) end-to-end performance of the overall system, rather than individual components: The authors fail to address this point, as they argue that their discovery system cannot be benchmarked in this way, and scales would depend on the different types of substructures to be found.

We have tested the overall system on various operating systems (see also answer to point d). It is indeed correct that the performance of MS2LDA is dependent on the different types of substructures to be found, or, more precisely, to be expected, as MS2LDA is an exploratory data analysis tool. We would like to refer to our previous answers that highlight how the MS2LDA concept has been benchmarked/validated before, and how MAG has been benchmarked/validated in our current study. We also refer to point e) on verification, where we do a reanalysis of a study that used MS2LDA v1 to assess the added value of MAG that is only available in MS2LDA v2.

Reviewer 2 (and 3)

The manuscript was substantially refined and almost all of our comments adequately addressed. We only have a few minor suggestions that do not require another round of answers.

In the answers and also the revised manuscript the benefits of the new version, especially MAG, are now clearer presented. We think that the last point from the first answer, reducing the time for manual inspection of motifs from hours/days (?) to minutes, could also be included explicitely into the manuscript, perhaps at the end of the Introduction or beginning of the Results. This is indeed very impressive.

We thank the reviewer(s) for the positive words on our efforts in making our manuscript clearer. We have looked at our manuscript and we think the sentence under the "*Benchmarking of Mass2Motif Annotation Guidance using MotifDB* " session captures the intended time reduction on annotation of Mass2Motifs sufficiently, also together with an important condition:

"When relevant structures are found, this substantially accelerates and simplifies the structural annotation process."

We see now that optimization of motifs is always performed. This does however sound different in the description of one case study; this might need to be adapted to avoid confusion: "This issue can be circumvented by using the optimized fragments of the Mass2Motif (Supplementary Notes 1.2)."

We have changed the sentence to reflect that optimized Mass2Motifs are always available:

However, we note that as a part of MAG Mass2Motif optimization takes place and the optimized fragments of the Mass2Motif could be used instead to improve generalization of the discovered fragmentation pattern (see Supplementary Notes 1.2 for details on Mass2Motif optimization).

The new supplementary table on parameters is much appreciated, we could however not find the parameters for agglomerative clustering. We specifically refer to the following sentence in the manuscript: "This behavior is identified by applying agglomerative clustering (scikit-learn version 1.5.1)"

Thank you for highlighting the need for clarity regarding the agglomerative clustering parameters, which are a central aspect of the Mass2Motif Annotation Guidance recommendation process.

To address this, we have specified in the table that the similarity metric used is Cosine Similarity, from which we derive the cosine distance. Additionally, the linkage criterion for cluster formation is always set to "complete". These details have now been included for in the table.

	Cosine_ similarity	float	0-1	0.9	For calculating the cosine distance for the agglomerative clustering (= 1 – Cosine_similarity). Therefore, number of clusters is set to None and the algorithm used to determine the clusters is hardcoded to "complete"

Reviewer 2 (and 3) - Remarks on code availability:

Code is looking ok, well-documented, tests etc

We are glad that the reviewers appreciate our efforts in making our code well-documented and well-tested to promote its use and reuse.

Point by point answer

Manuscript ID: NCOMMS-25-56219-T
Title: "Large-scale discovery and annotation of substructure patterns in mass spectrometry profiles"
Authors: Laura Rosina Torres Ortega, Jonas Dietrich, Joe Wandy, Hans Mol & Justin J. J. van der Hooft

Remaining reviewer comments

Reviewer 2:
The only point where it makes more sense to answer in the manuscript than to the reviewer only, is (c). The answer to the reviewer says, "we are doing this, and here are the differences to CANOPUS for predicting compound classes" -- but that should be explained *in the manuscript instead*. In particular, since a very similar issue is coming up again in (f) for predefined classes.

Our Response: we thank the editor and reviewers for their careful evaluation of our manuscript and the given constructive comments. The main point raised by reviewer #2, was to add the answer (c and in some extend f) of the previous response to editors document regarding the differnce between the chemical class prediction and substructure approach, which we have adressed in the new version of our manuscript.

This is explained as a paragraph in the introduction (lines 48-60):

"An alternative route to annotate metabolite features is to predict their chemical compound class terms with tools such as MolNetEnhancer[15], CANOPUS[16], which assigns spectra to classes within a predefined ontology, or through analogue search to library spectra with predefined classes with tools like MS2Query[17]. Such class ontology-based annotations could be combined with maximum common subgraph analysis to derive shared substructures. However, these ontology-driven strategies are limited by the resolution of the class hierarchy and often return trivial scaffolds for structurally diverse classes, while many relevant substructure moieties or functional groups, such as glycosylations, phosphate groups or indole rings, can recur across multiple chemical classes, and therefore not captured based on this class-prediction logic. As such, unsupervised substructure-level discovery approaches such as MS2LDA that operate directly on the mass fragmentation spectra are complementary to both full-structure annotation and chemical compound class prediction."
